# Spectroscopic and photochemical evaluation of stereochemically biased 3′-substituted spiropyran photoswitches†

**DOI:** 10.1039/d4ra07750d

**Published:** 2024-11-21

**Authors:** Vojtěch Boháček, Tereza Erbenová, Jakub Dávid Malina, Marie Kloubcová, Michal Šmahel, Václav Eigner, Jiří Tůma

**Affiliations:** a Department of Organic Chemistry, University of Chemistry and Technology Prague Technická 5, Prague 6 Prague 166 28 Czech Republic tumaa@vscht.cz; b Department of Solid State Chemistry, University of Chemistry and Technology Prague Technická 5, Prague 6 Prague 166 28 Czech Republic

## Abstract

Three series of spiropyran photoswitches with an auxiliary chiral centre at position 3′ of the indoline unit were synthesized. Using one example, a novel methodology for synthesis of an optically active spiropyran photoswitch with a defined chirality at position 3′ is demonstrated. Furthermore, a new acid-mediated strategy for spiropyran purification affording moderate to excellent yields (up to 96%) is reported herein. Relative diastereomeric ratios of the prepared spiropyrans were evaluated using NMR spectroscopy in five different solvents (*syn* : *anti* up to 21 : 79) and their photoswitching properties determined by UV-vis spectroscopy. It was found that substitution at position 8 of the chromene subunit notably accelerates the photoswitching process.

## Abstract 2

A library of 18 optically biased spiropyran photoswitches were synthesised and evaluated using NMR and UV-vis spectroscopy. A strategy for synthesis of optically active spiropyrans and a new purification method for such compounds are also reported.

## Graphical abstract

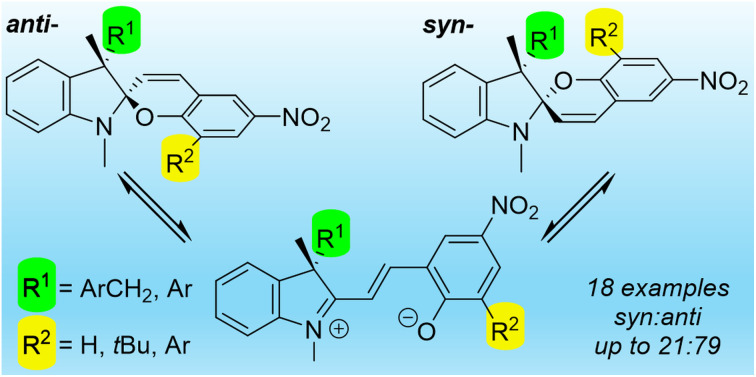

## Introduction

**Scheme 1:**
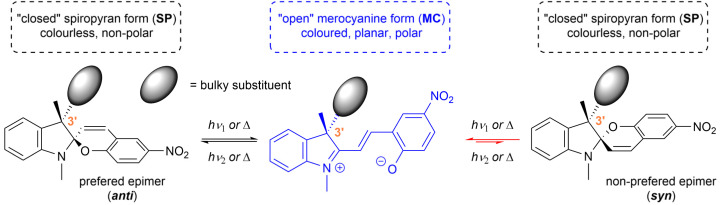
Equilibrium between spiropyran diastereomers.

Spiropyrans are well-known photoswitchable compounds with unique properties. The key characteristic of spiropyrans is the very nature of their photoswitching transformation. In contrast to common photoswitches (*e.g.*, stilbenes and azobenzenes), upon irradiation, they not only change their geometry, but also undergo a dramatic transformation in their polarity. The non-polar, colourless spiropyran (SP) arrangement with two perpendicular subunits (indoline and chromene part) can be easily turned into a planar, conjugated, zwitterionic merocyanine form (MC), which is deeply coloured (Scheme 1).^1,2^ Alongside photoinduced activation, spiropyran switching can also be triggered by other stimuli, such as thermochromic,^3^ acidochromic,^4^ electrochromic,^5^ or mechanochromic^6^ impulse. Thanks to this fast and wide tunability of their molecular scaffold, spiropyrans have found numerous applications in materials science^7^ including, but not limited to, modern drug delivery systems,^8^ materials facilitating molecular motion,^9^ photochromic fluorescent probes,^10^ chemosensors of metal ions,^11^ photochromic liquid crystals,^12^ and modification of solid surfaces.^13^

The very nature of the currently emerging applications of spiropyrans calls for optically pure compounds that would govern the next evolutionary step of spiropyran-based materials. An optically pure spiropyran represents a cornerstone of a new dimension of spiropyran research. Materials based on such photoswitch could facilitate numerous new applications, such as tunable stereoselective sensing, formation of switchable oriented chiral domains in bulk materials, optically active modifiable surfaces, light-driven selective recognition of chiral molecules, and likely many others.^7^

Generally, the scaffold of the spiropyran photoswitch is chiral due to the presence of the spiro-carbon. It is, therefore, theoretically feasible to isolate their respective enantiomers, *e.g.*, by dynamic enantioselective crystallization,^14^ or *via* HPLC with a chiral stationary phase.^15,16^ In solution, however, spiropyrans undergo a dynamic equilibrium of the spiropyran and merocyanine form, which leads to undesired spontaneous racemization^16,17^ leaving these compounds unreliable for the outlined applications requiring optically pure species.

In order to stabilize the optical purity of the spiropyran backbone, it is possible to incorporate a defined centre of chirality in the position 3′ of the indoline subunit, *i.e.*, using two different substituents in this position (*e.g.*, methyl and bulky alkyl or aryl groups). This way, the overall structure, due to the presence of two chiral centres, shifts from a racemic mixture to diastereomeric equilibrium undergoing between two epimers (Scheme 1). Such stereoisomers differ in their free energy and, therefore, their relative ratio. For example, if the energy difference between the two epimers is 12 kJ mol^−1^, their ratio is equal to 99 : 1 at 25 °C. This concept was originally investigated by Gruda *et al.* in 1978 (ref. 18) and later by Eggers *et al.* in 1997.^19,20^ However, only seven compounds in total, exhibiting rather modest diasteromeric excess (*syn* : *anti* = up to 29 : 71), were reported. To the best of our knowledge, this approach to optically biased spiropyrans remained unexplored any further until 2018, when Perry *et al.* published their work on microwave-promoted synthesis of spiropyrans with varying substitution in the position 3′.^21^ They reported a library of 15 compounds bearing several alkyl and arylmethyl groups mostly with respect to their steric hindrance on the individual spiropyran epimers. The epimeric ratio was evaluated using ^1^H NMR in CDCl_3_ and the stereochemical preference (*syn* : *anti*) was determined by NMR NOESY experiments that (in most cases) showed clear prevalence of the *anti*-configuration over *syn*-. The best epimeric ratio at ambient temperature was found for a combination of methyl and 2-bromophenylmethyl moieties in the position 3′ (*syn* : *anti* = 14 : 86). In their follow-up work in 2020 (ref. 22) and 2023,^23^ respectively, Perry *et al.* reported further progress on the synthesis of non-symmetrically substituted spiropyrans but put very little emphasis on the stereochemical aspect of the additional chiral centre in the position 3′.

**Fig. 1:**
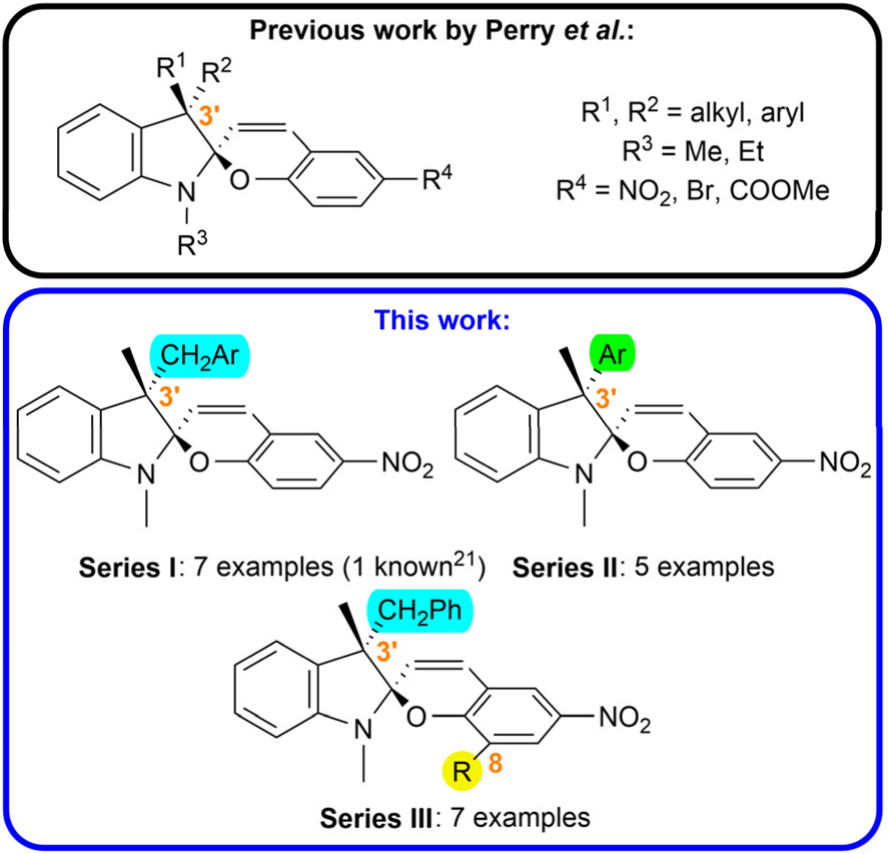
Aim of study.

In this work, we focused on the synthesis of a library of spiropyran compounds with varying substitution in the position 3′. We prepared three series of photoswitches (Fig. 1): (i) we broadened the scope of arylmethyl-substituted compounds investigated by Perry *et al.*^21^ studying the effect of electron donating and electron withdrawing groups (CH_3_, CH_3_O, CF_3_) in the position 3 and 4 of the aromatic moiety (Series I); (ii) we synthesized a series possessing an aryl unit in the position 3′ instead of more flexible arylmethyl functionality (Series II); (iii) we introduced a new structural pattern by incorporation of an auxiliary bulky group (*t*Bu, Ar) in the position 8 of the chromene subunit alongside a benzylic moiety in the position 3′ to increase the steric hindrance between these two groups in the *syn*-epimer (Series III). To the best of our knowledge, all the prepared compounds except for Ia have not been reported up to date.

The kinetic rates of photochemical switching of all the prepared spiropyrans were evaluated using UV-vis spectroscopy. The molecular isomerization from SP to MC form was initiated by UV light (*λ* = 365 nm) while the backwards process was triggered by green light (*λ* = 522 nm). The *syn* : *anti* ratio of the prepared spiropyrans was determined by NMR spectroscopy at 25 °C in various solvents.

As the *syn* : *anti* ratio reflects only relative stereochemical arrangement, all the target compounds have been synthesized in a non-stereoselective manner, *i.e.*, without specification of absolute configuration in the position 3′. Thus, every compound prepared holds a total sum of four diastereomers. However, we also report one compound where we successfully applied our own developed method of stereoselective separation using co-crystallization of a corresponding 3*H*-indole precursor with (–)-camphor-10-sulfonic acid. This way, pure (*R*)-enantiomer of the 3*H*-indole was yielded and subsequently transformed into the target spiropyran. The absolute configuration of the compound was proven by X-ray crystallography. We also developed and successfully applied a new purification method for isolation of spiropyrans using their acidochromic properties. We believe that both of our separation/isolation methods represent a strong standpoint that can serve the spiropyran research community going forward.

## Results and discussion

### Synthesis

This chapter summarizes the applied synthetic pathways towards the target spiropyrans of the Series I–III, and the description of the outlined isolation/purification methods. Detailed experimental protocols and spectroscopic data are available in the ESI.†

#### Series I

**Scheme 2:**
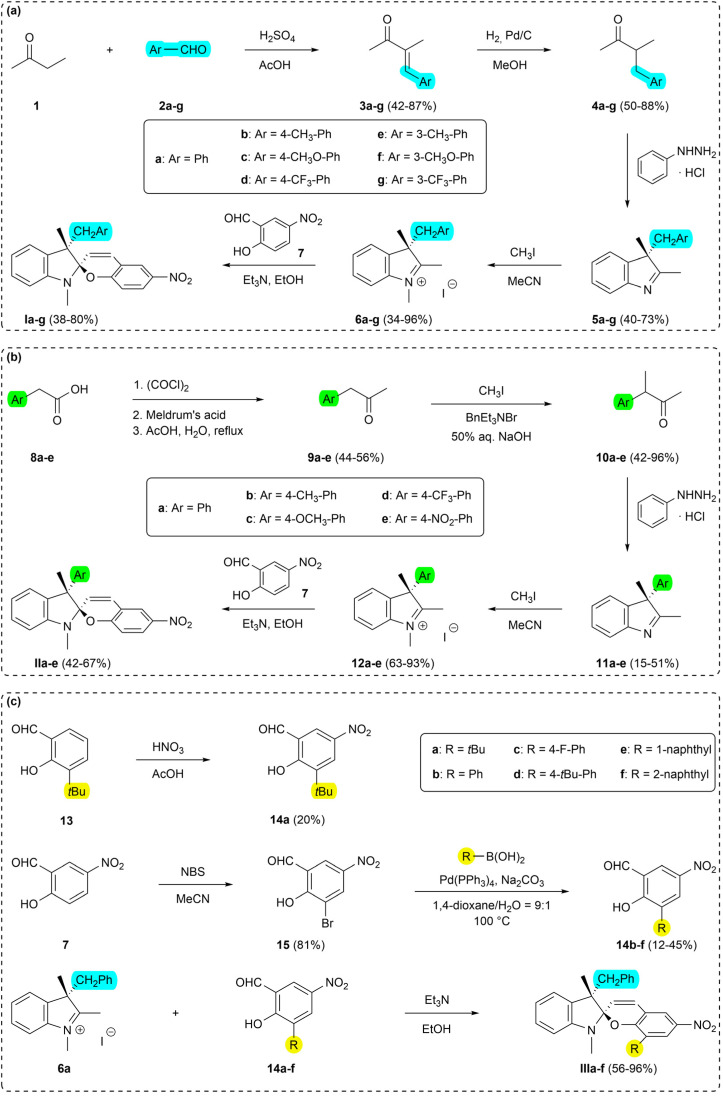
Synthesis of the target spiropyrans: (a) Ia–g (Series I), (b) IIa–e (Series II), (c) IIIa–f (Series III).

Synthesis of the Series I started from butanone (1), which was coupled with the corresponding arene carbaldehydes 2a–g in the presence of sulfuric acid yielding α,β-unsaturated ketones 3a–g that were subsequently reduced by H_2_ gas on Pd/C catalyst affording aryl ketones 4a–g.^24,25^ Ketones 4a–g underwent Fischer condensation with phenylhydrazine hydrochloride to provide 3*H*-indols 5a–g. The prepared 3*H*-indols were methylated and consequently coupled with 2-hydroxy-5-nitrobenzaldehyde (7) in the presence of triethylamine yielding the target spiropyrans of the Series I (Scheme 2a).^26^

#### Series II

Synthesis of the Series II was carried out starting from 4-substituted arylacetic acids 8a–e, which were first transformed to the corresponding methyl ketones 9a–e by the means of 2,2-dimethyl-1,3-dioxane-4,6-dione (Meldrum's acid) followed by acid-mediated hydrolysis.^27–29^ The resulting arylacetones 9a–e were methylated *via* phase-transfer catalysis to yield the intermediates 10a–e.^30^ They were subsequently transformed into the target spiropyrans using analogous procedures as for the Series I, *i.e.*, Fischer condensation with phenylhydrazine hydrochloride, methylation, and condensation with hydroxy aldehyde 7, yielding compounds IIa–e (Scheme 2b).^26^

#### Series III

Synthesis of the Series III was based on tetrasubstituted aromatic hydroxy aldehydes 14a–f. *Tert*-butyl-substituted derivative 14a was prepared by nitration of 3-*tert*-butyl-2-hydroxybenzaldehyde (13).^31^ Key bromo precursor 15 was obtained by substitution of hydroxy aldehyde 7 with *N*-bromosuccinimide (NBS).^32^ The remaining hydroxy aldehydes 14b–f were yielded using Suzuki cross-coupling reaction of the bromo aldehyde 15 and corresponding arylboronic acids.^33^ The target spiropyrans IIIa–f were obtained by condensation of the precursors 14a–f with iminium salt 6a (Scheme 2c).^26^

#### Isolation of an optically pure spiropyran precursor

**Fig. 2:**
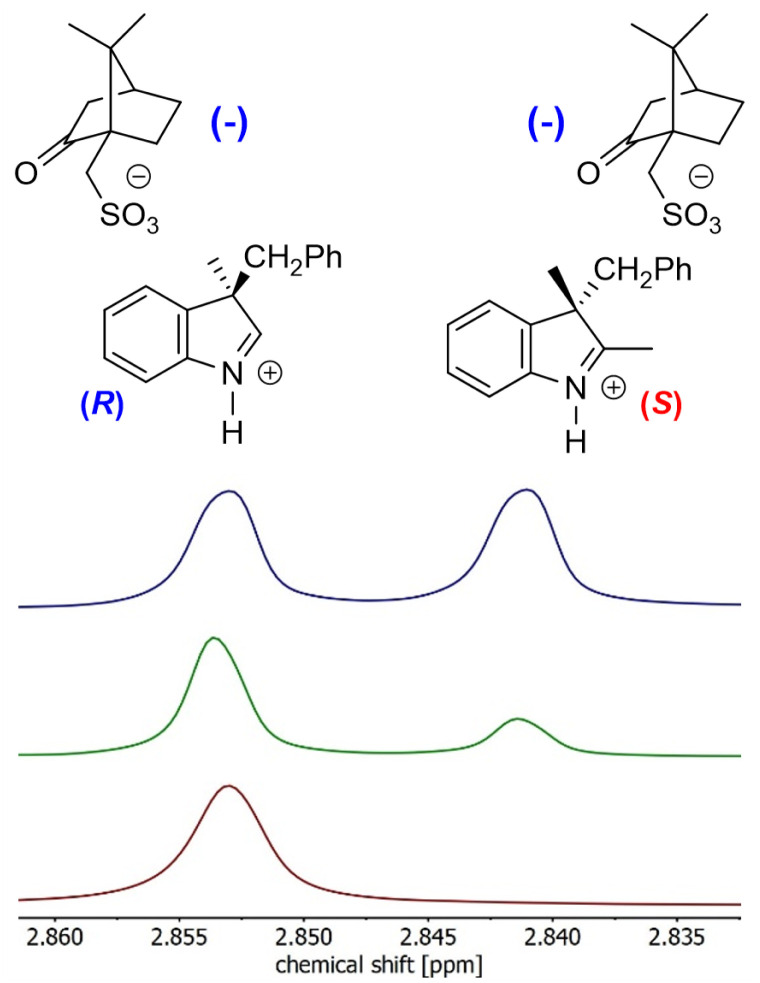
NMR analysis of diastereomeric co-crystallization: (i) initial 50 : 50 mixture (blue); (ii) 74 : 26 mixture after the 1st recrystallization (green); (iii) optically pure mixture after the 2nd recrystallization (maroon). Full NMR spectra are shown in the ESI.†

For the purpose of the outlined study, all the spiropyrans were synthesized with no stereocontrol over the auxiliary chiral centre in the position 3′. However, the ultimate goal – isolation of an optically pure spiropyran – can be achieved only if the 3′ stereocentre exhibits defined chirality. Aside from stereoselective synthesis and chromatographic separation on a chiral stationary phase, optically pure materials can be obtained, *e.g.*, by using co-crystallization with an optically pure additive. We envisioned that the 3*H*-indole precursors 5 and 11 are the most convenient targets for co-crystallization as they have basic character and thus can be captured by chiral acids to form iminium salts that should be suitable for recrystallization. As a model compound, we selected the 3′ benzyl-substituted 3*H*-indole 5a (1.18 g; 5.0 mmol) and mixed it in 1 : 1 ratio with (–)-camphor-10-sulfonic acid (1.16 g; 5.0 mmol) in EtOH. Upon evaporation, crystalline compound was obtained, which was subsequently recrystallized from EtOAc/MeCN mixture. The progress of the recrystallization was followed by ^1^H NMR. Two isolated peaks for the two diastereomers were found for the methyl group in the position 2 of the 3*H*-indole (singlet at *δ* = 2.84 and 2.85 ppm, respectively) in CD_3_CN. Other solvents tested (CDCl_3_, DMSO-*d*_6_, acetone-*d*_6_, CD_3_OD) showed no baseline differentiation of signals at 400 MHz. After the first recrystallization, 702 mg of 74 : 26 diastereomeric mixture was found. Second recrystallization using the same solvent mixture yielded 225 mg (19%) of optically pure salt (Fig. 2). The free base of 3*H*-indole 5a was subsequently released by treatment with aq. NaHCO_3_ followed by extraction into dichloromethane. 101 mg (90%) of optically pure 3*H*-indole 5a was obtained this way. Experimental details are available in the ESI.†

**Fig. 3:**
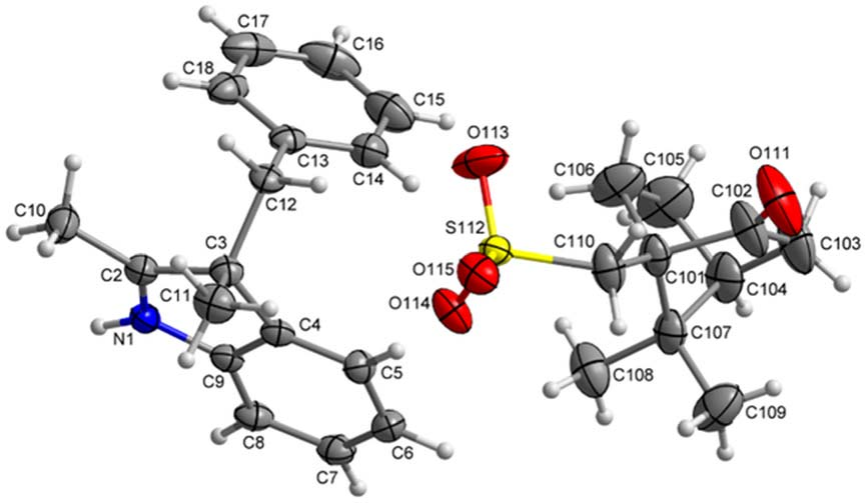
X-ray crystallographic structure of the optically pure salt of 3*H*-indole 5a.

The recrystallized optically pure salt of 3*H*-indole 5a and (–)-camphor-10-sulfonic acid was studied using X-ray crystallography and the absolute configuration of the isolated 3*H*-indole was assigned as (3-*R*) (Fig. 3). Details of the X-ray crystallography measurements are enclosed in the ESI.† The optically pure 3*H*-indole 5a was subsequently transformed into the corresponding spiropyran Ia using the synthetic protocol for the Series I. The reaction yields and spectroscopic data are in agreement with those of the spiropyran Ia using racemic 3*H*-indole 5a. To the best of our knowledge, we are the first to report a working protocol for synthesis and characterization of a spiropyran photoswitch with a defined stereochemistry in the position 3′ using purely chemical methods. Despite the modest yield, we believe the process can serve as a basis for further progress in search for optically pure spiropyrans.

#### Acid-mediated isolation and purification of the target spiropyrans

**Scheme 3:**
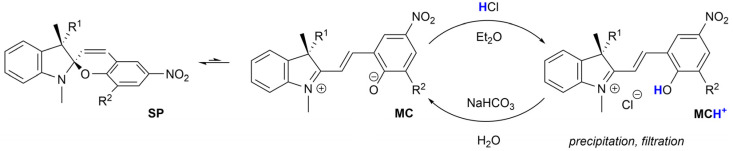
Acid-mediated procedure for purification of spiropyrans.

One of the most tedious processes in spiropyran synthesis is the final purification of the crude spiropyran reaction mixture. Common procedures involve liquid–liquid extraction followed by column chromatography using either silica gel stationary phase and highly polar mobile phases (*e.g.*, EtOAc/MeOH mixtures) or neutral/basic alumina with less polar mobile phases (hexane/dichloromethane/MeOH). The presented purification method is plain simple and straightforward. It utilizes acid-base-driven equilibrium between spiropyran (SP), merocyanine (MC), and protonated merocyanine (MCH^+^) forms (Scheme 3). First, the crude product is partitioned between Et_2_O and water to remove ammonium salts formed during the reaction. The organic layer is dried and acidified by a slow dropwise addition of 1.0 M ethereal HCl while being stirred. Full consumption of the spiropyran product is indicated by massive precipitation of yellow solid as the remaining liquid solution turns from dark blue/purple to transparent yellow. The formed solid is filtered and washed with Et_2_O. The isolated MCH^+^ is dissolved in dichloromethane and washed with saturated solution of NaHCO_3_. The washing process is accompanied by colour change in the organic layer from yellow to dark blue/purple as the MCH^+^ form switches back to SP/MC. The organic layer is dried, and the solvent evaporated to give a pure spiropyran.

This method of purification was successfully applied to the synthesis of the Series III and provided moderate to excellent yields (56–96%; for details see ESI†). The undisputable advantage of this process is low solvent consumption as well as overall cost as opposed to chromatographic methods, thus being potentially attractive for large-scale synthesis. This protocol serves as a convenient and practical methodology for purification of spiropyrans and can be widely used alongside traditionally utilized procedures.

### Diastereomeric preference of the target spiropyrans

**Fig. 4:**
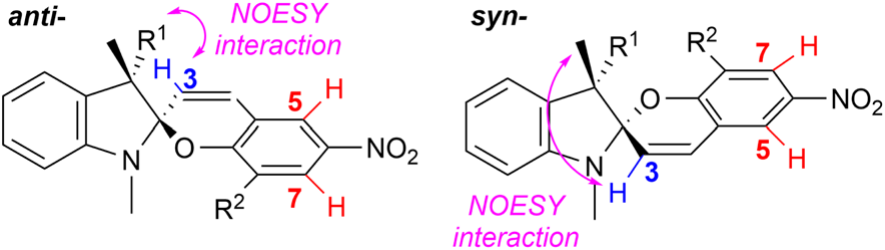
Signal assignment of the *syn*- and *anti*-epimers based on NMR NOESY experiments; the *syn* : *anti* ratios were calculated using signals of the H-3 protons or H-5/H-7 protons from ^1^H NMR spectra.

The diastereomeric preference of the target spiropyrans was determined by ^1^H NMR. The NMR samples were not subjected to photoisomerization prior to or during the measurement. The influence of five different solvents per each compound was studied using moderately polar chloroform as a benchmark solvent while two polar (acetone, DMSO) and two non-polar (toluene, benzene) solvents were tested as well. Each spectrum is a superposition of both relative stereoisomers (*syn*- and *anti*-) and, in some cases, also the open merocyanine form. The *syn* : *anti* ratio was estimated based on the integral values corresponding to the H-3 proton in the spectrum for both stereoisomers. For compounds IIIb–d, doublet signals H-5 and H-7 were utilized instead as there was an overlap of the H-3 signals with other proton multiplets. The *syn* : *anti* assignment of signals was performed using NMR NOESY experiments as demonstrated by Perry *et al.* (Fig. 4).^21^ The obtained results are summarized in Table 1.

**Table 1:** Diastereomeric ratios of the prepared spiropyrans

Entry	Compound	3′-Substitution	8-Substitution	CDCl_3_	Acetone-d_6_	DMSO-d_6_	Toluene-d_8_	Benzene-d_6_
1	Ia	PhCH_2_	H	25 : 75	31 : 69	26 : 74 (5)^a^	26 : 74	23 : 77
2	Ib	4-CH_3_-PhCH_2_	H	25 : 75	26 : 74	28 : 72 (8)^a^	22 : 78	26 : 74
3	Ic	4-OCH_3_-PhCH_2_	H	24 : 76	25 : 75	28 : 72 (4)^a^	21 : 79	22 : 78
4	Id	4-CF_3_-PhCH_2_	H	25 : 75	27 : 73	29 : 71 (4)^a^	21 : 79	23 : 77
5	Ie	3-CH_3_-PhCH_2_	H	27 : 73	30 : 70	35 : 65 (5)^a^	26 : 74	27 : 73
6	If	3-OCH_3_-PhCH_2_	H	25 : 75	28 : 72	30 : 70 (4)^a^	25 : 75	25 : 75
7	Ig	3-CF_3_-PhCH_2_	H	22 : 78	28 : 72	31 : 69 (4)^a^	26 : 74	22 : 78
8	IIa	Ph	H	51 : 49	49 : 51	50 : 50	50 : 50	50 : 50
9	IIb	4-CH_3_-Ph	H	52 : 48	46 : 54	45 : 55	52 : 48	53 : 47
10	IIc	4-OCH_3_-Ph	H	48 : 52	41 : 59	44 : 56	50 : 50	49 : 51
11	IId	4-CF_3_-Ph	H	49 : 51	40 : 60	36 : 64	49 : 51	51 : 49
12	IIe	4-NO_2_-Ph	H	49 : 51	38 : 62	41 : 59	55 : 45	55 : 45
13	IIIa	PhCH_2_	*t*Bu	27 : 73	28 : 72	31 : 69 (6)^a^	22 : 78	28 : 72
14	IIIb b	PhCH_2_	Ph	30 : 70	29 : 71	30 : 70 (21)^a^	28 : 72	30 : 70
15	IIIc b	PhCH_2_	4-F-Ph	29 : 71	29 : 71	29 : 71 (31)^a^	27 : 73	35 : 65
16	IIId b	PhCH_2_	4-*t*Bu-Ph	30 : 70	29 : 71	28 : 72 (23)^a^	28 : 72	28 : 72
17	IIIe c	PhCH_2_	1-Naphthyl	n.d.^d^	n.d^d^	n.d.^d^	30 : 70	31 : 69
18	IIIf	PhCH_2_	2-Naphthyl	28 : 72	31 : 69	28 : 72 (33)^a^	27 : 73	27 : 73

a Percentage of merocyanine with respect to the content of the whole sample (spiropyran + merocyanine form) is shown in the brackets.

b Syn : anti ratios quantified using H-5 and H-7 proton signals.

c Total four relative stereoisomers found due to axial chirality of the 1,1′-binaphthalene-like structure (see Fig. 5).

d Syn : anti ratio not determined due to a signal overlap.

The compounds of the Series I and III exhibited varying concentration of merocyanine in DMSO (4–33%). All other combinations of compounds and solvents showed either no merocyanine peaks at all or only small hints of these signals that could not be reliably quantified. Therefore, we estimate that the concentration of merocyanine in these samples was 0–2%. This observation is in agreement with the expected trend that highly polar solvents promote the formation of merocyanine. Surprisingly, no compound of the Series II exhibited notable amount of merocyanine regardless the solvent. This indicates that the materials IIa–e are not prone to the spiropyran opening process. This supposition is further supported by the obtained photokinetic data (see below). In the case of the Series III, notable increase of the merocyanine form was observed with respect to the analogous compound Ia with no substitution in the position 8. While the *tert*-butyl-substituted compound IIIa yielded comparable amount of merocyanine to Ia, the aryl-substituted spiropyrans IIIb–f reached up to 33% of merocyanine in DMSO. The amount of merocyanine for IIIe was not determined due to signal overlap. However, the spectrum clearly indicated substantial concentration of the merocyanine form. These results indicate that the presence of an aryl moiety in the position 8 supports the formation of merocyanine in strongly polar media, such as DMSO. This effect is likely not a result of steric hindrance as no such increase in the merocyanine concentration was found for IIIa.

#### Series I

The compounds of the Series I show a correlation between the *syn* : *anti* ratio and solvent polarity. Chloroform solutions exhibit *syn* : *anti* = 25 : 75 on average for all the compounds Ia–g. More polar solvents (acetone, DMSO) decrease the *anti*-epimer prevalence up to 35 : 65 (Ie; Table 1, entry 5). DMSO (the most polar solvent used) provides the lowest *syn* : *anti* ratio except for Ia (Table 1, entry 1) where the lowest *anti*-abundance was found in acetone. On the other hand, low-polar solvents (benzene, toluene) yield the highest *anti*-preference (*syn* : *anti* up to 21 : 79). We also tested cyclohexane-*d*_12_ as a non-polar and non-polarizable solvent. However, the prepared spiropyrans were insoluble thereof. Despite low *syn* : *anti* differences across the whole Series I, the observed trend shows steady increase of the diastereomeric ratio with decreasing solvent polarity.

Within the Series I, two structural patterns were studied: 4-substituted, and 3-substituted benzylic units in the position 3′ of the spiropyran scaffold (for examples of 2-substituted analogues see ref. 21). In polar environment (DMSO, acetone), slight preference of the *anti*-epimer was observed for the 4-substituted phenyl derivatives (Ib–d; Table 1, entries 2–4) over the 3-substituted materials (Ie–g; Table 1, entries 5–7). The same trend is prevalent in toluene as well. In chloroform and benzene, only minor differences were found. Overall, substitution in the position 4 of the benzylic arm yields comparable or slightly higher *anti*-preference than the non-substituted compound Ia. On the other hand, substitution in the position 3 generally delivers similar or slightly lower *anti*-abundance than Ia.

The electronic effects of the substituents used (CH_3_, OCH_3_, CF_3_) appear to have very limited influence on the stereochemical preference. The strongly electron withdrawing CF_3_ group and electron donating OCH_3_ group have a slightly positive effect on the *syn* : *anti* ratio with respect to the CH_3_ substituted compounds (Ib, Ie). This effect is mostly prevalent in benzene and, for the 3-substituted compounds Ie–g also in chloroform, acetone, and DMSO. For the remaining combinations of compounds and solvents, the electronic effect is mostly negligible.

#### Series II

Based on the results for the Series II, the substitution pattern used has little to no influence on the stereochemical bias of the target spiropyrans (Table 1, entries 8–12). If the aromatic ring is oriented closer to the indoline unit (compare Series I and II), we speculate that it does not reach far enough to exert any notable influence on the chromene moiety, thus enforcing only very small epimeric abundance. The compound IIa with an unsubstituted phenyl group in the position 3′, shows no *syn* : *anti* preference regardless the type of solvent (Table 1, entry 8). Other compounds (IIb–e), that hold a substituent in the position 4 of the aromatic side arm, exhibit minor bias towards the *anti*-epimer in polar solvents (acetone, DMSO). In chloroform, benzene, and toluene, only subtle differences from equal *syn* : *anti* ratios were found. Analogously to the Series I, strong electron withdrawing and, to minor extent, electron donating effects (R = CF_3_, NO_2_, OCH_3_) provide higher diastereomeric preference over weaker or no electronic effects (R = H, CH_3_). This factor implies a broader trend that substituents exerting strong polarization have partial positive influence on the energy differentiation of the spiropyran epimers thus increasing their diastereomeric ratio.

Despite low effect of less polar solvents (chloroform, toluene, benzene), the NO_2_-substituted compound IIe yields minor *syn*-preference in toluene and benzene (*syn* : *anti* = 55 : 45). Lesser, but still notable, *syn*-bias was found for the methyl-substituted compound IIb. Despite the low diastereomeric excesses found for the Series II, it is worth noting that a change of solvent can lead to opposite epimeric preference. This highlights the significance of solvent selection while contemplating potential applications.

#### Series III

The Series III features a benzyl group in the position 3′ to generate a secondary chiral centre while exploring the influence of substitution in the position 8 of the chromene subunit. Spiropyran IIIa bears a *tert*-butyl moiety, while other compounds of this series (IIIb–f) are substituted with an additional aromatic ring in this position.

Compound IIIa exhibits by far the largest span of *syn* : *anti* values throughout the series ranging from 31 : 69 in DMSO to 22 : 78 in toluene (Table 1, entry 13). This finding further supports the trend found in the Series I, reflecting the influence of the solvent polarity on the stereochemical preference. The sole effect of the *tert*-butyl group in the position 8, however, brings no conclusive improvement over the unsubstituted compound Ia. In acetone and toluene, IIIa yields higher *anti*-preference than Ia, whereas in chloroform, DMSO, and benzene, the opposite is true. Compounds IIIb–f each provide almost identical diastereomeric preference (*syn* : *anti* = approx. 30 : 70) no matter the solvent used (Table 1, entries 14–18). The only exception from this trend is the 4-fluorophenyl-substituted compound IIIc in benzene where *syn* : *anti* = 35 : 65 ratio was found. The unravelled trend suggests that the auxiliary aromatic ring in the position 8 stabilizes the *syn* : *anti* ratio regardless the solvent used. The effect of the substitution, however, does not improve the results found for the compound Ia and is, in fact, slightly detrimental (*syn* : *anti* max. up to 27 : 73). The two functionalities in the position 3′ and 8, respectively, are likely too far away from each other to cause a notable steric hindrance in the *syn*-isomer. Thus, no major improvement over the compound Ia was found.

The compound IIIe bears a 1-naphthyl group in the position 8 of the chromene unit. Therefore, it acts as a 1,1′-binaphthalene system that appears in two rotameric forms. The spiropyran IIIe thus holds three elements of chirality instead of two as the other compounds. As a result, eight diastereomers, *i.e.*, four relative stereoisomers, are formed. The relative ratio of these stereoisomers could not be elucidated using 400 MHz NMR in chloroform, acetone, and DMSO due to overlapping signals. Thus, only data for toluene and benzene are shown. If the effect of axial chirality is neglected, the *syn* : *anti* ratio equals to 30 : 70 for toluene, and 31 : 69 for benzene, respectively (Table 1, entry 17). The actual ratio of the four relative stereoisomers is, however, 13 : 17 : 43 : 27 for toluene, and 12 : 19 : 39 : 30 for benzene, respectively. The signal assignment for the *syn*- and *anti*-stereoisomers was carried out using NMR NOESY (Fig. 5).

**Fig. 5:**
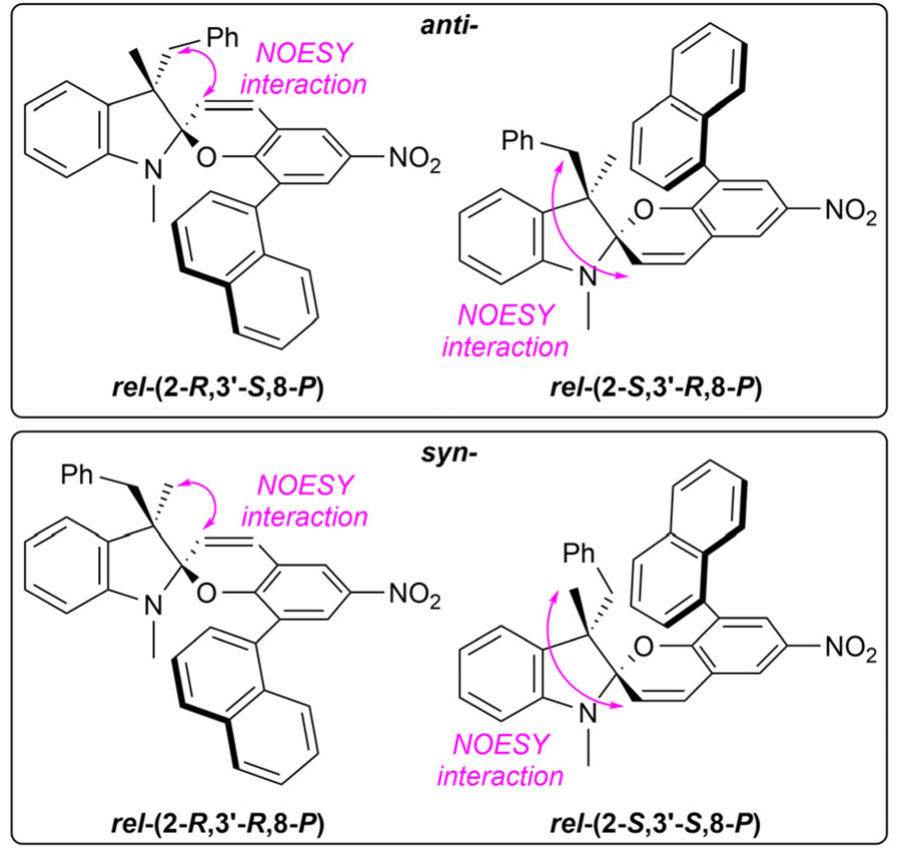
Signal assignment of the IIIe relative stereoisomers based on NMR NOESY experiment.

### Photokinetic studies

**Fig. 6:**
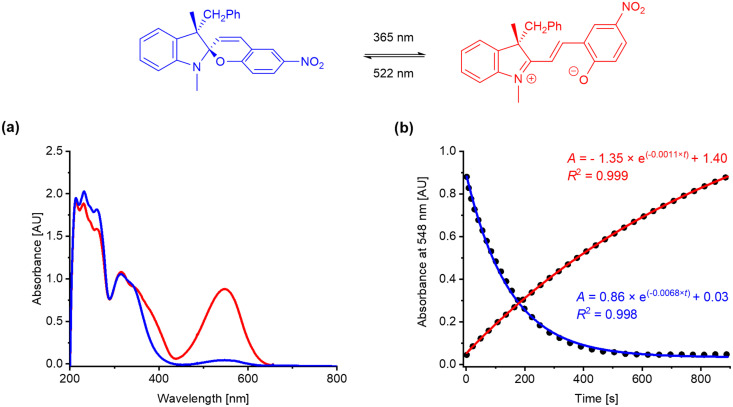
(a) Absorption profile of photostationary states of Ia after irradiation with green light (blue curve) and UV light (red curve); (b) kinetic plot of spiropyran opening (red curve) and closing (blue curve) for Ia at 548 nm.

The photokinetic properties of the target spiropyrans were studied using UV-vis spectroscopy of methanol solutions at concentration 10^−4^ mol L^−1^ in quartz glass fluorescent cuvettes (path length 1 cm, *λ* = 200–800 nm). The photoswitching processes were triggered by a UV diode (*λ* = 365 nm, 175 mW) for spiropyran opening and a green diode (*λ* = 522 nm, 60 mW) for spiropyran closing, respectively. The progress of both the opening (*k*_SP-MC_) and closing (*k*_MC-SP_) transition was monitored at wavelength corresponding to the absorption maximum of the merocyanine band (*λ*_max_*ca.* 540–580 nm). The rate constants *k*_SP-MC_ and *k*_MC-SP_ were calculated based on the assumption of first order kinetics. The results are summarized in Table 2. The values *A*_max_ correspond to the maximal absorbance found at *λ*_max_ during the photoswitching measurements, *i.e.*, in the photostationary state upon UV light irradiation (at maximal merocyanine concentration). UV-vis spectra and kinetic plots for Ia are shown in Fig. 6. Experimental details, UV-vis spectra and kinetic plots for the remaining spiropyrans are available in the ESI.† To the best of our knowledge, we are the first to report any data on photoswitching of optically biased spiropyrans with an auxiliary chiral centre in the position 3′.

**Table 2:** Rates of the light-induced photoswitching of the target spiropyrans (MeOH, *c* = 10^−4^ mol L^−1^)

Entry	Compound	3′-Substitution	8-Substitution	*λ*_max_ (nm)	*A*_max_ (AU)	*k*_SP-MC_ (10^−3^ s^−1^)	*k*_MC-SP_ (10^−3^ s^−1^)
1	Ia	PhCH_2_	H	548	0.88	1.1	6.8
2	Ib	4-CH_3_-PhCH_2_	H	548	0.56	2.5	3.2
3	Ic	4-OCH_3_-PhCH_2_	H	548	0.77	2.2	3.0
4	Id	4-CF_3_-PhCH_2_	H	548	0.79	3.0	3.0
5	Ie	3-CH_3_-PhCH_2_	H	547	0.62	2.5	3.2
6	If	3-OCH_3_-PhCH_2_	H	548	0.78	2.6	2.7
7	Ig	3-CF_3_-PhCH_2_	H	558	0.44	5.6	5.7
8	IIa	Ph	H	549	0.36	8.1	5.6
9	IIb	4-CH_3_-Ph	H	544	0.17	7.6	8.8
10	IIc	4-OCH_3_-Ph	H	550	0.06	9.3	10.4
11	IId	4-CF_3_-Ph	H	552	0.10	30.8	27.1
12	IIe	4-NO_2_-Ph	H	559	0.05	15.4	13.6
13	IIIa	PhCH_2_	*t*Bu	578	2.76	146.2	22.1
14	IIIb	PhCH_2_	Ph	577	2.20	56.1	11.4
15	IIIc	PhCH_2_	4-F-Ph	577	2.33	37.9	11.9
16	IIId	PhCH_2_	4-*t*Bu-Ph	570	2.95	64.1	4.9
17	IIIe	PhCH_2_	1-Naphthyl	573	1.00	63.4	60.9
18	IIIf	PhCH_2_	2-Naphthyl	573	1.70	35.3	8.0

The compounds of the Series I exhibit the slowest photoswitching overall, not exceeding 10^−3^ s^−1^ rate. The non-substituted material Ia has a notable discrepancy between the kinetic rate of spiropyran opening and closing (Table 2, entry 1). This suggests that Ia prefers to adopt its closed spiropyran form even in polar solvents, such as methanol, where the merocyanine form is more stabilized in contrast to less polar media. In comparison to Ia, the compounds Ib–g exhibit faster spiropyran opening and slower closing. Similar kinetic rates for both opening and closing processes were found with the ring closing being only slightly faster (Table 2, entries 2–7). The compound Ig with the electron withdrawing CF_3_ group in the position 3 of the benzylic side arm provides double kinetic rates for both processes over the other substituted compounds from the Series I (Ib–f). The analogous compound Id with a CF_3_ group in the position 4 of the benzyl moiety exhibits minor increase in spiropyran opening rate over other compounds bearing electron donating moieties (CH_3_, OCH_3_). This indicates that a presence of an electron withdrawing group generally supports the photoswitching rate.

The compounds of the Series I provide similar values of *A*_max_. The highest *A*_max_ = 0.88 was found for Ia, while the lowest *A*_max_ = 0.44 for Ig. Assuming the molar extinction coefficients of the observed absorption bands of Ia–g are comparable, the *A*_max_ values indicate that any substitution on the benzylic side arm in the position 3′ partly shifts the SP-MC photostationary state equilibrium towards the spiropyran form.

The Series II, bearing aromatic moieties in the position 3′ instead of benzylic groups, exhibits substantially faster photoswitching than the Series I. The kinetic rates of both processes (opening, closing) are similar for each compound with the rate of opening being slightly prevalent for IIa, IId, and IIe. For IIb and IIc, the opposite is true. While compounds IIa–c either non-substituted or substituted with an electron donating group show comparable kinetic rates (*ca.* 5–10 × 10^−3^ s^−1^), the CF_3_-substituted material IId exhibits tripled values for both opening and closing. This finding supports the trend of the influence of electron withdrawing moieties on the kinetic rates outlined for the Series I.

The *A*_max_ values of the compounds of the Series II are considerably lower than for the other series. This indicates that the spiropyrans IIa–e exhibit very little change upon external light stimulus. Therefore, direct aryl-substitution in the position 3′ stabilizes the spiropyran form to the extent that, at given conditions, it acts as a poor photoswitch.

All the compounds of the Series III show highly accelerated spiropyran opening kinetics in comparison to the unsubstituted compound Ia. The highest photoswitching rates of opening were found for IIIa with a *tert*-butyl group (Table 2, entry 13). The fastest switching for closing was found for the 1-naphthyl-substituted material IIIe (Table 2, entry 17). In the case of other materials (IIIb–d, IIIf), dramatic difference between the opening and closing rates were observed similarly to IIIa. While the rates of closing are comparable to the non-substituted material Ia, the rates of opening are increased by an order of magnitude. In general, based on the variability of the substituents in the Series III, we assume that any bulky group in the position 8 of the chromene unit promotes spiropyran opening.

All the compounds from the Series III show higher *λ*_max_ than those from the two other series including the *tert*-butyl-substituted material IIIa. Therefore, the observed band shift is not a result of the aromatic character of the substituents in the position 8 (IIIb–f), but rather a general auxochromic effect of these functionalities. Throughout the whole series, notably high values of *A*_max_ were found in contrast to the other series. Therefore, it is safe to assume that the compounds of this series provide the highest merocyanine concentrations upon irradiation. This is especially true for the compounds IIIa–d, where *A*_max_ ≥ 2.2 AU. In the case of the naphthyl-substituted compounds IIIe–f, the *A*_max_ is partially decreased, however, still surpasses the values for the compounds of the Series I and II. Overall, the collected data for the Series III shows that the substitution in the position 8 of the spiropyran scaffold supports both fast photoswitching and high concentration changes of the spiropyran and merocyanine form. Such feature is a necessary precondition for potential applications, *e.g.*, in materials chemistry of low-molecular sensors and light-tunable liquid crystals.^12^

## Conclusions

Three series of spiropyran photoswitches (18 compounds) with an additional element of chirality in the position 3′ of the indoline subunit were synthesized. The target materials were yielded in racemic form with no stereocontrol over the chiral centre in the position 3′. One compound (Ia), however, was also prepared with a defined chirality in this position using co-crystallization of its 3*H*-indole precursor with optically pure (–)-camphor-10-sulfonic acid. The absolute configuration of this intermediate was determined using X-ray crystallography. To the best of our knowledge, this is an unprecedented method for synthesis of optically active spiropyrans thus far. Moreover, a novel approach to spiropyran purification using its acid–base equilibrium was developed. This method represents a complementary protocol to conventional techniques (*e.g.*, column chromatography) for purification of such compounds and can serve as a cheap alternative for scale-up synthesis.

The stereochemical bias of the prepared compounds in various solvents (chloroform, acetone, DMSO, toluene, benzene) was evaluated using ^1^H NMR spectroscopy at 25 °C. The assignment of the NMR signals to the corresponding relative stereoisomers (*syn*-, *anti*-) was performed using NOESY. Series I exhibited *syn* : *anti* ratios up to 21 : 79 with notable influence of the solvent used. In general, non-polar solvents were found to yield the highest stereochemical preference. The compounds of the Series II showed little to no stereochemical bias. In the case of the compound IIe, rather uncommon (minor) *syn*-preference was observed. All photoswitches throughout the Series III (excluding IIIa) afforded comparable *syn* : *anti* ratio (*ca.* 30 : 70) regardless of the substitution pattern or the solvent applied. The compounds of the series I and III also exhibited varying amounts of the merocyanine form in DMSO, which were notably increased for IIIb–f. It was thereby postulated that a presence of an aromatic unit in the position 8 promotes the spiropyran opening in polar solvents.

The photochemical switching of the target compounds was monitored by UV-vis spectroscopy. Throughout Series I and II, a presence of an electron withdrawing group in the aromatic side arm in the position 3′ accelerated the photoswitching kinetics by a factor of up to 5 with respect to the non-substituted compounds Ia and IIa, respectively. The main difference between the photokinetics of the Series I and II is very low maximal abundance of the merocyanine form for IIa–e. The substitution pattern of the Series II (direct aryl substitution in the position 3′) thus appears to stabilize the spiropyran form considerably. Compounds of the Series III exhibited one to two orders of magnitude higher kinetic rates of spiropyran opening than the unsubstituted benchmark compound Ia. For IIIa and IIIe, the same was true for the kinetics of spiropyran closing, whereas IIIb–d and IIIf showed similar closing rate constants as Ia. The Series III also provided the highest concentration of merocyanine upon irradiation with UV light, especially in the case of the compounds IIIa–d. This indicates that substitution of the spiropyran scaffold in the position 8 has a dramatic effect on both the kinetics and thermodynamics of the photoswitching process.

## Data availability

Detailed synthesis and characterization of the prepared compounds, X-ray crystallographic data, NMR spectra, and photokinetic plots are included in ESI.† The X-ray structure was deposited into the Cambridge Structural Database under number CCDC 2393565.†

## Author contributions

Vojtěch Boháček, Tereza Erbenová, Jakub Dávid Malina and Marie Kloubcová – performed the experiments, characterized the compounds and interpreted the data. Václav Eigner – performed the X-ray crystallography experiment and interpreted the data. Michal Šmahel – supervised the project, interpreted the data and edited the manuscript. Jiří Tůma – designed and supervised the project, wrote the manuscript and provided funding.

## Conflicts of interest

There are no conflicts to declare.

## Supplementary Material

RA-014-D4RA07750D-s001

RA-014-D4RA07750D-s002
