# Supplementary material for: Spectroscopic and photochemical evaluation of stereochemically biased 3′-substituted spiropyran photoswitches
Source: RSC Adv. 2024 Nov 21;14(50):37370–9. doi: 10.1039/d4ra07750d (PMC11580008; doi:10.1039/d4ra07750d)
Supplement: RA-014-D4RA07750D-s001 [file RA-014-D4RA07750D-s001.pdf]

Supplementary Information (SI) for RSC Advances.
This journal is © The Royal Society of Chemistry 2024

Electronic Supplementary Material

Towards optically pure spiropyrans: spectroscopic and photochemical evaluation of stereochemically biased spiropyran photoswitches

Vojtěch Boháček[1], Tereza Erbenová[1], Jakub Dávid Malina[1], Marie Kloubcová[1], Michal Šmahel[1], Václav Eigner[2] and Jiří Tůma[1*]

[1]Department of Organic Chemistry, University of Chemistry and Technology Prague, Technická 5, Prague 6, 166 28, Prague, Czech Republic

[2]Department of Solid State Chemistry, University of Chemistry and Technology Prague, Technická 5, Prague 6, 166 28, Prague, Czech Republic

email: tumaa@vscht.cz

Table of contents

1. Instruments, materials and methods **2**

2. Synthesis **3**

2.1 Synthesis of the Series I **3**
2.2 Synthesis of the Series II **14**
2.3 Synthesis of the Series III **22**
2.4 Isolation of optically pure 3*H*-indole 5a **28**

3. X-Ray crystallography of 5a_salt **29**

4. ^{1}H spectra of 2,3-dimethyl-3-benzyl-3*H*-indolium (–)-camphor-10-sulfonate **31**

5. ^{1}H and ^{13}C NMR spectra of the target spiropyrans **32**

6. Photokinetic plots **50**

7. References **56**

1. Instruments, materials and methods

NMR

The NMR spectra were measured on Agilent 400-MR DDR2 and JEOL JNMECZL400G with working frequency 400 MHz for ^{1}H, 101 MHz for ^{13}C and 376 MHz for ^{19}F spectra. The samples were dissolved in deuterated solvents and referenced internally to the residual non-deuterated solvent. Chemical shifts δ are reported in ppm units and spin-spin coupling constants J in Hz. The multiplicities of signals are characterized by following symbols: s (singlet), d (doublet), dd (doublet of doublets), ddd (doublet of doublets of doublets), t (triplet), dt (doublet of triplets), td (triplet of doublets), q (quartet), m (multiplet).

HRMS

High resolution mass spectra were recorded on LTQ Orbitrap Velos (Thermo Fischer Scientific) under ESI or APCI ionization.

UV-VIS and photokinetic measurements

Absorption spectra and photokinetic plots were measured in quartz cuvettes with path length 10 mm on Agilent Cary 8454 UV-VIS spectrophotometer. The analytes were dissolved in MeOH (HPLC grade; $c = 10^{-4}$ mol/L). The photokinetic processes were triggered by photodiodes ($\lambda_1 = 365$ nm, $p = 175$ mW; $\lambda_2 = 522$ nm, $p = 60$ mW).

Chromatography

TLC analyses were performed on aluminium TLC plates coated with silica gel 60 F_{254} (Merck). Purification by column chromatography was carried out using silica gel Kieselgel 60 (Merck) with granularity of 63–100 μm. Spiropyrans of the Series I and II were purified by column chromatography on neutral aluminium oxide (Sigma-Aldrich; Brockmann I type).

Reagents and solvents

Solvents were distilled prior to use. Dry solvents were dried using solvent purification system PURESOLV MD7 and stored over sodium pellets or molecular sieves (4Å).

Unless stated otherwise, all reagents were commercially available products and were used without further purification.

2. Synthesis

2.1 Synthesis of the Series I

*<u>Unsaturated ketones **3a-g**</u>*

*General protocol **A***

96% H_2SO_4 (1.00 eq.) was slowly added dropwise to a mixture of arene carbaldehyde **2** (1.00 eq.) and butanone (2.00 eq.) in AcOH (c = 5 mol/L). The reaction mixture was stirred at room temperature overnight. The reaction was alkalised with 10% aq. NaOH to pH = 7 and extracted with ethyl acetate (3 × 50 mL). Organic phase was washed with saturated aq. solution of $NaHCO_3$ (2 × 50 mL), brine (50 mL) and then dried over $MgSO_4$. Solvents were evaporated under reduced pressure. The crude product was purified by column chromatography using hexane:ethyl acetate, 8:1 to 6:1 mixture as eluent.

*4-Phenyl-3-methylbut-3-en-2-one (**3a**)*

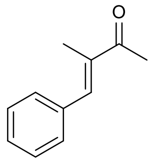

The title compound was prepared according to the general protocol **A** using 14.6 mmol (0.81 mL) of H_2SO_4, 14.6 mmol (1.5 g) of benzaldehyde, and 29.2 mmol (2.11 g) of butanone. The product was yielded as a yellow oil (1.93 g; 81%).

^{1}H NMR (400 MHz, $CDCl_3$) δ 7.52 (q, J = 1.6 Hz, 1H), 7.44 – 7.32 (m, 5H), 2.47 (s, 3H), 2.06 (d, J = 1.6 Hz, 3H).

^{13}C NMR (400 MHz, $CDCl_3$) δ 200.3, 139.7, 137.8, 135.9, 129.7, 128.6, 128.5, 25.9, 12.9.

The NMR data is in agreement with ref. [S1].

*4-(4-Methylphenyl)-3-methylbut-3-en-2-one (**3b**)*

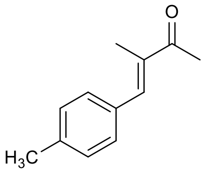

The title compound was prepared according to the general protocol **A** using 25.0 mmol (1.39 mL) of H_2SO_4, 25.0 mmol (3.00 g) of 4-methylbenzaldehyde, and 50.0 mmol (3.61 g) of butanone. The product was yielded as a yellow oil (1.88 g; 44%).

^{1}H NMR (400 MHz, $CDCl_3$) δ 7.48 (s, 1H), 7.32 (d, J = 8.2 Hz, 2H), 7.20 (d, J = 8.0 Hz, 2H), 2.43 (s, 3H), 2.36 (s, 3H), 2.04 (s, 3H).

^{13}C NMR (101 MHz, $CDCl_3$): δ 200.0, 140.3, 139.1, 137.2, 133.2, 130.2, 129.6, 26.3, 22.0, 13.4.

The NMR data is in agreement with ref. [S1].

*4-(4-Methoxyphenyl)-3-methylbut-3-en-2-one (**3c**)*

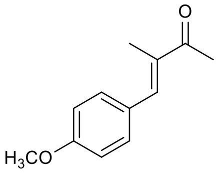

The title compound was prepared according to the general protocol **A** using 12.5 mmol (0.69 mL) of H_2SO_4, 12.5 mmol (1.70 g) of 4-methoxybenzaldehyde, and 25.0 mmol (1.80 g) of butanone. The product was yielded as a yellow oil (2.10 g; 87%).

^{1}H NMR (400 MHz, $CDCl_3$) δ 7.47 (s, 1H), 7.41 (d, J = 8.8 Hz, 2H), 6.94 (d, J = 8.8 Hz, 2H), 3.84 (s, 3H), 2.44 (s, 3H), 2.06 (s, 3H).

^{13}C NMR (101 MHz, $CDCl_3$): δ 200.4, 160.1, 139.7, 136.0, 131.7, 128.6, 114.1, 55.5, 25.9, 13.1.

The NMR data is in agreement with ref. [S1].

*4-(4-Trifluorophenyl)-3-methylbut-3-en-2-one (**3d**)*

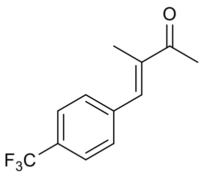

The title compound was prepared according to the general protocol **A** using 17.0 mmol (0.94 mL) of H_2SO_4, 17.0 mmol (2.96 g) of 4-trifluoromethylbenzaldehyde, and 34.0 mmol (2.45 g) of butanone. The product was yielded as a yellow oil (1.65 g; 43%).

^{1}H NMR (400 MHz, $CDCl_3$): δ 7.67 (d, J = 8.8 Hz, 2H), 7.51 (d, J = 8.8 Hz, 2H), 7.50 (q, J = 1.4 Hz, 1H), 2.48 (s, 3H), 2.04 (d, J = 1.4 Hz, 3H).

^{13}C NMR [^{19}F] (101 MHz, $CDCl_3$): δ 200.0, 139.7, 139.6, 137.7, 130.4, 129.9, 125.5, 124.7, 26.1, 13.2.

^{19}F NMR (376 MHz, $CDCl_3$): δ –62.7.

The NMR data is in agreement with ref. [S2].

*4-(3-Methylphenyl)-3-methylbut-3-en-2-one (**3e**)*

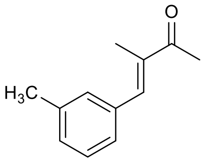

The title compound was prepared according to the general protocol **A** using 15.0 mmol (0.83 mL) of H_2SO_4, 15.0 mmol (1.80 g) of 3-methylbenzaldehyde, and 30.0 mmol (2.16 g) of butanone. The product was yielded as a yellow oil (1.08 g; 42%).

^{1}H NMR (400 MHz, $CDCl_3$): δ 7.49 (s, 1H), 7.35 – 7.26 (m, 1H), 7.24 – 7.19 (m, 2H), 7.15 (d, J = 7.9 Hz, 1H), 2.45 (s, 3H), 2.38 (d, J = 0.7 Hz, 3H), 2.04 (d, J = 1.4 Hz, 3H).

^{13}C NMR (101 MHz, $CDCl_3$): δ 200.5, 140.0, 138.2, 137.7, 136.0, 130.5, 129.5, 128.5, 126.9, 26.0, 21.5, 13.1.

The NMR data is in agreement with ref. [S3].

*4-(3-Methoxyphenyl)-3-methylbut-3-en-2-one (**3f**)*

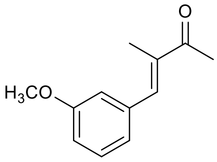

The title compound was prepared according to the general protocol **A** using 15.0 mmol (0.83 mL) of H_2SO_4, 15.0 mmol (2.04 g) of 3-methoxybenzaldehyde, and 30.0 mmol (2.16 g) of butanone. The product was yielded as a yellow oil (1.10 g; 48%).

^{1}H NMR (400 MHz, $CDCl_3$): δ 7.48 (s, 1H), 7.36 – 7.28 (m, 1H), 7.00 (ddt, J = 7.6, 1.6, 0.8 Hz, 1H), 6.93 (t, J = 2.1 Hz, 1H), 6.88 (ddd, J = 8.2, 2.6, 0.9 Hz, 1H), 3.82 (s, 3H), 2.45 (s, 3H), 2.04 (d, J = 1.4 Hz, 3H).

^{13}C NMR (101 MHz, $CDCl_3$): δ 200.5, 159.6, 139.7, 138.1, 137.3, 129.6, 122.3, 115.2, 114.2, 55.4, 26.0, 13.1.

The NMR data is in agreement with ref. [S1].

*4-(3-Trifluoromethylphenyl)-3-methylbut-3-en-2-one (**3g**)*

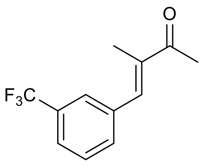

The title compound was prepared according to the general protocol **A** using 12.5 mmol (0.69 mL) of H_2SO_4, 12.5 mmol (2.18 g) of trifluoromethylbenzaldehyde, and 25.0 mmol (1.80 g) of butanone. The product was yielded as a yellow oil (1.71 g; 50%).

^{1}H NMR (400 MHz, $CDCl_3$): δ 7.67 – 7.60 (m, 1H), 7.61 – 7.49 (m, 4H), 2.46 (s, 3H), 2.03 (d, J = 1.4 Hz, 3H).

^{13}C NMR [^{19}F] (101 MHz, $CDCl_3$): *δ* 200.0, 139.3, 137.8, 136.8, 132.7, 131.1, 129.1, 126.4, 125.2, 124.0, 26.0, 13.0.

^{19}F NMR (376 MHz, $CDCl_3$): *δ* –62.7 (s, 3F).

*Methyl ketones **4a-g***

*General protocol **B***

10% (w/w) Pd/C (1.5 – 2.1 mol%) was added in a single portion to a solution of α,β-unsaturated ketone **3** (1.00 eq.) in dry MeOH (c = 0.2 mol/L). The reaction vessel was repeatedly flushed with H_2 atmosphere (3 ×), heated to 60 °C and vigorously stirred for 4 h. The reaction mixture was cooled down to ambient temperature and the solid catalyst was filtered off. The solvent was removed under reduced pressure and the crude product purified by column chromatography using hexane:ethyl acetate, 1:1 mixture as eluent.

*4-Phenyl-3-methylbutan-2-one (**4a**)*

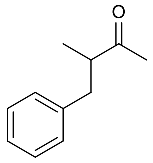

The title compound was prepared according to the general protocol **B** using 10 mmol (1.60 g) of ketone **3a** and 0.15 mmol (0.16 g) of 10% (w/w) Pd/C. The product was yielded as a yellow oil (1.20 g; 74%).

^{1}H NMR ($CDCl_3$, 400 MHz): *δ* 7.30 – 7.25 (m, 2H), 7.22 – 7.12 (m, 3H), 3.00 (dd, *J* = 13. 5, 6.8 Hz, 1H), 2.88 – 2.79 (m, 1H), 2.56 (dd, *J* = 13.5, 6.7 Hz), 2.09 (s, 3H), 1.09 (d, *J* = 6.9 Hz)

^{13}C NMR ($CDCl_3$, 101 MHz): *δ* 212.2, 140.4, 128.9, 128.4, 122.4, 48.8, 38.9, 28.9, 16.2.

The NMR data is in agreement with ref. [S1].

*4-(4-Methylphenyl)-3-methylbutan-2-one (**4b**)*

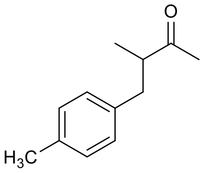

The title compound was prepared according to the general protocol **B** using 11 mmol (1.92 g) of ketone **3b** and 0.18 mmol (0.19 g) of 10% (w/w) Pd/C. The product was yielded as a yellow oil (1.35 g; 72%).

^{1}H NMR ($CDCl_3$, 400 MHz): *δ* 7.10 (d, *J* = 8.2 Hz ,2H), 7.05 (d, *J* = 8.2 Hz, 2H), 2.96 (dd, *J* = 13.6, 6.8 Hz, 1H), 2.81 (m, *J* = 7.0 Hz, 1H), 2.53 (dd, *J* = 13.5, 7.7 Hz, 1H), 2.32 (s, 3H), 2.09 (s, 3H), 1.09 (d, *J* = 7.0 Hz, 3H).

^{13}C NMR ($CDCl_3$, 101 MHz): *δ* 212.1, 136.5, 135.7, 129.1, 128.8, 48.9, 38.5, 28.8, 21.0, 16.2.

The NMR data is in agreement with ref. [S1].

*4-(4-Methoxyphenyl)-3-methylbutan-2-one (**4c**)*

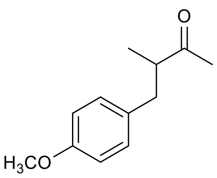

The title compound was prepared according to the general protocol **B** using 5.6 mmol (1.07 g) of ketone **3c** and 0.09 mmol (0.10 g) of 10% (w/w) Pd/C. The product was yielded as a yellow oil (0.75 g; 70%).

^{1}H NMR (400 MHz, $CDCl_3$): *δ* 7.09 – 7.04 (m, 2H), 6.84 – 6.79 (m, 2H), 3.78 (s, 3H), 2.93 (dd, *J* = 13.6, 6.9 Hz, 1H), 2.79 (m, 1H), 2.51 (dd, *J* = 13.6, 7.6 Hz, 1H), 2.08 (s, 3H), 1.08 (d, *J* = 6.9 Hz, 3H).

^{13}C NMR (101 MHz, $CDCl_3$): *δ* 212.5, 158.2, 131.8, 130.0, 114.0, 55.4, 49.2, 38.3, 29.1, 16.4.

The NMR data is in agreement with ref. [S1].

*4-(4-trifluoromethylphenyl)-3-methylbutan-2-one (**4d**)*

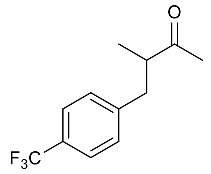

The title compound was prepared according to the general protocol **B** using 7.0 mmol (1.60 g) of ketone **3d** and 0.15 mmol (0.16 g) of 10% (w/w) Pd/C. The product was yielded as a yellow oil (1.42 g; 88%).

^{1}H NMR (400 MHz, $CDCl_3$): *δ* 7.55 – 7.51 (m, 2H), 7.29 – 7.25 (m, 2H), 3.07 (dd, *J* = 13.6, 7.0 Hz, 1H), 2.84 (m, 1H), 2.61 (dd, *J* = 13.6, 7.5 Hz, 1H), 2.11 (s, 3H), 1.12 (d, *J* = 7.0 Hz, 3H).

^{13}C NMR [^{19}F] (101 MHz, $CDCl_3$): *δ* 211.7, 144.0, 129.4, 128.7, 125.5, 123.0, 48.6, 38.5, 29.1, 16.6.

^{19}F NMR (376 MHz, $CDCl_3$): *δ* –62.3.

The NMR data is in agreement with ref. [S2].

*4-(3-Methylphenyl)-3-methylbutan-2-one (**4e**)*

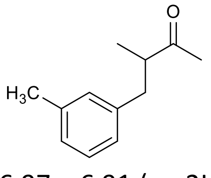

The title compound was prepared according to the general protocol **B** using 5.0 mmol (0.87 g) of ketone **3e** and 0.08 mmol (0.09 g) of 10% (w/w) Pd/C. The product was yielded as a yellow oil (0.44 g; 50%).

^{1}H NMR (400 MHz, $CDCl_3$): *δ* 7.16 (t, *J* = 7.6 Hz, 1H), 7.00 (d, *J* = 7.6 Hz, 1H), 6.97 – 6.91 (m, 2H), 2.95 (dd, *J* = 13.5, 6.7 Hz, 1H), 2.81 (m 1H), 2.51 (dd, *J* = 13.5, 7.9 Hz, 1H), 2.31 (s, 3H), 2.09 (s, 3H), 1.08 (d, *J* = 7.0 Hz, 3H).

^{13}C NMR (101 MHz, $CDCl_3$): *δ* 212.4, 139.7, 138.1, 129.8, 128.4, 127.1, 126.0, 48.9, 38.9, 29.0, 21.5, 16.3.

*4-(3-Methoxyphenyl)-3-methylbutan-2-one (**4f**)*

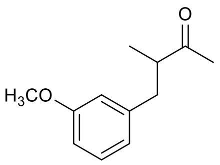

The title compound was prepared according to the general protocol **B** using 1.3 mmol (0.25 g) of ketone **3f** and 0.02 mmol (0.025 g) of 10% (w/w) Pd/C. The product was yielded as a yellow oil (0.20 g; 80%).

^{1}H NMR (400 MHz, $CDCl_3$): *δ* 7.18 (t, *J* = 8.1 Hz, 1H), 6.75 – 6.71 (m, 2H), 6.68 (t, *J* = 2.1 Hz, 1H), 3.77 (s, 3H), 2.96 (dd, *J* = 13.5, 6.8 Hz, 1H), 2.82 (m 1H), 2.52 (dd, *J* = 13.5, 7.7 Hz, 1H), 2.08 (d, *J* = 0.5 Hz, 3H), 1.08 (d, *J* = 7.0 Hz, 3H).

^{13}C NMR (101 MHz, $CDCl_3$): *δ* 212.2, 159.7, 141.4, 129.5, 121.4, 114.8, 111.6, 55.2, 48.8, 39.0, 29.0, 16.4.

The NMR data is in agreement with ref. [S1].

*4-(3-Trifluoromethylphenyl)-3-methylbutan-2-one (**4g**)*

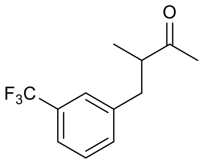

The title compound was prepared according to the general protocol **B** using 4.8 mmol (1.10 g) of ketone **3g** and 0.10 mmol (0.11 g) of 10% (w/w) Pd/C. The product was yielded as a yellow oil (0.61 g; 55%).

^{1}H NMR (400 MHz, $CDCl_3$): *δ* 7.46 – 7.42 (m, 1H), 7.40 – 7.30 (m, 3H), 3.05 (dd, *J* = 13.7, 7.0 Hz, 1H), 2.82 (m, 1H), 2.59 (dd, *J* = 13.7, 7.5 Hz, 1H), 2.09 (d, *J* = 0.5 Hz, 3H), 1.09 (d, *J* = 7.0 Hz, 3H).

^{13}C NMR (101 MHz, $CDCl_3$): δ 211.4, 140.8, 132.5, 130.8, 128.9, 125.6, 124.2, 123.2, 48.61, 38.4, 28.9, 16.4.

^{19}F NMR (376 MHz, $CDCl_3$): δ –62.5 (s, 3F).

3H-indoles ***5a-g***

General protocol ***C***

To a stirred solution of methyl ketone **4** (1.10 – 1.25 eq.) in dry EtOH, phenylhydrazine hydrochloride (1.00 eq.) was added in a single portion. The resulting mixture was heated to reflux and stirred in argon atmosphere for 7 h. The reaction mixture was cooled to room temperature and the solvent was evaporated under reduced pressure. Solid residue was dissolved in DCM, washed with water (2 ×), and dried over $MgSO_4$. The crude product was purified by column chromatography using DCM:MeOH, 97:3 mixture as eluent.

*2,3-Dimethyl-3-benzyl-3H-indole (**5a**)*

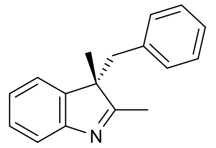

The title compound was prepared according to the general protocol **C** using 1.28 mmol mmol (0.21 g) of ketone **4a** and 1.06 mmol (0.15 g) of phenylhydrazine hydrochloride in 10 mL of dry EtOH. The product was yielded as an orange oil (0.18 g; 71%).

^{1}H NMR (300 MHz, $CDCl_3$) δ 7.43 (d, J = 7.6 Hz, 1H), 7.27 (td, J = 7.5, 1.4 Hz, 1H), 7.17 – 7.03 (m, 5H), 6.87 – 6.28 (m, 2H), 3.18 (d, J = 13.5 Hz, 1H), 2.85 (d, J = 13.5 Hz, 1H), 2.35 (s, 3H), 1.39 (s, 3H).

^{13}C NMR (101 MHz, $CDCl_3$) δ 186.4, 154.2, 143.0, 136.1, 129.4, 127.8, 127.7, 126.7, 124.7, 122.7, 119.8, 58.5, 42.8, 21.9, 16.3.

The NMR data is in agreement with ref. [S4].

*2,3-Dimethyl-3-(4-methylbenzyl)-3H-indole (**5b**)*

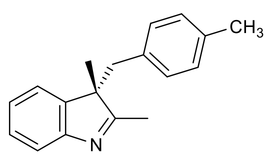

The title compound was prepared according to the general protocol **C** using 5.74 mmol (0.93 g) of ketone **4b** and 4.59 mmol (0.66 g) of phenylhydrazine hydrochloride in 20 mL of dry EtOH. The product was yielded as an orange oil (0.75 g; 68%).

^{1}H NMR (400 MHz, $CDCl_3$) δ 7.44 (d, J = 7.6, 0.9 Hz, 1H), 7.29 – 7.25 (m, 1H), 7.15 (td, J = 7.4, 1.1 Hz, 1H), 7.06 (dt, J = 7.3, 1.8 Hz, 1H), 6.91 (d, J = 7.9 Hz, 2H), 6.69 (d, J = 7.9 Hz, 2H), 3.14 (d, J = 13.5 Hz, 1H), 2.80 (d, J = 13.4 Hz, 1H), 2.33 (s, 3H), 2.23 (s, 3H), 1.37 (s, 3H).

^{13}C NMR (101 MHz, $CDCl_3$) δ 186.6, 154.1, 143.1, 136.3, 136.0, 129.3, 127.7, 127.6, 124.8, 122.5, 119.4, 58.4, 43.0, 22.5, 21.7, 16.4.

The NMR data is in agreement with ref. [S5].

*2,3-Dimethyl-3-(4-methoxybenzyl)-3H-indole (**5c**)*

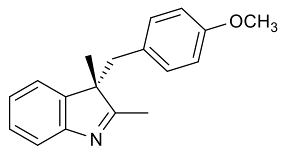

The title compound was prepared according to the general protocol **C** using 2.29 mmol (0.44 g) of ketone **4c** and 2.08 mmol (0.30 g) of phenylhydrazine hydrochloride in 20 mL of dry EtOH. The product was yielded as an orange oil (0.32 g; 58%).

^{1}H NMR (300 MHz, $CDCl_3$) δ 7.43 (dt, J = 7.6, 0.9 Hz, 1H), 7.32 – 7.22 (m, 1H), 7.20 – 7.11 (m, 1H), 7.05 (dt, J = 7.3, 1.3 Hz, 1H), 6.70 (d, J = 8.8 Hz, 2H), 6.63 (d, J = 8.8 Hz, 2H), 3.71 (s, 3H), 3.11 (d, J = 13.6 Hz, 1H), 2.78 (d, J = 13.7 Hz, 1H), 2.33 (s, 3H), 1.36 (s, 3H).

^{13}C NMR (101 MHz, $CDCl_3$) δ 186.7, 158.3, 153.9, 143.1, 130.4, 128.1, 127.7, 124.8, 122.7, 119.8, 113.2, 58.6, 55.1, 42.0, 21.8, 16.3.

*2,3-Dimethyl-3-(4-trifluoromethylbenzyl)-3H-indole (**5d**)*

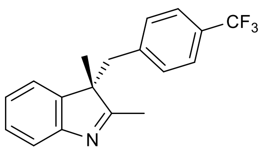

The title compound was prepared according to the general protocol **C** using 2.17 mmol (0.50 g) of ketone **4d** and 1.97 mmol (0.29 g) of phenylhydrazine hydrochloride in 20 mL of dry EtOH. The product was yielded as an orange oil (0.30 g; 50%).

^{1}H NMR (400 MHz, $CDCl_3$) δ 7.41 (d, *J* = 7.6, 1H), 7.37 – 7.30 (m, 2H), 7.27 (dt, *J* = 7.6, 1.0 Hz, 1H), 7.18 (dt, *J* = 7.4, 0.9 Hz, 1H), 7.09 (td, *J* = 7.4, 1.4 Hz, 1H), 6.86 (d, *J* = 7.9 Hz, 2H), 3.23 (d, *J* = 13.5 Hz, 1H), 2.93 (d, *J* = 13.5 Hz, 1H), 2.35 (s, 3H), 1.40 (s, 3H).

^{13}C NMR (101 MHz, $CDCl_3$) δ 185.6, 154.1, 142.4, 140.1, 129.5, 128.9 (q, *J* = 32.4 Hz), 128.1, 125.0, 124.7 (q, *J* = 272 Hz), 124.1 (q, *J* = 3.8 Hz) 122.4, 120.1, 58.3, 42.5, 22.0, 16.2.

The NMR data is in agreement with ref. [S5].

*2,3-Dimethyl-3-(3-methylbenzyl)-3H-indole (**5e**)*

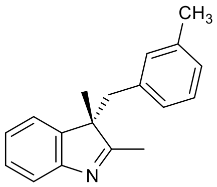

The title compound was prepared according to the general protocol **C** using 2.27 mmol (0.40 g) of ketone **4e** and 2.06 mmol (0.30 g) of phenylhydrazine hydrochloride in 10 mL of dry EtOH. The product was yielded as an orange oil (0.34 g; 66%).

^{1}H NMR (400 MHz, $CDCl_3$): δ 7.42 (dt, *J* = 7.7, 0.9 Hz, 1H), 7.26 (td, *J* = 7.5, 1.3 Hz, 1H), 7.13 (td, *J* = 7.5, 1.1 Hz, 1H), 7.04 – 6.90 (m, 3H), 6.63 – 6.54 (m, 2H), 3.12 (d, *J* = 13.5 Hz, 1H), 2.77 (d, *J* = 13.5 Hz, 1H), 2.31 (s, 3H), 2.17 (d, *J* = 0.7 Hz, 3H), 1.36 (s, 3H).

^{13}C NMR (101 MHz, $CDCl_3$): δ 186.5, 154.4, 143.3, 137.3, 136.2, 130.5, 127.8, 127.7, 127.5, 126.6, 124.6, 122.8, 119.9, 58.5, 42.8, 22.0, 21.4, 16.4.

*2,3-Dimethyl-3-(3-methoxybenzyl)-3H-indole (**5f**)*

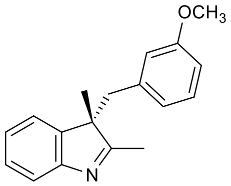

The title compound was prepared according to the general protocol **C** using 2.29 mmol (0.44 g) of ketone **4f** and 2.08 mmol (0.30 g) of phenylhydrazine hydrochloride in 20 mL of dry EtOH. The product was yielded as an orange oil (0.30 g; 54%).

^{1}H NMR (400 MHz, $CDCl_3$): δ 7.42 (dt, *J* = 7.7, 0.9 Hz, 1H), 7.29 – 7.24 (m, 1H), 7.16 (td, *J* = 7.3, 1.1 Hz, 1H), 7.11 (ddd, *J* = 7.4, 1.4, 0.7 Hz, 1H), 7.03 – 6.98 (m, 1H), 6.65 (ddd, *J* = 8.2, 2.6, 0.9 Hz, 1H), 6.44 – 6.40 (m, 1H), 6.23 – 6.20 (m, 1H), 3.56 (s, 3H), 3.17 (d, *J* = 13.5 Hz, 1H), 2.83 (d, *J* = 13.5 Hz, 1H), 2.31 (s, 3H), 1.38 (s, 3H).

^{13}C NMR (101 MHz, $CDCl_3$): δ 186.4, 159.0, 154.5, 143.3, 137.8, 128.8, 127.9, 124.8, 122.7, 121.9, 120.0, 114.6, 112.7, 58.5, 55.0, 42.9, 22.2, 16.4.

The NMR data is in agreement with ref. [S4].

*2,3-Dimethyl-3-(3-trifluoromethylbenzyl)-3H-indole (**5g**)*

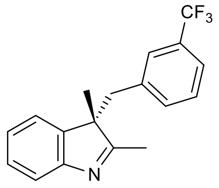

The title compound was prepared according to the general protocol **C** using 1.52 mmol (0.35 g) of ketone **4g** and 1.38 mmol (0.20 g) of phenylhydrazine hydrochloride in 20 mL of dry EtOH. The product was yielded as an orange oil (0.17 g; 40%).

^{1}H NMR (400 MHz, $CDCl_3$): *δ* 7.39 (dt, *J* = 7.8, 0.9 Hz, 1H), 7.35 (d, *J* = 7.8 Hz, 1H), 7.29 – 7.25 (m, 1H), 7.21 – 7.15 (m, 2H), 7.07 (ddd, *J* = 7.4, 1.4, 0.7 Hz, 1H), 6.98 (s, 1H), 6.91 (d, *J* = 7.9 Hz, 1H), 3.22 (d, *J* = 13.5 Hz, 1H), 2.91 (d, *J* = 13.5 Hz, 1H), 2.33 (s, 3H), 1.40 (s, 3H).

^{13}C NMR [^{19}F] (101 MHz, $CDCl_3$): *δ* 185.5, 154.4, 142.4, 137.0, 132.5, 130.1, 128.3, 128.2, 126.2, 125.0, 124.0, 123.7, 122.5, 120.1, 58.5, 42.7, 21.8, 16.3.

^{19}F NMR (376 MHz, $CDCl_3$): *δ* –62.7.

*<u>Indolium salts **6a-g**</u>*

*General protocol **D***

Iodomethane (4.00 eq.) was added dropwise to a stirred solution of 3*H*-indole **5** (1.00 eq.) in dry acetonitrile under argon atmosphere. The obtained solution was heated to 60 °C and stirred overnight (16 h). The reaction mixture was cooled down to 0 °C. Et_2O was slowly added dropwise until visible precipitation of solids. The solids were filtered, washed with Et_2O, dried in vacuo, and used in the next reaction step without further purification.

*1,2,3-Trimethyl-3-benzyl-3H-indolium iodide (**6a**)*

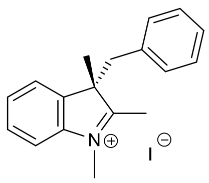

The title compound was prepared according to the general protocol **D** using 0.71 mmol (0.17 g) of 3*H*-indol **5a** and 2.86 mmol (0.18 mL) of iodomethane in 5 mL of dry acetonitrile. The product was yielded as a pale brown solid (0.13 g; 48%) and used in the next reaction step without further purification.

^{1}H NMR (400 MHz, DMSO-*d*6): 7.73 – 7.49 (m, 4H), 7.13 – 6.98 (m, 3H), 6.76 – 6.70 (m, 2H), 3.80 (s, 3H), 3.50 (d, *J* = 13.4 Hz, 1H), 3.40 (d, *J* = 13.4 Hz, 1H), 2.92 (s, 3H), 1.63 (s, 3H).

The NMR data is in agreement with ref. [S6].

*1,2,3-Trimethyl-3-(4-methylbenzyl)-3H-indolium iodide (**6b**)*

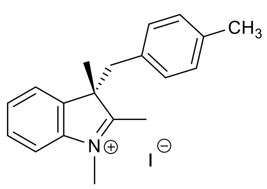

The title compound was prepared according to the general protocol **D** using 2.61 mmol (0.65 g) of 3*H*-indol **5b** and 10.4 mmol (0.65 mL) of iodomethane in 25 mL of dry acetonitrile. The product was yielded as a pale brown solid (0.79 g; 77%)

^{1}H NMR (400 MHz, DMSO-*d*6): *δ* 7.75 – 7.69 (m, 2H), 7.60 – 7.51 (m, 2H), 6.87 (d, *J* = 9.0 Hz, 2H), 6.64 (d, *J* = 8.9 Hz, 2H), 3.84 (s, 3H), 3.48 (d, *J* = 13.6 Hz, 1H), 3.37 (d, *J* = 13.5 Hz, 1H), 2.94 (s, 3H), 2.13 (s, 3H), 1.63 (s, 3H).

*1,2,3-Trimethyl-3-(4-methoxybenzyl)-3H-indolium iodide (**6c**)*

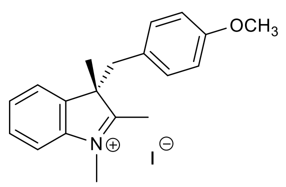

The title compound was prepared according to the general protocol **D** using 0.66 mmol (0.17 g) of 3*H*-indol **5c** and 2.63 mmol (0.16 mL) of iodomethane in 6 mL of dry acetonitrile. The product was yielded as a pale brown solid (0.14 g; 53%) and used in the next reaction step without further purification.

^{1}H NMR (400 MHz, DMSO-*d*6): *δ* 7.74 – 7.67 (m, 2H), 7.63 – 7.52 (m, 2H), 6.66 (d, *J* = 8.1 Hz, 2H), 6.62 (d, *J* = 8.1 Hz, 2H), 3.82 (s, 3H), 3.61 (s, 3H), 3.44 (d, *J* = 13.7 Hz, 1H), 3.33 (d, *J* = 13.6 Hz, 1H), 2.90 (s, 3H), 1.61 (s, 3H).

*1,2,3-Trimethyl-3-(4-trifluoromethylbenzyl)-3H-indolium iodide (**6d**)*

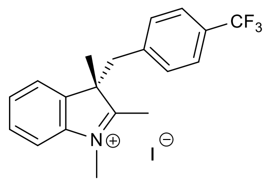

The title compound was prepared according to the general protocol **D** using 1.0 mmol (0.30 g) of 3*H*-indol **5d** and 4.0 mmol (0.25 mL) of iodomethane in 8 mL of dry acetonitrile. The product was yielded as a pale brown solid (0.15 g; 34%) and used in the next reaction step without further purification.

^{1}H NMR (400 MHz, DMSO-*d*6): *δ* 7.76 – 7.69 (m, 2H), 7.63 – 7.52 (m, 2H), 6.66 (d, *J* = 8.1 Hz, 2H), 6.62 (d, *J* = 8.1 Hz, 2H), 3.82 (s, 3H), 3.61 (s, 3H), 3.44 (d, *J* = 13.7 Hz, 1H), 3.33 (d, *J* = 13.6 Hz, 1H), 2.90 (s, 3H), 1.61 (s, 3H).

*1,2,3-Trimethyl-3-(3-methylbenzyl)-3H-indolium iodide (**6e**)*

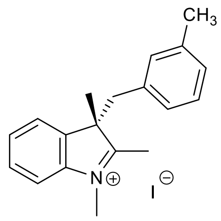

The title compound was prepared according to the general protocol **D** using 1.28 mmol (0.32 g) of 3*H*-indol **5e** and 5.1 mmol (0.32 mL) of iodomethane in 10 mL of dry acetonitrile. The product was yielded as a pale brown solid (0.47 g; 94%) and used in the next reaction step without further purification.

^{1}H NMR (400 MHz, DMSO-*d*6): *δ* 7.73 – 7.66 (m, 2H), 7.61 – 7.52 (m, 2H), 6.99 – 6.88 (m, 2H), 6.54 – 6.49 (m, 2H), 3.80 (s, 3H), 3.49 – 3.30 (m, 2H), 2.90 (s, 3H), 2.08 (s, 3H), 1.63 (s, 3H).

*1,2,3-Trimethyl-3-(3-methoxybenzyl)-3H-indolium iodide (**6f**)*

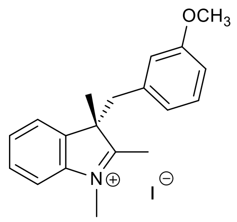

The title compound was prepared according to the general protocol **D** using 0.72 mmol (0.19 g) of 3*H*-indol **5f** and 2.86 mmol (0.18 mL) of iodomethane in 5 mL of dry acetonitrile. The product was yielded as a pale brown solid (0.18 g; 62%) and used in the next reaction step without further purification.

^{1}H NMR (400 MHz, DMSO-*d*6): *δ* 7.74 (dd, *J* = 13.3, 6.9 Hz, 2H), 7.64 – 7.54 (m, 2H), 7.00 (t, *J* = 7.9 Hz, 1H), 6.67 (dd, *J* = 7.9, 2.5 Hz, 1H), 6.34 (d, *J* = 7.6 Hz, 1H), 6.22 – 6.19 (t, *J* = 2.1 Hz, 1H), 3.81 (s, 3H), 3.51 (s, 3H), 3.49 – 3.35 (m, 2H), 2.90 (s, 3H), 1.63 (s, 3H).

*1,2,3-Trimethyl-3-(3-trifluoromethylbenzyl)-3H-indolium iodide (**6g**)*

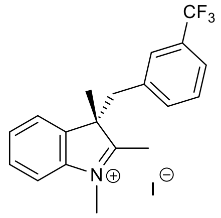

The title compound was prepared according to the general protocol **D** using 0.42 mmol (0.13 g) of 3*H*-indol **5g** and 1.9 mmol (0.12 mL) of iodomethane in 5 mL of dry acetonitrile. The product was yielded as a pale brown solid (0.18 g; 96%) and used in the next reaction step without further purification.

^{1}H NMR (400 MHz, DMSO-*d*6): *δ* 7.71 (t, *J* = 7.1 Hz, 2H), 7.63 – 7.53 (m, 2H), 7.48 (d, *J* = 7.9 Hz, 1H), 7.35 (t, *J* = 7.7 Hz, 1H), 7.13 (d, *J* = 7.7 Hz, 1H), 6.99 (s, 1H), 3.80 (s, 3H), 3.66 – 3.48 (m, 2H), 2.93 (s, 3H), 1.65 (s, 3H).

*Spiropyrans **Ia-g***

*1',3'-Dimethyl-3'-benzyl-6-nitrospiro[chromene-2,2'-indoline] (**Ia**)*

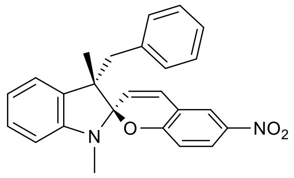

Triethylamine (2.00 mmol, 0.28 mL) was added in one portion into a solution of indolium salt **6a** (1.00 mmol, 377 mg) and 2-hydroxy-5-nitrobenzaldehyde (1.00 mmol, 167 mg) in ethanol (15 mL). The obtained mixture was heated to reflux and stirred for 2.5 hours. The solvent was evaporated under reduced pressure. The yielded residue was suspended in Et_2O (30 mL) and solids were filtered off.The filtrate was evaporated and the crude product was purified by two consecutive column chromatographies on basic alumina (Brockmann I type) using DCM:MeOH, 99:1 mixture as eluent. 224 mg (56 %) of **Ia** (yellow solid) was isolated.

^{1}H NMR (400 MHz, $CDCl_3$, diastereomeric ratio *syn:anti* = 25:75) δ 8.11 (dd, *J* = 9.0, 2.7 Hz, 1H), 8.06 – 7.98 (m, 3H), 7.26 – 7.08 (m, 8H) 7.05 – 7.02 (m, 1H), 7.01 (d, *J* = 10.5 Hz, 1H), 6.94 – 6.87 (m, 2H), 6.78 – 6.75 (m 2H), 6.72 – 6.66 (m, 5H), 6.64 (d, *J* = 8.5 Hz, 1H), 6.56 (d, *J* = 7.8 Hz, 1H), 6.19 (dt, *J* = 7.3, 0.7 Hz, 1H), 6.04 (d, *J* = 10.3 Hz, 1H), 5.65 (d, *J* = 10.3 Hz, 1H), 3.26 (d, *J* = 13.6 Hz, 1H), 3.17 (d, *J* = 13.6 Hz, 1H), 2.83 (s, 3H), 2.80 (d, *J* = 12.3 Hz, 1H), 2.71 (d, *J* = 12.3 Hz, 1H), 2.62 (s, 3H), 1.30 (s, 3H), 1.23 (s, 3H).

^{13}C NMR (101 MHz, $CDCl_3$) δ 160.0, 159.1, 148.2, 146.9, 141.1, 141.0, 137.4, 136.7, 135.7, 132.0, 131.6, 130.9, 128.8, 128.0, 127.6, 127.3, 127.0, 126.4, 126.4, 126.0, 125.8, 125.0, 122.7, 122.7, 122.2, 121.8, 120.9, 119.8, 119.2, 118.8, 118.6, 115.7, 115.6, 107.7, 107.3, 107.1, 104.6, 57.3, 54.4, 41.5, 39.3, 29.3, 28.1, 23.8, 16.6.

HRMS (APCI+): calculated for $[C_{25}H_{22}N_2O_3 + H]^+$: 399.1703; found: 399.1699.

*1',3'-Dimethyl-3'-(4-methylbenzyl)-6-nitrospiro[chromene-2,2'-indoline] (**Ib**)*

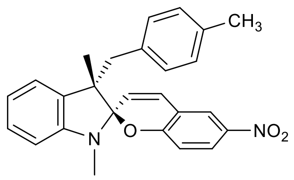

Triethylamine (1.00 mmol, 0.14 mL) was added in one portion into a solution of indolium salt **6b** (0.50 mmol, 196 mg) and 2-hydroxy-5-nitrobenzaldehyde (0.50 mmol, 84 mg) in ethanol (10 mL). The obtained mixture was heated to reflux and stirred for 2 hours. The solvent was evaporated under reduced pressure. The yielded residue was dissolved in DCM (30 mL), washed with water (3 × 15 mL) and dried over $MgSO_4$. The crude product was purified by two consecutive column chromatographies on neutral alumina (Brockmann I type) using hexane:DCM, 1:1 and DCM:MeOH, 99:1 mixtures as eluents. 140 mg (68 %) of **Ib** (light-brown solid) was isolated.

^{1}H NMR (400 MHz, $CDCl_3$, diastereomeric ratio *syn:anti* = 25:75) δ 8.11 (dd, *J* = 9.0, 2.7 Hz, 1H), 8.07 – 7.99 (m, 3H), 7.24 (td, *J* = 7.7, 1.5 Hz, 2H), 7.11 (dd, *J* = 7.4, 1.3 Hz, 1H), 7.03 – 6.97 (m, 4H), 6.95 – 6.91 (m, 4H), 6.89 (dd, *J* = 7.5, 1.0 Hz, 1H), 6.77 (d, *J* = 9.7 Hz, 1H), 6.71 (td, *J* = 7.4, 1.0 Hz, 2H), 6.64 (d, *J* = 7.7 Hz, 1H), 6.60 (d, *J* = 8.0 Hz, 2H), 6.56 (d, *J* = 7.7, 1H), 6.24 (dd, *J* = 7.3, 1.3 Hz, 1H), 6.05 (d, *J* = 10.4 Hz, 1H), 5.68 (d, *J* = 10.3 Hz, 1H), 3.23 (d, *J* = 13.6 Hz, 1H), 3.14 (d, *J* = 13.6 Hz, 1H), 2.84 (s, 3H), 2.78 (d, *J* = 13.2 Hz, 1H), 2.68 (d, *J* = 13.2 Hz, 1H), 2.63 (s, 3H), 2.34 (s, 3H), 2.30 (s, 3H), 1.45 (s, 3H), 1.23 (s, 3H).

^{13}C NMR (101 MHz, $CDCl_3$) δ 160.0, 159.2 148.2, 147.0, 141.1, 141.0, 135.9, 135.9, 135.8, 134.2, 133.5, 132.2, 131.5, 130.8, 128.7, 128.3, 128.0, 128.0, 128.0, 127.9, 127.0, 126.0, 125.8, 125.1, 122.7, 122.7, 122.3, 121.9, 121.0, 119.7, 118.8, 118.6, 115.7, 115.6, 107.7, 107.3, 107.1, 104.6, 57.3, 54.4, 41.1, 38.8, 29.3, 26.9, 21.1, 21.0, 16.6, 14.2.

HRMS (APCI+): calculated for $[C_{26}H_{24}N_2O_3 + H]^+$: 413.1860; found: 413.1865.

*1',3'-Dimethyl-3'-(4-methoxybenzyl)-6-nitrospiro[chromene-2,2'-indoline] (**Ic**)*

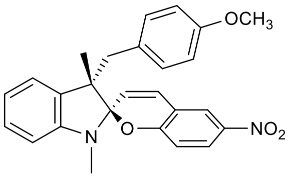

Triethylamine (0.70 mmol, 0.10 mL) was added in one portion into a solution of indolium salt **6c** (0.35 mmol, 141 mg) and 2-hydroxy-5-nitrobenzaldehyde (0.35 mmol, 58 mg) in ethanol (10 mL). The obtained mixture was heated to reflux and stirred for 2 hours. The solvent was evaporated under reduced pressure. The yielded residue was dissolved in DCM (30 mL), washed with water (3 × 15 mL) and dried over $MgSO_4$. The crude product was purified by two consecutive column chromatographies on neutral alumina (Brockmann I type) using hexane:DCM, 1:1 and DCM:MeOH, 99:1 mixtures as eluents. 120 mg (80 %) of **Ic** (purple semi-solid) was isolated.

^{1}H NMR (400 MHz, $CDCl_3$, diastereomeric ratio *syn:anti* = 24:76) *δ* 8.10 (dd, *J* = 9.0, 2.7 Hz, 1H), 8.04 – 7.98 (m, 3H), 7.22 (td, *J* = 7.7, 1.3 Hz, 2H), 7.08 (dd, *J* = 7.3, 1.3, 1H), 6.99 (d, *J* = 10.5, 1H), 6.96 – 6.86 (m, 3H), 6.79 – 6.74 (m, 1H), 6.74 – 6.66 (m, 5H), 6.66 – 6.58 (m, 5H), 6.55 (d, J = 8.5 Hz, 1H), 6.22 (dd, *J* = 7.3, 1.3, 1H), 6.02 (d, *J* = 10.4 Hz, 1H), 5.65 (d, *J* = 10.3 Hz, 1H), 3.79 (s, 3H), 3.75 (s, 3H), 3.19 (d, *J* = 13.8 Hz, 1H), 3.10 (d, *J* = 13.8 Hz, 1H), 2.81 (s, 3H), 2.74 (d, *J* = 12.3 Hz, 1H), 2.63 (d, *J* = 12.5 Hz, 1H), 2.60 (s, 3H), 1.27 (s, 3H), 1.20 (s, 3H).

^{13}C NMR (101 MHz, $CDCl_3$) *δ* 160.0, 159.1, 158.2, 158.2, 148.2, 147.0, 141.0, 135.7, 132.5, 132.1, 131.8, 129.4, 128.7, 128.7, 128.0, 128.0, 126.9, 126.0, 125.8, 125.0, 122.7, 122.6, 122.2, 121.9, 121.0, 119.7, 119.2, 118.8, 118.6, 115.7, 115.6, 112.9, 112.7, 107.6, 107.3, 107.0, 104.6, 57.4, 55.2, 54.4, 40.6, 38.3, 29.3, 28.1, 23.7, 16.5.

HRMS (APCI+): calculated for $[C_{26}H_{24}N_2O_4 + H]^+$: 429.1809; found: 429.1810.

*1',3'-Dimethyl-3'-(4-trifluoromethylbenzyl)-6-nitrospiro[chromene-2,2'-indoline] (**Id**)*

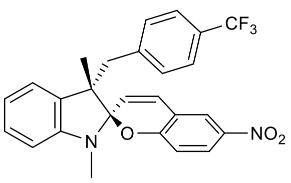

Triethylamine (1.00 mmol, 0.14 mL) was added in one portion into a solution of indolium salt **6d** (0.50 mmol, 152 mg) and 2-hydroxy-5-nitrobenzaldehyde (0.50 mmol, 84 mg) in ethanol (10 mL). The obtained mixture was heated to reflux and stirred for 2 hours. The solvent was evaporated under reduced pressure. The yielded residue was dissolved in DCM (30 mL), washed with water (3 × 15 mL) and dried over $MgSO_4$. The crude product was purified by two consecutive column chromatographies on neutral alumina (Brockmann I type) using hexane:DCM, 1:1 and DCM:MeOH, 99:1 mixtures as eluents. 138 mg (60 %) of **Id** (purple solid) was isolated.

^{1}H NMR (400 MHz, $CDCl_3$, diastereomeric ratio *syn:anti* = 25:75) *δ* 8.11 (dd, *J* = 9.0, 2.7 Hz, 1H), 8.05 – 8.00 (m, 3H), 7.43 (d, *J* = 8.0 Hz, 2H), 7.31 (d, *J* = 7.9 Hz, 1H), 7.27 – 7.21 (m, 3H), 7.13 (d, *J* = 8.0 Hz, 1H), 7.07 – 6.99 (m, 2H), 6.95 – 6.87 (m, 2H), 6.82 – 6.75 (m, 4H), 6.74 – 6.67 (m, 2H), 6.64 (d, *J* = 7.8 Hz, 1H), 6.55 (d, *J* = 7.7 Hz, 1H), 6.16 (dd, *J* = 7.3, 1.2 Hz, 1H), 6.01 (d, *J* = 10.4 Hz, 1H), 5.64 (d, *J* = 10.3 Hz, 1H), 3.31 (d, *J* = 13.5 Hz, 1H), 3.21 (d, *J* = 13.5 Hz, 1H), 2.84 (d, *J* = 12.2 Hz, 1H), 2.82 (s, 3H), 2.76 (d, *J* = 12.2 Hz, 1H), 2.60 (s, 3H), 1.28 (s, 3H), 1.20 (s, 3H).

^{13}C NMR [^{19}F] (101 MHz, DMSO-*d6*) *δ* 159.9, 158.9, 148.5, 146.9, 142.2, 141.2, 141.1, 135.9, 132.4, 131.7, 131.7, 129.4, 128.6, 128.5, 127.6, 126.3, 126.2, 126.2, 124.6, 124.5, 124.4, 124.4, 123.3, 123.3, 122.4, 121.9, 120.8, 120.0, 119.7, 119.5, 118.7, 116.1, 115.9, 107.8, 107.8, 104.3, 57.0, 54.3, 41.1, 39.1, 31.1, 29.3, 28.0, 16.7.

^{19}F NMR (376 MHz, $CDCl_3$): *δ* –62.2, –62.3.

HRMS (APCI+): calculated for $[C_{26}H_{21}F_3N_2O_3 + H]^+$: 467.1577; found: 467.1574.

*1',3'-Dimethyl-3'-(3-methylbenzyl)-6-nitrospiro[chromene-2,2'-indoline] (**Ie**)*

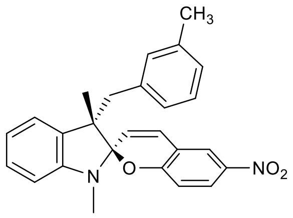

Triethylamine (2.20 mmol, 0.31 mL) was added in one portion into a solution of indolium salt **6e** (1.10 mmol, 430 mg) and 2-hydroxy-5-nitrobenzaldehyde (1.10 mmol, 184 mg) in ethanol (20 mL). The obtained mixture was heated to reflux and stirred for 2 hours. The solvent was evaporated under reduced pressure. The yielded residue was dissolved in DCM (30 mL), washed with water (3 × 15 mL) and dried over $MgSO_4$. The crude product was purified by two consecutive column chromatographies on neutral alumina (Brockmann I type) using hexane:DCM, 1:1 and DCM:MeOH, 99:1 mixtures as eluents. 342 mg (72 %) of **Ie** (light-brown solid) was isolated.

^{1}H NMR (400 MHz, $CDCl_3$, diastereomeric ratio *syn:anti* = 27:73): δ 8.10 (dd, *J* = 9.0, 2.7 Hz, 1H), 8.03 – 8.01 (m, 2H), 7.98 (dd, *J* = 10.1, 2.7 Hz, 1H), 7.22 (td, J = 7.7, 1.3 Hz, 1H), 7.21 (td, *J* = 7.7, 1.3 Hz, 1H), 7.10 (dd, *J* = 7.3, 1.3 Hz, 1H), 7.08 – 6.96 (m, 4H), 6.94 – 6.84 (m, 4H), 6.78 – 6.72 (m, 2H), 6.67 (td, *J* = 7.5, 1.0 Hz, 1H), 6.64 – 6.60 (m, 2H), 6.54 (d, *J* = 7.7 Hz, 1H), 6.49 (dt, J = 7.4, 1.7 Hz, 1H), 6.46 – 6.43 (m, 1H), 6.18 (ddd, *J* = 7.3, 1.3, 0.5 Hz, 1H), 6.02 (d, *J* = 10.4 Hz, 1H), 5.61 (d, *J* = 10.3 Hz, 1H), 3.22 (d, *J* = 13.5 Hz, 1H), 3.11 (d, *J* = 13.5 Hz, 1H), 2.81 (s, 3H), 2.74 (d, *J* = 12.1 Hz, 1H), 2.65 (d, *J* = 12.1 Hz, 1H), 2.59 (s, 3H), 2.22 (s, 3H), 2.02 (s, 3H), 1.28 (s, 3H), 1.20 (s, 3H).

^{13}C NMR (101 MHz, $CDCl_3$): δ 160.1, 159.3, 148.3, 147.0, 141.2, 141.1, 137.3, 137.1, 136.8, 136.7, 136.0, 132.7, 132.2, 132.0, 128.8, 128.7, 128.1, 127.8, 127.5, 127.2, 127.2, 127.1, 126.8, 126.1, 125.9, 125.2, 122.8, 122.7, 122.2, 122.0, 121.0, 119.9, 119.3, 118.9, 118.6, 115.8, 115.7, 107.8, 107.4, 107.1, 104.6, 57.4, 54.1, 41.5, 39.3, 29.4, 28.1, 24.0, 21.4, 21.2, 16.7.

HRMS (APCI+): calculated for $[C_{26}H_{24}N_2O_3 + H]^+$: 413.1860; found: 413.1856.

*1',3'-Dimethyl-3'-(3-methoxybenzyl)-6-nitrospiro[chromene-2,2'-indoline] (**If**)*

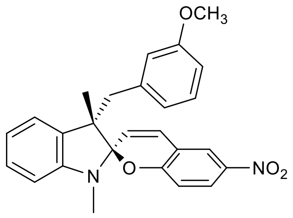

Triethylamine (0.84 mmol, 0.12 mL) was added in one portion into a solution of indolium salt **6f** (0.42 mmol, 170 mg) and 2-hydroxy-5-nitrobenzaldehyde (0.70 mmol, 42 mg) in ethanol (10 mL). The obtained mixture was heated to reflux and stirred for 2 hours. The solvent was evaporated under reduced pressure. The yielded residue was dissolved in DCM (30 mL), washed with water (3 × 15 mL) and dried over $MgSO_4$. The crude product was purified by two consecutive column chromatographies on neutral alumina (Brockmann I type) using hexane:DCM, 1:1 and DCM:MeOH, 99:1 mixtures as eluents. 110 mg (62 %) of **If** (purple solid) was isolated.

^{1}H NMR (400 MHz, $CDCl_3$): δ 8.09 (dd, *J* = 9.0, 2.7 Hz, 1H), 8.04 – 7.97 (m, 3H), 7.25 – 7.19 (m, 2H), 7.13 – 6.86 (m, 5H), 6.79 – 6.60 (m, 8H), 6.5 – 6.49 (m, 1H), 6.39 – 6.34 (m, 2H), 6.28 – 6.23 (m, 1H), 6.15 – 6.1 (m, 1H), 6.02 (d, *J* = 10.3 Hz, 1H), 5.66 (d, *J* = 10.2 Hz, 1H), 3.63 (s, 3H), 3.53 (s, 3H), 3.23 (d, *J* = 13.6 Hz, 1H), 3.12 (d, *J* = 13.7 Hz, 1H), 2.81 (s, 3H), 2.77 (d, *J* = 12.1 Hz, 1H), 2.68 (d, *J* = 12.1 Hz, 1H), 2.60 (s, 3H), 1.30 (s, 3H), 1.23 (s, 3H).

^{13}C NMR (101 MHz, $CDCl_3$) δ 160.1, 159.2, 159.0, 158.6, 148.3, 147.0, 141.1, 138.4, 135.8, 132.1, 129.0, 128.6, 128.30, 128.2, 128.2, 127.0, 126.1, 125.9, 125.2, 124.0, 123.5, 122.8, 122.8, 122.3, 121.9, 121.0, 119.9, 119.4, 118.9, 118.7, 116.9, 116.6, 115.8, 115.7, 112.6, 112.0, 107.8, 107.4, 107.2, 104.6, 57.4, 55.1, 54.9, 41.7, 39.4, 29.4, 28.1, 24.0, 16.8.

HRMS (APCI+): calculated for $[C_{26}H_{24}N_2O_4 + H]^+$: 429.1809; found: 429.1807.

1',3'-Dimethyl-3'-(3-trifluoromethylbenzyl)-6-nitrospiro[chromene-2,2'-indoline] ***(Ig)***

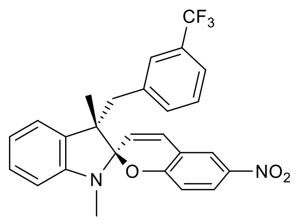

Triethylamine (0.76 mmol, 0.11 mL) was added in one portion into a solution of indolium salt **6g** (0.38 mmol, 170 mg) and 2-hydroxy-5-nitrobenzaldehyde (0.38 mmol, 64 mg) in ethanol (10 mL). The obtained mixture was heated to reflux and stirred for 2 hours. The solvent was evaporated under reduced pressure. The yielded residue was dissolved in DCM (30 mL), washed with water (3 × 15 mL) and dried over $MgSO_4$. The crude product was purified by two consecutive column chromatographies on neutral alumina (Brockmann I type) using hexane:DCM, 1:1 and DCM:MeOH, 99:1 mixtures as eluents. 121 mg (68 %) of **Ig** (purple solid) was isolated.

^{1}H NMR (400 MHz, $CDCl_3$): *δ* 8.11 (dd, *J* = 9.0, 2.7 Hz, 1H), 8.05 – 8.02 (m, 2H), 8.00 (dd, *J* = 7.5, 2.7 Hz, 1H), 7.49 – 7.45 (m, 2H), 7.42 – 7.38 (m, 1H), 7.33 – 7.17 (m, 4H), 7.1 – 6.97 (m, 3H), 6.95 – 6.85 (m, 4H), 6.77 (d, *J* = 8.7 Hz, 1H), 6.72 – 6.62 (m, 3H), 6.55 (d, *J* = 7.8 Hz, 1H), 6.13 (d, *J* = 7.3 Hz, 1H), 6.02 (d, *J* = 10.2 Hz, 1H), 5.59 (d, *J* = 10.3 Hz, 1H), 3.37 (d, *J* = 13.8 Hz, 1H), 3.21 (d, *J* = 13.7 Hz, 1H), 2.85 (d, *J* = 12.3 Hz, 1H), 2.82 (s, 3H), 2.77 (d, *J* = 12.3 Hz, 1H), 2.59 (s, 3H), 1.29 (s, 3H), 1.20 (s, 3H).

^{13}C NMR [^{19}F] (101 MHz, $CDCl_3$): *δ* 159.9, 158.7, 148.3, 146.9, 141.5, 141.2, 138.5, 137.8, 135.3, 134.8, 134.2, 131.4, 130.0, 129.7, 129.2, 128.5, 128.4, 128.3, 128.1, 127.8, 127.5, 127.2, 126.2, 126.0, 124.8, 124.2, 123.4, 123.0, 122.9, 122.0, 121.5, 120.6, 120.0, 119.2, 118.9, 118.8, 115.7, 107.6, 107.5, 107.5, 104.1, 57.2, 54.2, 41.4, 39.3, 29.4, 28.0, 23.7, 16.5.

^{19}F NMR (376 MHz, $CDCl_3$): *δ* –62.6, –62.8.

HRMS (APCI+): calculated for $[C_{26}H_{21}F_3N_2O_3 + H]^+$: 467.1577; found: 467.1579.

2.2 Synthesis of the Series II

*Arylacetones **9a-e***

*General protocol **E***

A solution of arylacetic acid **8** (1.00 eq.), oxalyl chloride (1.50 – 2.10 eq.) and one drop of DMF (ca 0.01 ml) in dry toluene (40 mL) was stirred at room temperature under argon atmosphere for 1 h. The solvent was evaporated under reduced pressure and the solid residue was filtered off. The filtrate was dissolved in DCM (10 mL) and added dropwise to a solution of 2,2-dimethyl-1,3-dioxane-4,6-dione (0.95 – 1.00 eq.) and freshly distilled pyridine (1.90 eq.) in dry DCM (50 ml) at 0 °C under argon atmosphere. After completion of addition, the reaction was warmed to room temperature and stirred for additional 2-3 h. The reaction mixture was then washed successively with 5% HCl solution (3 × 25 mL) and brine (25 mL). The organic layer was dried over anhydrous $MgSO_4$. The crude intermediate was purified by column chromatography on silica gel using hexane:EtOAc, 2:1 mixture as an eluent. The obtained keto ester was dissolved in AcOH (30 mL) and H_2O (25 mL) and refluxed for 2 h. The reaction mixture was cooled down to room temperature and neutralized with a saturated solution of $NaHCO_3$. The aqueous phase was extracted with chloroform (3 × 100 mL) and then the organic layer was dried over anhydrous $MgSO_4$. The product **9** was yielded after solvent evaporation.

*1-Phenylpropan-2-one (**9a**)*

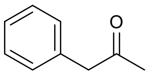

The title compound was prepared according to the general protocol **E** using 14.7 mmol (2.00 g) of **8a**, 22.0 mmol (1.86 mL) of oxalyl chloride, 13.4 mmol (1.93 g) of 2,2-dimethyl-1,3-dioxane-4,6-dione and 26.7 mmol (2.16 mL) of pyridine. The product was yielded as a yellowish oil (0.84 g; 44%).

^{1}H NMR (400 MHz, $CDCl_3$) δ 7.21 – 7.19 (m, 5H), 3.63 (s, 2H), 2.09 (s, 3H).

The NMR data is in agreement with ref. [S7].

*1-(4-Methylphenyl)propan-2-one (**9b**)*

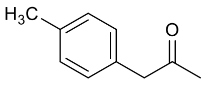

The title compound was prepared according to the general protocol **E** using 13.3 mmol (2.00 g) of **8b**, 23.6 mmol (2.00 mL) of oxalyl chloride, 12.7 mmol (1.83 g) of 2,2-dimethyl-1,3-dioxane-4,6-dione and 25.4 mmol (2.05 mL) of pyridine. The product was yielded as a bright yellow oil (1.06 g; 56%).

^{1}H NMR (400 MHz, $CDCl_3$) δ 7.15 – 7.05 (m, 4H), 4.03 (s, 2H), 2.28 (s, 3H).

The NMR data is in agreement with ref. [S8].

*1-(4-Methoxyphenyl)propan-2-one (**9c**)*

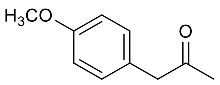

The title compound was prepared according to the general protocol **E** using 12.0 mmol (2.00 g) of **8c**, 18.1 mmol (1.53 mL) of oxalyl chloride, 12.0 mmol (1.73 g) of 2,2-dimethyl-1,3-dioxane-4,6-dione and 24.0 mmol (1.95 mL) of pyridine. The product was yielded as a yellowish oil (1.10 g; 53%).

^{1}H NMR (400 MHz, $CDCl_3$) δ 7.12 (d, J = 8.6 Hz, 2H), 6.87 (d, J = 8.6 Hz, 2H), 3.80 (s, 3H), 3.63 (s, 2H), 2.14 (s, 3H).

The NMR data is in agreement with ref. [S9].

*1-(4-Trifluoromethylphenyl)propan-2-one (**9d**)*

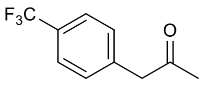

The title compound was prepared according to the general protocol **E** using 4.40 mmol (0.90 g) of **8d**, 6.60 mmol (0.56 mL) of oxalyl chloride, 4.40 mmol (0.64 g) of 2,2-dimethyl-1,3-dioxane-4,6-dione and 8.80 mmol (0.71 mL) of pyridine. The product was yielded as an orange oil (0.39 g; 44%).

^{1}H NMR (400 MHz, $CDCl_3$) δ 7.53 (d, J = 8.0 Hz, 2H), 7.25 (d, J = 8.0 Hz, 2H), 3.72 (s, 2H), 2.14 (s, 3H).

The NMR data is in agreement with ref. [S10].

*1-(4-Nitrophenyl)propan-2-one (**9e**)*

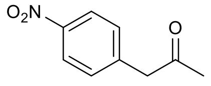

The title compound was prepared according to the general protocol **E** using 11.0 mmol (2.00 g) of **8e**, 23.6 mmol (2.00 mL) of oxalyl chloride, 11.0 mmol (1.59 g) of 2,2-dimethyl-1,3-dioxane-4,6-dione and 22.0 mmol (1.78 mL) of pyridine. The product was yielded as a yellowish oil (3.31 g; 55%).

^{1}H NMR (400 MHz, $CDCl_3$) δ 8.13 (d, J = 8.7 Hz, 2H), 7.30 (d, J = 8.8 Hz, 2H), 3.78 (s, 2H), 2.18 (s, 3H).

The NMR data is in agreement with ref. [S11].

*<u>Ketones **10a-e**</u>*

General protocol ***F***

Iodomethane (15 – 30 eq.) was slowly added dropwise to a mixture of arylacetone **9** (1.00 eq.), 50% aq. NaOH (3.0 – 3.6 mL) and benzyltriethylammonium bromide (0.20 eq.) while the temperature was kept at approx. 20 °C using a cooling bath. The resulting mixture was vigorously stirred at ambient temperature for 24 h. Distilled water (30 mL) and DCM (40 mL) were added. Phases were separated and the organic layer was washed with portions of distilled water until the aqueous layer was pH neutral. The resulting organic phase was washed with brine and dried over $MgSO_4$. The crude product was purified by column chromatography on silica gel using hexane:EtOAc, 8:1 mixture as eluent.

*3-Phenylbutan-2-one (**10a**)*

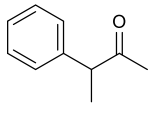

A flame-dried round-bottom flask was charged with 60% dispersion of NaH in mineral oil (2.25 mmol, 90 mg) and dry THF (15 mL) under argon atmosphere. Phenylacetone (**9a**; 0.21 g, 1.56 mmol) in dry THF (10 mL) was added dropwise. The reaction mixture was stirred at room temperature under argon atmosphere for 1 h. Subsequently, iodomethane (11.8 mmol, 0.74 mL) was added and the reaction mixture was stirred at room temperature under argon atmosphere for an additional 1 h. Distilled water (10 mL) was slowly added dropwise. The obtained mixture was extracted with diethyl ether (3 × 20 mL). The organic layer was then washed with brine (10 mL) and dried over anhydrous $MgSO_4$ to yield **10a** as a yellowish oil (0.18 g; 75%).

^{1}H NMR (400 MHz, $CDCl_3$) δ 7.32 – 7.09 (m, 5H), 3.68 (q, J = 7.0 Hz, 1H), 1.98 (s, 3H), 1.32 (d, J = 7.0 Hz, 3H).

The NMR data is in agreement with ref. [S12].

*3-(4-Methylphenyl)butan-2-one (**10b**)*

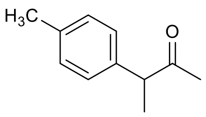

The title compound was prepared according to the general protocol **F** using 2.76 mmol (0.41 g) of **9b**, 3.0 mL of 50% aq. NaOH, 0.55 mmol (127 mg) of benzyltriethylammonium bromide and 82.7 mmol (5.2 mL) of iodomethane. The product was yielded as a bright yellow oil (0.34 g; 77%).

^{1}H NMR (400 MHz, $CDCl_3$) δ 7.08 (d, J = 8.0 Hz, 2H), 7.03 (d, J = 8.0 Hz, 2H), 3.64 (q, J = 7.0 Hz, 1H), 2.27 (s, 3H), 1.97 (s, 3H), 1.30 (d, J = 7.0 Hz, 3H).

The NMR data is in agreement with ref. [S12].

*3-(4-Methoxyphenyl)butan-2-one (**10c**)*

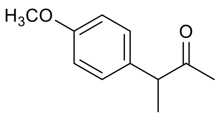

The title compound was prepared according to the general protocol **F** using 1.54 mmol (0.25 g) of **9c**, 3.0 mL of 50% aq. NaOH, 0.31 mmol (84 mg) of benzyltriethylammonium bromide and 23.1 mmol (1.4 mL) of iodomethane. The product was yielded as a bright yellow oil (0.26 g; 96%).

^{1}H NMR (400 MHz, $CDCl_3$) δ 7.05 (d, J = 7.3 Hz, 2H), 6.80 (d, J = 7.3 Hz, 2H), 3.73 (s, 3H), 3.62 (q, J = 7.0 Hz, 1H), 1.97 (s, 3H), 1.29 (d, J = 7.0 Hz, 3H).

The NMR data is in agreement with ref. [S12].

*3-(4-Trifluoromethylphenyl)butan-2-one (**10d**)*

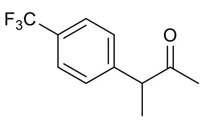

The title compound was prepared according to the general protocol **F** using 1.92 mmol (0.39 g) of **9d**, 3.6 mL of 50% aq. NaOH, 0.38 mmol (104 mg) of benzyltriethylammonium bromide and 57.5 mmol (3.6 mL) of iodomethane. The product was yielded as a bright yellow oil (0.17 g; 42%).

^{1}H NMR (400 MHz, $CDCl_3$) δ 7.53 (d, J = 8.0 Hz, 2H), 7.28 (d, J = 8.0 Hz, 2H), 3.76 (q, J = 7.0 Hz, 1H), 2.01 (s, 3H), 1.35 (d, J = 7.0 Hz, 3H).

The NMR data is in agreement with ref. [S13].

*3-(4-Nitrophenyl)butan-2-one (**10e**)*

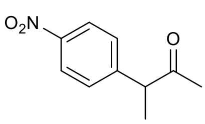

The title compound was prepared according to the general protocol **F** using 1.47 mmol (0.26 g) of **9e**, 3.6 mL of 50% aq. NaOH, 0.29 mmol (80 mg) of benzyltriethylammonium bromide and 44.1 mmol (2.7 mL) of iodomethane. The product was yielded as a bright yellow oil (0.14 g; 48%).

^{1}H NMR (400 MHz, $CDCl_3$) δ 8.14 (d, J = 8.8 Hz, 2H), 7.34 (d, J = 8.8 Hz, 2H), 3.83 (q, J = 7.0 Hz, 1H), 2.04 (s, 3H), 1.38 (d, J = 7.0 Hz, 3H).

The NMR data is in agreement with ref. [S14].

*3H-Indoles **11a-e***

*General protocol **G***

To a stirred solution of ketone **10** (1.05 – 1.10 eq.) in dry EtOH, phenylhydrazine hydrochloride (1.00 eq.) was added in a single portion. The resulting mixture was heated to reflux and stirred in argon atmosphere for 6.5 h. The reaction mixture was cooled to room temperature and the solvent was evaporated under reduced pressure. Solid residue was dissolved in DCM, washed with water (2 ×), and dried over $MgSO_4$. The crude product was purified by column chromatography using hexane:EtOAc, 8:1 mixture as eluent.

*2,3-Dimethyl-3-phenyl-3H-indole (**11a**)*

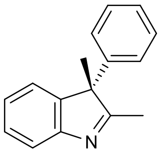

The title compound was prepared according to the general protocol **G** using 1.50 mmol (0.22 g) of **10a** and 1.40 mmol (0.20 g) of phenylhydrazine hydrochloride in 35 mL of dry EtOH. The product was yielded as an orange oil (0.12 g; 37%).

^{1}H NMR (400 MHz, $CDCl_3$) δ 7.56 (dd, J = 7.7; 0.8 Hz, 1H), 7.27 (ddd, J = 7.8; 1.4; 0.7 Hz, 1H), 7.24–7.14 (m, 3H), 7.10 (dd, J = 7.4; 0.9 Hz, 1H), 7.03 (ddd, J = 7.4; 1.3; 0.7 Hz, 1H), 6.98–6.93 (m, 2H), 2.09 (s, 3H), 1.63 (s, 3H).

^{13}C NMR (101 MHz, $CDCl_3$) δ 187.6, 153.7, 146.8, 139.1, 128.9, 128.0, 127.3, 126.1, 125.9, 122.5, 120.0, 61.8, 20.3, 15.8.

The NMR data is in agreement with ref. [S15].

*2,3-Dimethyl-3-(4-methylphenyl)-3H-indole (**11b**)*

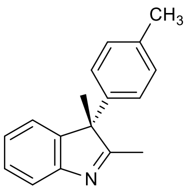

The title compound was prepared according to the general protocol **G** using 0.92 mmol (0.15 g) of **10b** and 0.88 mmol (0.13 g) of phenylhydrazine hydrochloride in 20 mL of dry EtOH. The product was yielded as a brownish oil (0.10 g; 49%).

^{1}H NMR (400 MHz, $CDCl_3$) δ 7.70 (d, J = 7.8 Hz, 1H), 7.37 (td, J = 7.7; 1.3 Hz, 1H), 7.22 (td, J = 7.5; 1.1 Hz, 1H), 7.15 – 7.07 (m, 3H), 6.90 (d, J = 8.3 Hz, 2H), 2.31 (s, 3H), 2.25 (s, 3H), 1.71 (s, 3H).

^{13}C NMR (101 MHz, $CDCl_3$) δ 187.3, 153.8, 146.7, 139.6, 136.0, 129.8, 128.5, 127.4, 125.9, 122.8, 119.2, 61.5, 21.2, 20.3, 15.3.

*2,3-Dimethyl-3-(4-methoxyphenyl)-3H-indole (**11c**)*

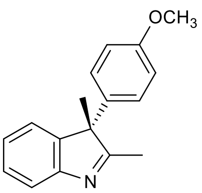

The title compound was prepared according to the general protocol **G** using 1.47 mmol (0.26 g) of **10c** and 1.40 mmol (0.20 g) of phenylhydrazine hydrochloride in 30 mL of dry EtOH. The product was yielded as an orange oil (0.13 g; 38%).

^{1}H NMR (400 MHz, $CDCl_3$) δ 8.02 (d, J = 7.8 Hz, 1H), 7.55 (td, J = 7.7, 1.3 Hz, 1H), 7.48 (td, J = 7.6, 1.1 Hz, 1H), 7.30 (d, J = 7.3 Hz, 1H), 6.94 (d, J = 9.0 Hz, 2H), 6.89 (d, J = 9.0 Hz, 2H), 3.82 (s, 3H), 2.66 (s, 3H), 2.19 (s, 3H).

^{13}C NMR (101 MHz, $CDCl_3$) δ 187.1, 158.3, 153.9, 147.9, 135.4, 130.5, 127.6, 123.5, 123.1, 119.7, 58.4, 56.7, 34.6, 22.5, 18.5.

*2,3-Dimethyl-3-(4-trifluoromethylphenyl)-3H-indole (**11d**)*

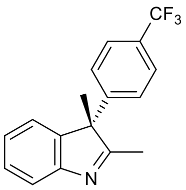

The title compound was prepared according to the general protocol **G** using 0.81 mmol (0.17 g) of **10d** and 0.77 mmol (0.11 g) of phenylhydrazine hydrochloride in 25 mL of dry EtOH. The product was yielded as an orange oil (33 mg; 15%).

^{1}H NMR (400 MHz, $CDCl_3$) δ 8.07 (d, J = 7.9 Hz, 2H), 7.68 (d, J = 8.2 Hz, 2H), 7.63–7.56 (m, 1H), 7.56–7.49 (m, 2H), 7.46–7.37 (m, 1H), 2.71 (s, 3H), 1.94 (s, 3H).

HRMS (ESI+): calculated for $[C_{17}H_{15}F_3N + H]^+$: 290.1151; found: 290.1153.

*2,3-Dimethyl-3-(4-nitrophenyl)-3H-indole (**11e**)*

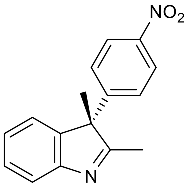

The title compound was prepared according to the general protocol **G** using 0.71 mmol (0.14 g) of **10e** and 0.68 mmol (98 mg) of phenylhydrazine hydrochloride in 25 mL of dry EtOH. The product was yielded as an orange oil (92 mg; 51%).

^{1}H NMR (400 MHz, $CDCl_3$) δ 8.24 (d, J = 8.9 Hz, 2H), 8.03 (d, J = 7.9 Hz, 1H), 7.58 (td, J = 7.8, 1.2 Hz, 1H), 7.46–7.56 (m, 1H), 7.45 (d, J = 8.9 Hz, 1H), 7.23 (d, J = 8.9 Hz, 2H), 2.16 (s, 3H), 1.87 (s, 3H).

HRMS (ESI+): calculated for $[C_{16}H_{15}N_2O_2 + H]^+$: 267.1128; found: 267.1126.

<u>*Indolium salts **12a-e***</u>

*General protocol **H***

A solution of 3*H*-indole **11** (1.00 eq.) and iodomethane (40 – 50 eq.) in dry acetonitrile was stirred at 55 °C under argon atmosphere for 24 h. The reaction mixture was cooled down to 0 °C. Cold Et_2O

(30 mL) was added dropwise. Precipitated solids were filtered, washed with Et_2O, dried in vacuo and used in the next reaction step without further purification.

*1,2,3-Trimethyl-3-phenyl-3H-indolium iodide (**12a**)*

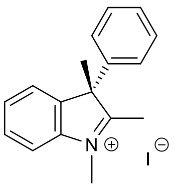

The title compound was prepared according to the general protocol **H** using 0.52 mmol (0.12 g) of **11a** and 20.8 mmol (1.3 mL) of iodomethane in 6 mL of dry acetonitrile. The product was yielded as a light yellow solid (0.13 g; 82%) and used in the next reaction step without further purification.

^{1}H NMR (400 MHz, $CDCl_3$) *δ* 7.70 (d, *J* = 8.0 Hz, 1H), 7.58 (dd, *J* = 7.8; 1.2 Hz, 1H), 7.52 – 7.48 (m, 1H), 7.25–7.36 (m, 4H), 6.95–7.05 (m, 2H), 4.35 (s, 3H), 2.87 (s, 3H), 2.06 (s, 3H).

The NMR data is in agreement with ref. [S16].

*1,2,3-Trimethyl-3-(4-methylphenyl)-3H-indolium iodide (**12b**)*

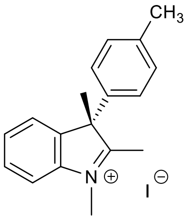

The title compound was prepared according to the general protocol **H** using 0.43 mmol (0.10 g) of **11b** and 17.1 mmol (1.1 mL) of iodomethane in 4 mL of dry acetonitrile. The product was yielded as a light brown solid (0.12 g; 63%) and used in the next reaction step without further purification.

^{1}H NMR (400 MHz, $CDCl_3$) *δ* 7.69 (d, *J* = 8.1 Hz, 1H), 7.59 (dd, *J* = 7.7; 1.2 Hz, 1H), 7.51 (dd, *J* = 7.5; 1.2 Hz, 1H), 7.29 (d, *J* = 7.5 Hz, 1H), 7.12 (d, *J* = 7.8 Hz, 2H), 6.87 (d, *J* = 7.9 Hz, 2H) 4.31 (s, 3H), 2.80 (s, 3H), 2.28 (s, 3H), 2.01 (s, 3H).

*1,2,3-Trimethyl-3-(4-methoxyphenyl)-3H-indolium iodide (**12c**)*

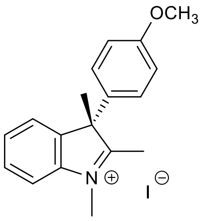

The title compound was prepared according to the general protocol **H** using 0.53 mmol (0.13 g) of **11c** and 26.4 mmol (1.7 mL) of iodomethane in 6 mL of dry acetonitrile. The product was yielded as a dark red solid (0.12 g; 63%) and used in the next reaction step without further purification.

^{1}H NMR (400 MHz, $CDCl_3$) *δ* 7.79 (dd, *J* = 8.0, 1.3 Hz, 1H), 7.69 – 7.63 (m, 1H), 7.59 (td, *J* = 7.6, 1.0 Hz, 1H), 7.37 (dd, *J* = 8.0, 1.2 Hz, 1H), 7.00 (d, *J* = 8.8 Hz, 2H), 6.90 (d, *J* = 8.8 Hz, 2H), 4.34 (s, 3H), 3.80 (s, 3H), 2.84 (s, 3H), 2.01 (s, 3H).

*1,2,3-Trimethyl-3-(4-trifluoromethylphenyl)-3H-indolium iodide (**12d**)*

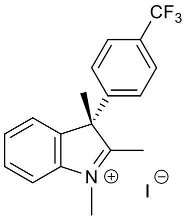

The title compound was prepared according to the general protocol **H** using 0.11 mmol (33 mg) of **11d** and 5.65 mmol (0.35 mL) of iodomethane in 4 mL of dry acetonitrile. The product was yielded as a dark red solid (45 mg; 93%) and used in the next reaction step without further purification.

^{1}H NMR (400 MHz, $CDCl_3$) *δ* 7.82 (d, *J* = 7.8 Hz, 2H), 7.63 (d, *J* = 7.8 Hz, 2H), 7.59 – 7.48 (m, 2H), 7.09 (td, *J* = 7.4, 1.0 Hz, 1H), 7.01 (d, *J* = 8.0 Hz, 1H), 4.42 (s, 3H), 2.92 (s, 3H), 1.89 (s, 3H).

*1,2,3-Trimethyl-3-(4-nitrophenyl)-3H-indolium iodide (**12e**)*

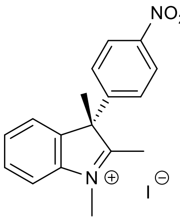

The title compound was prepared according to the general protocol **H** using 0.35 mmol (92 mg) of **11e** and 17.3 mmol (1.1 mL) of iodomethane in 4 mL of dry acetonitrile. The product was yielded as a brown solid (103 mg; 73%) and used in the next reaction step without further purification.

^{1}H NMR (400 MHz, $CDCl_3$) δ 8.27 (d, J = 8.1 Hz, 2H), 7.82 (d, J = 8.0 Hz, 1H), 7.73 (t, J = 7.6 Hz 1H), 7.63 (t, J = 7.5 Hz, 1H), 7.40 (d, J = 8.1 Hz, 2H), 7.35 (d, J = 7.5 Hz, 1H), 4.42 (s, 3H), 2.17 (s, 6H).

<u>*Spiropyrans **IIa-e***</u>

*1',3'-Dimethyl-3'-phenyl-6-nitrospiro[chromene-2,2'-indoline] (**IIa**)*

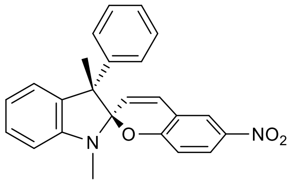

A solution of indolium salt **12a** (118 mg; 0.32 mmol), 2-hydroxy-5-nitrobenzaldehyde (54 mg; 0.32 mmol) and triethylamine (91 µl; 0.65 mmol) in dry ethanol (20 mL) was refluxed under argon atmosphere for 2 h. The reaction mixture was cooled down to room temperature and ethanol was removed under reduced pressure. The crude product was dissolved in DCM (25 mL). The organic layer was washed with distilled water (4 × 20 mL) and then dried over anhydrous $MgSO_4$. The crude product was purified by column chromatography on alumina using hexane:DCM, 1:1 mixture as eluent. The compound **IIa** was obtained as a purple solid (77 mg; 61%).

^{1}H NMR (400 MHz, $CDCl_3$, diastereomeric ratio *syn:anti* = 51:49) δ 8.06 (dd, J = 9.0, 2.8 Hz, 1H), 8.00 (d, J = 2.8 Hz, 1H), 7.73 (d, J = 2.8 Hz, 1H), 7.66 (dd, J = 9.0, 2.8 Hz, 1H), 7.40 (d, J = 7.1 Hz, 1H), 7.36 – 7.23 (m, 6H), 7.12 (dd, J = 7.4, 1.3 Hz, 1H), 7.07 – 6.90 (m, 8H), 6.85 (t, J = 9.6 Hz, 2H), 6.73 – 6.67 (m, 3H), 6.18 (d, J = 9.0 Hz, 1H), 5.98 (d, J = 10.3 Hz, 1H), 4.93 (d, J = 10.3 Hz, 1H), 2.88 (s, 3H), 2.71 (s, 3H), 1.77 (s, 3H), 1.60 (s, 3H).

^{13}C NMR (101 MHz, $CDCl_3$) δ 159.5, 159.0, 149.4, 148.8, 141.78, 141.1, 140.4, 139.3, 135.4, 134.2, 129.0, 128.8, 128.6, 128.2, 128.1, 128.0, 127.5, 127.2, 126.9, 126.9, 125.8, 125.1, 124.6, 123.4, 123.1, 122.7, 122.3, 121.9, 120.3, 119.5, 118.7, 118.7, 115.5, 115.4, 107.5, 107.1, 106.6, 106.1, 60.3, 59.8, 29.3, 28.9, 23.4, 19.6.

HRMS (ESI+): calculated for $[C_{24}H_{20}N_2O_3 + H]^+$: 385.1547; found: 385.1552.

*1',3'-Dimethyl-3'-(4-methylphenyl)-6-nitrospiro[chromene-2,2'-indoline] (**IIb**)*

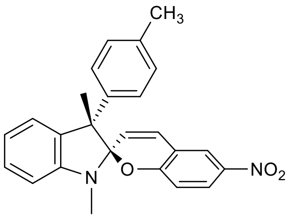

A solution of indolium salt **12b** (65 mg; 0.17 mmol), 2-hydroxy-5-nitrobenzaldehyde (29 mg; 0.17 mmol) and triethylamine (48 µl; 0.34 mmol) in dry ethanol (30 mL) was refluxed under argon atmosphere for 2 h. The reaction mixture was cooled down to room temperature and ethanol was removed under reduced pressure. The crude product was dissolved in DCM (40 mL). The organic layer was washed with distilled water (3 × 20 mL) and then dried over anhydrous $MgSO_4$. The crude product was purified by column chromatography on alumina using hexane:DCM, 1:1 mixture as eluent. The compound **IIb** was obtained as a dark purple solid (46 mg; 67%).

^{1}H NMR (400 MHz, $CDCl_3$, diastereomeric ratio *syn:anti* = 52:48) δ 8.05 (dd, J = 9.0, 2.8 Hz, 1H), 7.99 (d, J = 2.8 Hz, 1H), 7.72 (d, J = 2.8 Hz, 1H), 7.67 (dd, J = 9.0, 2.8 Hz, 1H), 7.35 – 7.22 (m, 4H), 7.10 (dd, J = 7.3, 1.3 Hz, 1H) 7.08 – 7.01 (m, 3H), 6.96 – 6.89 (m, 2H), 6.88 – 6.80 (m, 6H), 6.71 – 6.66 (m, 3H),

6.18 (dd, J = 8.9, 0.6 Hz, 1H), 5.97 (d, J = 10.3 Hz, 1H), 4.95 (d, J = 10.4 Hz, 1H), 2.86 (s, 3H), 2.70 (s, 3H), 2.31 (s, 3H), 2.12 (s, 3H), 1.74 (s, 3H), 1.57 (s, 3H).

^{13}C NMR (101 MHz, $CDCl_3$) δ 159.6, 159.1, 149.5, 148.8, 141.1, 140.5, 136.9, 136.7, 136.3, 135.7, 134.4, 131.8, 129.8, 129.0, 128.8, 128.5, 128.3, 128.1, 128.0, 126.8, 125.8, 124.9, 124.7, 123.4, 123.3, 122.8, 122.5, 122.0, 120.3, 119.6, 118.9, 118.7, 115.7, 115.5, 107.5, 107.1, 106.7, 106.2, 56.0, 59.5, 29.4, 28.9, 23.5, 21.0, 20.8, 19.7.

HRMS (APCI+): calculated for $[C_{25}H_{22}N_2O_3 + H]^+$: 399.1703; found: 399.1699.

*1',3'-Dimethyl-3'-(4-methoxyphenyl)-6-nitrospiro[chromene-2,2'-indoline] (**IIc**)*

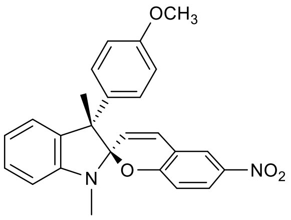

A solution of indolium salt **12c** (190 mg; 0.48 mmol), 2-hydroxy-5-nitrobenzaldehyde (81 mg; 0.48 mmol) and triethylamine (135 µl; 0.97 mmol) in dry ethanol (40 mL) was refluxed under argon atmosphere for 2 h. The reaction mixture was cooled down to room temperature and ethanol was removed under reduced pressure. The crude product was dissolved in DCM (40 mL). The organic layer was washed with distilled water (3 × 30 mL) and then dried over anhydrous $MgSO_4$. The crude product was purified by column chromatography on alumina using hexane:DCM, 1:1 mixture as eluent. The compound **IIc** was obtained as a dark red oil (96 mg; 48%).

^{1}H NMR (400 MHz, $CDCl_3$, diastereomeric ratio *syn:anti* = 48:52) δ 8.01 (dd, J = 9.1, 2.8 Hz, 1H), 7.96 (d, J = 2.8 Hz, 1H), 7.70 (d, J = 2.8 Hz, 1H), 7.66 (dd, J = 9.0, 2.6 Hz, 1H), 7.31 – 7.21 (m, 2H), 7.18 – 6.98 (m, 5H), 6.93 – 6.78 (m, 7H), 6.77 – 6.61 (m, 4H), 6.55 (d, J = 8.7 Hz, 1H), 6.18 (d, J = 8.9 Hz, 1H), 5.92 (d, J = 10.3 Hz, 1H), 4.92 (d, J = 10.4 Hz, 1H), 3.78 (s, 3H), 3.59 (s, 3H), 2.83 (s, 3H), 2.66 (s, 3H), 1.70 (s, 3H), 1.52 (s, 3H).

^{13}C NMR (101 MHz, $CDCl_3$) δ 159.7, 159.1, 158.7, 158.5, 149.4, 148.8, 141.1, 140.5, 135.7, 134.6, 133.9, 130.1, 129.2, 128.9, 128.6, 128.2, 127.1, 126.9, 125.8, 125.1, 124.6, 123.4, 123.3, 122.8, 122.49, 122.0, 120.7, 120.4, 119.6, 115.7, 115.5, 114.2, 113.5, 113.0, 107.5, 107.2, 106.7, 106.4, 59.7, 59.2, 55.3, 55.3, 29.4, 29.0, 25.3, 19.8.

HRMS (APCI+): calculated for $[C_{25}H_{22}N_2O_4 + H]^+$: 415.1652; found: 415.1655.

*1',3'-Dimethyl-3'-(4-trifluoromethylphenyl)-6-nitrospiro[chromene-2,2'-indoline] (**IId**)*

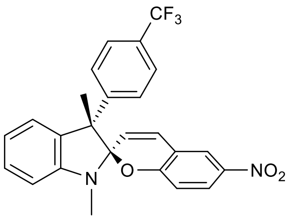

A solution of indolium salt **12d** (45 mg; 0.11 mmol), 2-hydroxy-5-nitrobenzaldehyde (18 mg; 0.11 mmol) and triethylamine (29 µl; 0.21 mmol) in dry ethanol (15 mL) was refluxed under argon atmosphere for 2 h. The reaction mixture was cooled down to room temperature and ethanol was removed under reduced pressure. The crude product was dissolved in DCM (30 mL). The organic layer was washed with distilled water (3 × 20 mL) and then dried over anhydrous $MgSO_4$. The crude product was purified by column chromatography on alumina using hexane:DCM, 1:1 mixture as eluent. The compound **IId** was obtained as a dark yellow solid (20 mg; 42%).

^{1}H NMR (400 MHz, $CDCl_3$, diastereomeric ratio *syn:anti* = 49:51) δ 8.07 (dd, J = 9.0, 2.7 Hz, 1H), 8.01 (d, J = 2.7 Hz, 1H), 7.74 (d, J = 2.7 Hz, 1H), 7.68 (dd, J = 9.0, 2.7 Hz, 1H), 7.52 (d, J = 8.1 Hz, 2H), 7.49 (d, J = 8.2 Hz, 2H), 7.37 – 7.29 (m, 4H), 7.11 – 7.05 (m, 3H), 7.01 (dd, J = 7.3, 1.0 Hz, 1H), 6.95 (td, J = 7.4, 1.0 Hz, 1H), 6.94 (td, J = 7.5, 1.0 Hz, 1H), 6.86 (d, J = 10.3 Hz, 1H), 6.85 (d, J = 10.3 Hz, 1H), 6.75 – 6.68

(m, 3H), 6.16 (d, *J* = 9.0 Hz, 1H), 5.98 (d, *J* = 10.3 Hz, 1H), 4.87 (d, *J* = 10.3 Hz, 1H), 2.85 (s, 3H), 2.71 (s, 3H), 1.76 (s, 3H), 1.61 (s, 3H).

^{13}C NMR (101 MHz, $CDCl_3$) δ 159.4, 158.6, 149.4, 148.7, 146.0, 144.1, 141.3, 140.8, 134.8, 133.4, 129.3, 129.0, 128.6, 128.6, 127.6, 127.3, 126.0, 125.3, 125.2, 125.1, 124.5, 124.4, 123.4, 122.9, 122.6, 122.1, 122.0, 120.6, 119.8, 118.6, 118.5, 116.5, 115.6, 115.5, 107.8, 107.5, 106.4, 105.7, 60.3, 59.9, 29.2, 29.0, 23.4, 19.6.

HRMS (APCI+): calculated for $[C_{25}H_{19}F_3N_2O_3 + H]^+$: 453.1421; found: 453.1424.

*1',3'-Dimethyl-3'-(4-nitromethylphenyl)-6-nitrospiro[chromene-2,2'-indoline] (**IIe**)*

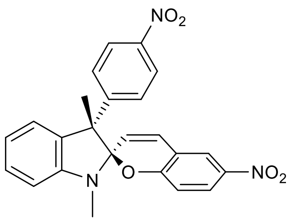

A solution of indolium salt **12e** (103 mg; 0.25 mmol), 2-hydroxy-5-nitrobenzaldehyde (42 mg; 0.125mmol) and triethylamine (71 µl; 0.51 mmol) in dry ethanol (30 mL) was refluxed under argon atmosphere for 2 h. The reaction mixture was cooled down to room temperature and ethanol was removed under reduced pressure. The crude product was dissolved in DCM (40 mL). The organic layer was washed with distilled water (3 × 20 mL) and brine (20 mL) and then dried over anhydrous $MgSO_4$. The crude product was purified by column chromatography on alumina using hexane:DCM, 1:1 mixture as eluent. The compound **IIe** was obtained as a yellow-green solid (60 mg; 55%).

^{1}H NMR (400 MHz, $CDCl_3$, diastereomeric ratio *syn:anti* = 49:51) δ 8.09 – 8.05 (m, 3H), 8.01 (d, *J* = 2.7 Hz, 1H), 7.95 (d, *J* = 8.9 Hz, 2H), 7.78 (d, *J* = 2.7 Hz, 1H), 7.71 (dd, *J* = 9.0, 2.7 Hz, 1H), 7.60 (d, *J* = 8.9 Hz, 2H), 7.39 – 7.29 (m, 3H), 7.15 (d, *J* = 8.5 Hz, 1H), 7.06 – 6.99 (m, 2H), 6.99 – 6.93 (m, 2H), 6.90 (d, *J* = 10.3 Hz, 1H), 6.87 (d, *J* = 8.9 Hz, 1H), 6.77 – 6.69 (m, 3H), 6.24 (d, *J* = 9.0 Hz, 1H), 6.01 (d, *J* = 10.3 Hz, 1H), 4.86 (d, *J* = 10.3 Hz, 1H), 2.84 (s, 3H), 2.73 (s, 3H), 1.78 (s, 3H), 1.63 (s, 3H).

^{13}C NMR (101 MHz, $CDCl_3$) δ 159.3, 158.4, 149.5, 149.4, 148.5, 147.6, 146.9, 146.9, 141.4, 141.0, 134.3, 133.1, 130.0, 129.5, 129.3, 128.8, 128.0, 126.1, 125.6, 124.3, 123.4, 123.3, 123.0, 122.7, 122.5, 122.2, 121.2, 120.8, 120.0, 118.5, 118.2, 115.6, 115.3, 108.0, 107.7, 106.2, 105.4, 60.5, 60.2, 29.1, 29.1, 23.8, 19.6.

HRMS (APCI+): calculated for $[C_{24}H_{19}N_3O_5 + H]^+$: 430.1398; found: 430.1398.

2.3 Synthesis of the Series III

*3-Tert-butyl-2-hydroxy-5-nitrobenzaldehyde (**14a**)*

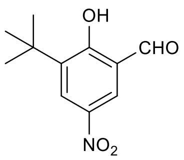

A stirred solution of 3-*tert*-butyl-2-hydroxybenzaldehyde (**13**; 1.12 mmol, 2.00 g) in acetic acid (4 mL) was cooled down to 0 °C. Fuming nitric acid (27.2 mmol, 1.12 mL) was cautiously added dropwise over 15 min. The cooling bath was removed, and the reaction mixture was stirred for an additional hour at room temperature. The reaction mixture was poured on ice/water mixture (250 mL) under stirring, while pale orange crystals precipitated out. The ice was let melt and then the mixture was filtered on a frit. The solids were washed with additional 50 mL of water and recrystallised from $EtOH/H_2O$ mixture. Yellow crystals (0.48 g, 20%) were obtained.

^{1}H NMR (400 MHz, $CDCl_3$) δ 12.43 (s, 1H), 9.96 (s, 1H), 8.40 (d, *J* = 2.7 Hz, 1H), 8.38 (d, *J* = 2.7 Hz, 1H), 1.43 (s, 9H).

^{13}C NMR (101 MHz, $CDCl_3$) δ 196.4, 165.9, 140.7, 140.1, 128.8, 128.0, 119.4, 35.5, 28.9.

The NMR data is in agreement with ref. [S17].

*3-Bromo-2-hydroxy-5-nitrobenzaldehyde (**15**)*

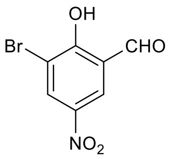

A solution of 2-hydroxy-5-nitrobenzaldehyde (24.0 mmol, 4.0 g) and *N*-bromosuccinimide (24.0 mmol, 4.3 g) in dry acetonitrile (120 mL) was stirred at room temperature for 20 h under argon atmosphere. The solvent was evaporated, the crude residue was dissolved in 120 mL of ethyl acetate and 200 mL of brine was added. Phases were separated, and the organic phase was washed with additional 200 mL of water, dried over $MgSO_4$ and concentrated under reduced pressure. The crude product was recrystallized from EtOH/H_2O mixture, yielding 4.8 g (81%) of yellow crystals.

^{1}H NMR (400 MHz, $CDCl_3$) δ 12.24 (s, 1H), 9.97 (s, 1H), 8.69 (d, *J* = 2.6 Hz, 1H), 8.54 (d, *J* = 2.6 Hz, 1H).

^{13}C NMR (101 MHz, $CDCl_3$) δ 195.1, 163.0, 140.7, 134.6, 128.4, 119.5, 112.4.

The NMR data is in agreement with ref. [S17].

<u>*Hydroxy aldehydes **14b-f***</u>

*General protocol **J***

Na_2CO_3 (3.0 eq.) and $(PPh_3)_4Pd(0)$ (2 mol%) were added to a solution of bromo aldehyde **15** (1.00 eq.) and corresponding arylboronic acid (1.50 eq.) in 1,4-dioxane:H_2O, 9:1 mixture (c = 0.2 mol/L). The resulting suspension was heated to 100 °C and stirred for 24 hours. After cooling to room temperature, water (5 mL) was added, followed by a dropwise addition of HCl (10%), up to pH = 2. The solution was extracted with 3 × 20 mL of dichloromethane, dried over $MgSO_4$ and concentrated under reduced pressure.

*2-Hydroxy-5-nitro-3-phenylbenzaldehyde (**14b**)*

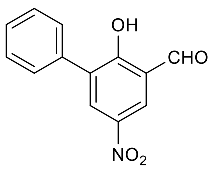

The title compound was prepared according to the general protocol **J** using 0.41 mmol (0.10 g) of **15**, 0.61 mmol (0.07 g) of phenylboronic acid, 1.22 mmol (0.13 g) of Na_2CO_3 and 0.01 mmol (0.01 g) of $(PPh_3)_4Pd(0)$. The crude product was purified via column chromatography (silica gel) using toluene as eluent. The product was yielded as a light-yellow solid (44 mg; 45%).

^{1}H NMR (400 MHz, $CDCl_3$) δ 12.16 (s, 1H), 10.04 (s, 1H), 8.54 (d, *J* = 2.8 Hz, 1H), 8.49 (d, *J* = 2.8 Hz, 1H), 7.62 – 7.41 (m, 5H).

^{13}C NMR (101 MHz, $CDCl_3$) δ 196.0, 163.7, 140.7, 134.1, 132.4, 131.9, 129.3, 129.0, 128.7, 128.6, 119.8.

The NMR data is in agreement with ref. [S17].

*2-Hydroxy-5-nitro-3-(4-fluorophenyl)benzaldehyde (**14c**)*

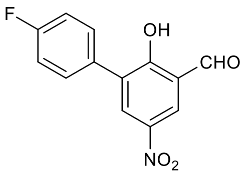

The title compound was prepared according to the general protocol **J** using 2.00 mmol (0.49 g) of **15**, 3.00 mmol (0.42 g) of 4-fluorophenylboronic acid, 6.00 mmol (0.64 g) of Na_2CO_3 and 0.04 mmol (0.05 g) of $(PPh_3)_4Pd(0)$. The crude product was purified via column chromatography (silica gel) using toluene:MTBE, 30:1 as eluent. The product was yielded as a white solid (180 mg; 35%).

.^{1}H NMR (400 MHz, $CDCl_3$) δ 12.19 (s, 1H), 10.04 (s, 1H), 8.54 (d, *J* = 2.7 Hz, 1H), 8.46 (d, *J* = 2.7 Hz, 1H), 7.59 (dd, *J* = 8.9, 5.3 Hz, 2H), 7.17 (dd, *J* = 8.9, 8.5 Hz, 2H).

^{13}C NMR [^{19}F] (101 MHz, $CDCl_3$) *δ* 196.0, 163.5, 140.6, 131.7, 131.3, 131.2, 131.1, 128.6, 119.8, 115.9, 115.7.

^{19}F NMR (376 MHz, $CDCl_3$) *δ* –112,14 (tt, *J* = 8.5, 5.3 Hz, 1F).

*2-Hydroxy-5-nitro-3-(4-tert-butylphenyl)benzaldehyde (**14d**)*

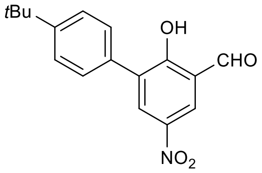

The title compound was prepared according to the general protocol **J** using 1.02 mmol (0.25 g) of **15**, 1.52 mmol (0.27 g) of 4-*tert*-butylphenylboronic acid, 3.05 mmol (0.32 g) of Na_2CO_3 and 0.02 mmol (0.02 g) of $(PPh_3)_4Pd(0)$. The crude product was purified via column chromatography (silica gel) using toluene:MTBE, 30:1 as eluent. The product was yielded as a light-yellow solid (130 mg; 41%).

^{1}H NMR (400 MHz, $CDCl_3$) *δ* 12.18 (s, 1H), 10.03 (s, 1H), 8.52 (d, *J* = 2.8 Hz, 1H), 8.49 (d, *J* = 2.8 Hz, 1H), 7.56 (d, *J* = 8.9 Hz, 2H), 7.51 (d, *J* = 8.9 Hz, 2H), 1.37 (s, 9H).

^{13}C NMR (101 MHz, $CDCl_3$) *δ* 196.0, 163.7, 152.1, 140.7, 132.3, 131.8, 131.2, 129.0, 128.4, 125.7, 119.8, 34.9, 31.4.

*2-Hydroxy-5-nitro-3-naphtalen-1-ylbenzaldehyde (**14e**)*

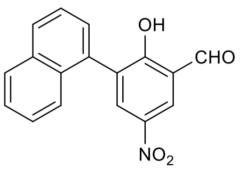

The title compound was prepared according to the general protocol **J** using 1.22 mmol (0.30 g) of **15**, 1.83 mmol (0.32 g) of naphthalen-1-ylboronic acid, 3.66 mmol (0.39 g) of Na_2CO_3 and 0.02 mmol (0.03 g) of $(PPh_3)_4Pd(0)$. The crude product was purified via column chromatography (silica gel) using DCM:hexane, 5:1 as eluent. The product was yielded as a light-yellow solid (41 mg; 12%).

^{1}H NMR (400 MHz, $CDCl_3$) *δ* 11.89 (s, 1H), 10.09 (s, 1H), 8.66 (dd, *J* = 2.7, 0.8 Hz, 1H), 8.49 (dd, *J* = 2.8, 0.6 Hz, 1H), 7.95 (ddd, *J* = 11.3, 8.2, 1.2 Hz, 1H), 7.57 (td, *J* = 7.0, 0.8 Hz, 1H), 7.55 – 7.49 (m 2H), 7.49 – 7.42 (m, 3H).

^{13}C NMR (101 MHz, $CDCl_3$) *δ* 195.8, 164.2, 140.5, 133.6, 133.4, 132.1, 131.7, 131.3, 129.6, 129.2, 128.7, 127.9, 126.8, 126.4, 125.4, 125.2, 119.6.

*2-Hydroxy-5-nitro-3-naphtalen-2-ylbenzaldehyde (**14f**)*

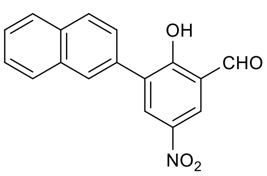

The title compound was prepared according to the general protocol **J** using 1.22 mmol (0.30 g) of **15**, 1.83 mmol (0.32 g) of naphthalen-1-ylboronic acid, 3.66 mmol (0.39 g) of Na_2CO_3 and 0.02 mmol (0.03 g) of $(PPh_3)_4Pd(0)$. The crude product was purified via column chromatography (silica gel) using DCM:hexane, 3:1 as eluent and subsequent recrystallisation from toluene. The product was yielded as a light-yellow solid (43 mg; 12%).

^{1}H NMR (400 MHz, acetone-*d*6) *δ* 12.38 (s, 1H), 10.30 (s, 1H), 8.85 (d, *J* = 2.8 Hz, 1H), 8.61 (d, *J* = 2.7 Hz, 1H), 8.25 (d, *J* = 1.8 Hz, 1H), 8.06 – 7.96 (m, 3H), 7.84 (dd, *J* = 8.6, 1.8 Hz, 1H), 7.62 – 7.56 (m 2H).

^{13}C NMR (101 MHz, acetone-*d*6) *δ* 205.4, 163.1, 134.0, 133.4, 133.2, 132.3, 131.5, 129.3, 128.8, 128.4, 128.0, 127.7, 127.0, 126.8, 126.7, 126.5, 126.3.

*Spiropyrans **IIIa-f***

*1',3'-Dimethyl-3'-benzyl-6-nitro-8-tert-butylspiro[chromene-2,2'-indoline] (**IIIa**)*

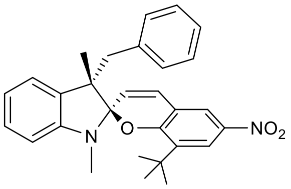

A solution of indolium salt **6a** (0.40 mmol, 151 mg), hydroxy aldehyde **14a** (0.40 mmol, 89 mg) and triethylamine (0.80 mmol, 112 µL) in dry ethanol (15 mL) was heated to reflux and stirred under argon atmosphere in darkness (covered with aluminium foil) for 2 hours. The solvent was evaporated, and the residue dissolved in Et_2O (40 mL). The solution was washed with water (3 × 30 mL), dried over $MgSO_4$ and concentrated under reduced pressure. The crude residue was dissolved in Et_2O (30 mL). 1M Ethereal HCl (ca 2 mL) was added dropwise under stirring, while orange crystals precipitated. The solution was stirred for 15 minutes, then filtered and washed with additional 20 mL of Et_2O. The obtained solids were dissolved in DCM (100 mL). The organic solution was washed with aq. $NaHCO_3$ (3 × 100 mL), dried over $MgSO_4$ and concentrated under reduced pressure. 148 mg (78%) of deep purple crystals were obtained.

^1H NMR (400 MHz, $CDCl_3$, diastereomeric ratio *syn:anti* = 27:73) δ 8.15 (d, J = 2.8 Hz, 1H), 8.06 (d, J = 2.7 Hz, 1H), 7.92 (d, J = 2.7 Hz, 1H), 7.89 (d, J = 2.7 Hz, 1H), 7.22 – 7.08 (m, 9H), 7.05 – 6.99 (m, 2H), 6.88 (td, J = 7.5, 1.0 Hz, 1H), 6.69 – 6.62 (m, 5H), 6.58 (d, J = 7.2 Hz, 1H), 6.47 (d, J = 7.1 Hz, 1H), 6.19 (dd, J = 7.3, 1.2 Hz, 1H), 6.07 (d, J = 10.4 Hz, 1H), 5.58 (d, J = 10.3 Hz, 1H), 3.40 (d, J = 13.5 Hz, 1H), 3.17 (d, J = 13.6 Hz, 1H), 2.81 (d, J = 12.0 Hz, 1H), 2.77 (d, J = 12.0 Hz, 1H), 2.76 (s, 3H), 2.54 (s, 3H), 1.30 (s, 3H), 1.24 (s, 9H), 1.22 (s, 3H), 1.05 (s, 9H).

^{13}C NMR (101 MHz, $CDCl_3$) δ 158.5, 157.6, 148.3, 146.8, 140.8, 140.4, 137.8, 137.5, 137.5, 137.0, 136.0, 132.2, 131.7, 131.0, 130.1, 128.1, 128.1, 128.0, 127.7, 127.3, 126.5, 126.4, 124.8, 123.7, 123.5, 121.7, 121.3, 121.2, 119.8, 119.7, 119.6, 119.2, 118.8, 108.7, 107.2, 107.1, 105.1, 56.7, 53.9, 41.8, 39.0, 35.0, 34.9, 29.6, 29.5, 29.3, 28.0, 23.9, 16.1.

HRMS (APCI+): calculated for $[C_{29}H_{30}N_2O_3 + H]^+$: 455.2329; found: 455.2332.

*1',3'-Dimethyl-3'-benzyl-6-nitro-8-phenylspiro[chromene-2,2'-indoline] (**IIIb**)*

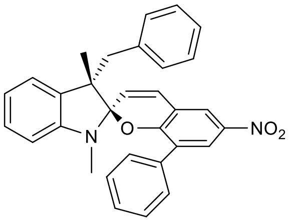

A solution of indolium salt **6a** (0.19 mmol, 72 mg), hydroxy aldehyde **14b** (0.19 mmol, 46 mg) and triethylamine (0.37 mmol, 52 µL) in dry ethanol (15 mL) was heated to reflux and stirred under argon atmosphere in darkness (covered with aluminium foil) for 2 hours. The solvent was evaporated, and the residue dissolved in Et_2O (30 mL). The solution was washed with water (3 × 20 mL), dried over $MgSO_4$ and concentrated under reduced pressure. The crude residue was dissolved in Et_2O (10 mL). 1M Ethereal HCl (ca 1 mL) was added dropwise under stirring, while orange crystals precipitated. The solution was stirred for 15 minutes, then filtered and washed with additional 15 mL of Et_2O. The obtained solids were dissolved in DCM (80 mL). The organic solution was washed with aq. $NaHCO_3$ (3 × 100 mL), dried over $MgSO_4$ and concentrated under reduced pressure. 72 mg (80%) of deep purple crystals were obtained.

^1H NMR (400 MHz, $CDCl_3$, diastereomeric ratio *syn:anti* = 30:70) δ 8.25 (d, J = 2.7 Hz, 1H), 8.21 (d, J = 2.8 Hz, 1H), 8.02 (d, J = 2.8 Hz, 1H), 7.97 (d, J = 2.7 Hz, 1H), 7.36 – 7.31 (m, 2H), 7.25 – 6.95 (m, 16H), 6.90 – 6.82 (m, 3H), 6.70 – 6.54 (m, 8H), 6.12 (d, J = 10.3 Hz, 1H), 6.09 (dd, J = 7.3, 1.2 Hz, 1H), 5.63 (d, J = 10.3 Hz, 1H), 3.11 (d, J = 13.0 Hz, 1H), 3.00 (d, J = 12.9 Hz, 1H), 2.82 (d, J = 12.3 Hz, 1H), 2.79 – 2.73 (m, 4H), 2.64 (s, 3H), 1.30 (s, 3H), 1.10 (s, 3H).

^{13}C NMR (101 MHz, $CDCl_3$) δ 156.5, 155.5, 148.2, 146.8, 141.3, 141.1, 137.6, 136.9, 136.3, 132.4, 131.6, 130.9, 129.9, 129.7, 129.6, 129.1, 128.9, 128.4, 128.1, 128.1, 128.0, 127.9, 127.8, 127.6, 127.6, 127.4, 126.5, 126.4, 126.4, 126.2, 126.1, 124.8, 123.4, 122.2, 122.0, 121.7, 121.6, 120.8, 120.0, 119.6, 119.0, 108.3, 107.2, 107.2, 104.6, 56.6, 53.9, 41.4, 39.4, 29.6, 28.3, 22.8, 16.5.

HRMS (APCI+): calculated for $[C_{31}H_{26}N_2O_3 + H]^+$: 475.2016; found: 475.2022.

*1',3'-Dimethyl-3'-benzyl-6-nitro-8-(4-fluorophenyl)spiro[chromene-2,2'-indoline] (**IIIc**)*

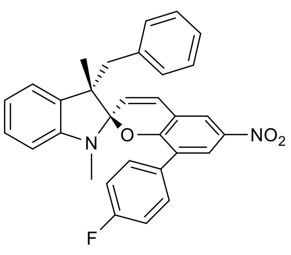

A solution of indolium salt **6a** (0.27 mmol, 102 mg), hydroxy aldehyde **14c** (0.27 mmol, 71 mg) and triethylamine (0.53 mmol, 74 µL) in dry ethanol (5 mL) was heated to reflux and stirred under argon atmosphere in darkness (covered with aluminium foil) for 2 hours. The solvent was evaporated, and the residue dissolved in Et_2O (20 mL). The solution was washed with water (3 × 30 mL), dried over $MgSO_4$ and concentrated under reduced pressure. The crude residue was dissolved in Et_2O:EtOAC, 1:1 mixture (50 mL). 1M Ethereal HCl (ca 2 mL) was added dropwise under stirring, while orange crystals precipitated. The solution was stirred for 15 minutes, then filtered and washed with additional 30 mL of Et_2O. The obtained solids were dissolved in DCM (100 mL). The organic solution was washed with aq. $NaHCO_3$ (3 × 100 mL), dried over $MgSO_4$ and concentrated under reduced pressure. 74 mg (56%) of deep purple crystals were obtained.

^1H NMR (400 MHz, $CDCl_3$, diastereomeric ratio *syn:anti* = 29:71) *δ* 8.21 (d, *J* = 2.7 Hz, 1H), 8.16 (d, *J* = 2.7 Hz, 1H), 8.02 (d, *J* = 2.7 Hz, 1H), 7.97 (d, *J* = 2.7 Hz, 1H), 7.30 (dd, *J* = 9.0, 5.3 Hz, 1H), 7.30 (dt, *J* = 9.9, 5.3 Hz, 1H), 7.26 (dd, *J* = 7.6, 1.3 Hz, 1H), 7.22 – 7.11 (m, 8H), 7.11 – 7.02 (m, 3H), 7.01 (ddd, *J* = 7.6 Hz, 1H), 6.91 – 6.86 (m, 2H), 6.83 – 6.76 (m, 2H), 6.72 – 6.63 (m, 7H), 6.56 (d, *J* = 7.6 Hz, 1H), 6.13 (d, *J* = 10.1 Hz, 1H), 6.11 (ddd, *J* = 7.3, 1.1, 0.6 Hz, 1H), 5.63 (d, *J* = 10.2 Hz, 1H), 2.97 (s, 2H), 2.83 (d, *J* = 12.2 Hz, 1H), 2.77 (s, 3H), 2.76 (d, *J* = 12.2 Hz, 1H), 2.63 (s, 3H), 1.31 (s, 3H), 1.09 (s, 3H).

^{13}C NMR [^{19}F] (101 MHz, $CDCl_3$) *δ* 162.5, 162.3, 156.4, 155.4, 148.1, 146.7, 141.3, 141.1, 137.5, 136.9, 136.2, 132.4, 131.6, 130.9, 130.9, 130.8, 130.7, 130.6, 129.7, 128.0, 127.9, 127.8, 127.6, 127.5, 127.4, 126.5, 126.4, 125.9, 125.8, 124.8, 122.1, 121.7, 121.6, 120.8, 120.4, 120.3, 120.2, 119.7, 119.1, 115.1, 114.9, 108.4, 107.3, 107.2, 104.8, 56.6, 53.9, 41.3, 39.4, 29.6, 28.3, 23.1, 16.5.

HRMS (APCI+): calculated for $[C_{31}H_{25}FN_2O_3 + H]^+$: 493.1922; found: 493.1925.

*1',3'-Dimethyl-3'-benzyl-6-nitro-8-(4-tert-butylphenyl)spiro[chromene-2,2'-indoline] (**IIId**)*

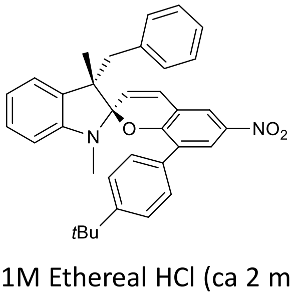

A solution of indolium salt **6a** (0.42 mmol, 158 mg), hydroxy aldehyde **14d** (0.42 mmol, 126 mg) and triethylamine (0.84 mmol, 117 µL) in dry ethanol (15 mL) was heated to reflux and stirred under argon atmosphere in darkness (covered with aluminium foil) for 2 hours. The solvent was evaporated, and the residue dissolved in Et_2O (40 mL). The solution was washed with water (3 × 30 mL), dried over $MgSO_4$ and concentrated under reduced pressure. The crude residue was dissolved in Et_2O (20 mL). 1M Ethereal HCl (ca 2 mL) was added dropwise under stirring, while orange crystals precipitated. The solution was stirred for 20 minutes, then filtered and washed with additional 20 mL of Et_2O. The obtained solids were dissolved in DCM (100 mL). The organic solution was washed with aq. $NaHCO_3$ (3 × 100 mL), dried over $MgSO_4$ and concentrated under reduced pressure. 196 mg (88%) of deep purple crystals were obtained.

^{1}H NMR (400 MHz, $CDCl_3$, diastereomeric ratio *syn:anti* = 30:70) δ 8.27 (d, *J* = 2.7 Hz, 1H), 8.22 (d, *J* = 2.7 Hz, 1H), 8.00 (d, *J* = 2.7 Hz, 1H), 7.95 (d, *J* = 2.7 Hz, 1H), 7.28 (d, *J* = 8.6 Hz, 1H), 7.24 (td, *J* = 7.6, 1.3 Hz, 1H), 7.21 – 6.96 (m, 17H), 6.91 – 6.84 (m, 2H), 6.70 – 6.65 (m, 3H), 6.63 (td, *J* = 7.5, 1.0 Hz, 1H), 6.58 (d, *J* = 7.7 Hz, 1H), 6.55 (dd, *J* = 7.4, 1.0 Hz, 1H), 6.11 (d, *J* = 10.3 Hz, 1H), 6.10 (dd, *J* = 7.4, 1.1 Hz, 1H), 5.62 (d, *J* = 10.3 Hz, 1H), 3.02 (d, *J* = 13.6 Hz, 1H), 2.97 (d, *J* = 13.7 Hz, 1H), 2.84 (d, *J* = 12.2 Hz, 1H), 2.79 – 2.73 (m, 4H), 2.62 (s, 3H), 1.31 (s, 3H), 1.26 (s, 9H), 1.20 (s, 9H), 1.09 (s, 3H).

^{13}C NMR (101 MHz, $CDCl_3$) δ 156.6, 155.6, 151.0, 150.8, 148.3, 146.9, 141.3, 141.1, 137.6, 137.0, 136.2, 132.5, 131.8, 131.7, 131.6, 131.6, 131.0, 129.8, 128.9, 128.7, 128.6, 128.5, 127.9, 127.7, 127.5, 127.4, 126.4, 126.4, 125.9, 125.8, 125.0, 124.8, 124.8, 122.0, 121.4, 121.3, 120.7, 120.3, 120.2, 119.9, 119.5, 118.9, 108.4, 107.4, 107.3, 104.7, 56.6, 53.9, 41.2, 39.4, 34.6, 34.5, 31.3, 31.2, 29.7, 28.3, 23.0, 16.5.

HRMS (APCI+): calculated for $[C_{35}H_{34}N_2O_3 + H]^+$: 531.2642; found: 531.2645.

1',3'-Dimethyl-3'-benzyl-6-nitro-8-(4-tert-butylphenyl)spiro[chromene-2,2'-indoline] ***(IIIe)***

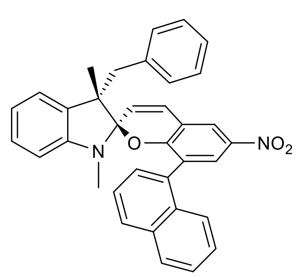

A solution of indolium salt **6a** (0.14 mmol, 53 mg), hydroxy aldehyde **14e** (0.14 mmol, 41 mg) and triethylamine (0.28 mmol, 39 µL) in dry ethanol (5 mL) was heated to reflux and stirred under argon atmosphere in darkness (covered with aluminium foil) for 2 hours. The solvent was evaporated, and the residue dissolved in Et_2O (40 mL). The solution was washed with water (3 × 30 mL), dried over $MgSO_4$ and concentrated under reduced pressure. The crude residue was dissolved in Et_2O (20 mL). 1M Ethereal HCl (ca 2 mL) was added dropwise under stirring, while orange crystals precipitated. The solution was stirred for 20 minutes, then filtered and washed with additional 20 mL of Et_2O. The obtained solids were dissolved in DCM (100 mL). The organic solution was washed with aq. $NaHCO_3$ (3 × 100 mL), dried over $MgSO_4$ and concentrated under reduced pressure. 71 mg (96%) of deep purple crystals were obtained.

^{1}H NMR (400 MHz, $CDCl_3$, mixture of four stereoisomers) δ 8.20 (t, *J* = 3.0 Hz, 2H), 8.14 – 8.09 (m, 6H), 7.87 – 7.59 (m, 7H), 7.55 – 7.47 (m, 2H), 7.46 – 7.28 (m, 12H), 7.23 – 6.98 (m, 25H), 6.86 – 6.75 (m, 5H), 6.69 (d, *J* = 6.7 Hz, 1H), 6.65 – 6.54 (m, 7H), 6.51 – 6.47 (m, 1H), 6.44 – 6-29 (m, 3H), 6.13 – 6.04 (m, 3H), 5.99 (d, *J* = 10.5 Hz, 1H), 5.79 (d, *J* = 7.3 Hz, 1H), 5.70 (d, *J* = 7.3 Hz, 1H), 5.62 (d, *J* = 10.2 Hz, 1H), 5.59 (d, *J* = 10.3 Hz, 1H), 3.11 (d, *J* = 13.0 Hz, 1H), 3.11 (d, *J* = 13.0 Hz, 1H), 3.00 (d, *J* = 12.9 Hz, 1H), 2.98 (s, 3H), 2.75 – 2.57 (m, 13H), 2.55 (d, *J* = 13.4 Hz, 1H), 2.39 (d, *J* = 13.6 Hz, 1H), 1.47 (s, 3H), 1.26 (s, 3H), 1.16 (s, 3H), 1.13 (s, 3H).

^{13}C NMR (101 MHz, $CDCl_3$) δ 157.3, 156.9, 156.6, 155.5, 147.7, 147.3, 146.4, 146.0, 140.9, 140.7, 140.5, 137.7, 136.9, 135.1, 133.6, 133.3, 133.1, 132.5, 132.2, 132.0, 131.7, 131.5, 131.1, 131.0, 129.9, 129.3, 129.1, 128.7, 128.6, 128.5, 128.4, 128.3, 128.2, 127.9, 127.9, 127.7, 127.5, 127.4, 127.3, 127.0, 126.5, 126.4, 126.3, 126.2, 126.1, 125.8, 125.7, 125.6, 125.0, 124.9, 124.8, 124.6, 124.4, 124.1, 123.4, 122.1, 122.0, 121.9, 121.4, 121.1, 120.1, 119.6, 119.3, 118.9, 118.8, 118.6, 118.1, 108.3, 107.9, 107.2, 106.7, 106.6, 105.2, 104.4, 57.1, 55.8, 54.1, 53.3, 41.8, 41.0, 39.3, 38.8, 29.4, 29.3, 28.4, 28.2, 22.5, 22.4, 16.6, 16.5.

HRMS (APCI+): calculated for $[C_{35}H_{28}N_2O_3 + H]^+$: 525.2173; found: 525.2172.

*1',3'-Dimethyl-3'-benzyl-6-nitro-8-(4-tert-butylphenyl)spiro[chromene-2,2'-indoline] (**IIIf**)*

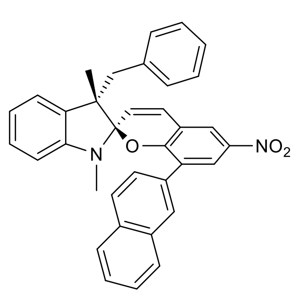

A solution of indolium salt **6a** (0.14 mmol, 53 mg), hydroxy aldehyde **14f** (0.14 mmol, 41 mg) and triethylamine (0.28 mmol, 39 μL) in dry ethanol (5 mL) was heated to reflux and stirred under argon atmosphere in darkness (covered with aluminium foil) for 2 hours. The solvent was evaporated, and the residue dissolved in Et_2O (40 mL). The solution was washed with water (3 × 30 mL), dried over $MgSO_4$ and concentrated under reduced pressure. The crude residue was dissolved in Et_2O (20 mL). 1M Ethereal HCl (ca 2 mL) was added dropwise under stirring, while orange crystals precipitated. The solution was stirred for 20 minutes, then filtered and washed with additional 20 mL of Et_2O. The obtained solids were dissolved in DCM (100 mL). The organic solution was washed with aq. $NaHCO_3$ (3 × 100 mL), dried over $MgSO_4$ and concentrated under reduced pressure. 50 mg (68%) of deep purple crystals were obtained.

^{1}H NMR (400 MHz, $CDCl_3$, mixture of four stereoisomers) *δ* 8.42 (d, *J* = 2.7 Hz, 1H), 8.37 (d, *J* = 2.7 Hz, 1H), 8.05 (d, *J* = 2.7 Hz, 1H), 7.99 (d, *J* = 2.7 Hz, 1H), 7.88 (d, *J* = 1.9 Hz, 1H), 7.80 (d, *J* = 1.9 Hz, 1H), 7.75 (d, *J* = 8.1 Hz, 1H), 7.69 (d, *J* = 7.7 Hz, 1H), 7.62 (d, *J* = 8.6 Hz, 1H), 7.57 – 7.47 (m, 3H), 7.46 – 7.30 (m, 6H), 7.27 – 7.22 (m, 2H) 7.21 – 6.99 (m, 8H), 6.93 (dt, *J* = 7.5, 1.0 Hz, 1H), 6.86 (d, *J* = 6.9 Hz, 2H), 6.72 – 6.62 (m, 6H), 6.15 (d, *J* = 10.3 Hz, 1H), 6.11 (dd, *J* = 7.2, 1.2 Hz, 1H), 5.66 (d, *J* = 10.3 Hz, 1H), 3.00 (d, *J* = 13.5 Hz, 1H), 2.93 (d, *J* = 13.6 Hz, 1H), 2.85 – 2.79 (m, 4H), 2.71 (s, 3H), 1.32 (s, 3H), 1.14 (s, 3H).

^{13}C NMR (101 MHz, $CDCl_3$) *δ* 156.8, 155.8, 148.2, 146.8, 141.4, 141.2, 137.5, 136.9, 136.4, 133.2, 133.0, 132.8, 132.6, 132.4, 132.0, 131.8, 131.7, 131.6, 130.9, 129.8, 129.4, 128.9, 128.8, 128.8, 128.7, 128.4, 128.4, 128.1, 128.1, 128.0, 127.7, 127.6, 127.5, 127.4, 127.4, 127.3, 126.6, 126.5, 126.5, 126.4, 126.4, 126.2, 126.1, 125.9, 125.0, 122.1, 121.7, 121.6, 120.8, 120.4, 120.3, 120.2, 119.8, 119.1, 108.4, 107.3, 104.6, 56.7, 54.0, 41.5, 39.3, 29.6, 28.2, 23.5, 16.6.

HRMS (APCI+): calculated for $[C_{35}H_{28}N_2O_3 + H]^+$: 525.2173; found: 525.2178.

2.4 Isolation of optically pure 3*H*-indole 5a

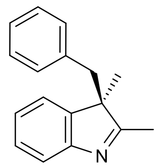

Racemic 3*H*-Indole **5a** (1.18 g; 5.0 mmol) and (–)-camphor-10-sulfonic acid (1.16 g; 5.0 mmol) were dissolved in EtOH (10 mL). The obtained red solution was stirred at room temperature for 10 min. The solvent was removed under reduced pressure. The obtained solid was suspended in boiling EtOAc. Acetonitrile was added dropwise until complete dissolution of the solids. The afforded hot solution was slowly let cool down to ambient temperature. Yielded crystals were filtered, washed with Et_2O and dried in vacuo. 702 mg of 74:26 diastereomeric mixture was isolated. The recrystallisation process was repeated and yielded 225 mg of optically pure diastereomeric salt, which was mixed with water (20 mL) and alkalised with saturated aq. solution of $NaHCO_3$ to pH = 7. The obtained mixture was extracted with DCM (3 × 10 mL). Pooled organic layers were washed with aq. $NaHCO_3$ and water (both 10 mL) and dried over $MgSO_4$. The solvent was removed under reduced pressure to give 101 mg (17%) of (*R*)-2,3-dimethyl-3-benzyl-3*H*-indole.

*(R)-2,3-dimethyl-3-benzyl-3H-indolium (–)-camphor-10-sulfonate (**5a_salt**)*

^{1}H NMR (400 MHz, CD_3CN) *δ* 7.70 (dd, *J* = 7.2, 1.3 Hz, 1H), 7.57 (dd, *J* = 7.7, 1.4 Hz, 1H), 7.52 (td, *J* = 7.3, 1.2 Hz, 1H), 7.47 (td, *J* = 7.5, 1.5 Hz, 1H), 7.18 – 7.07 (m, 3H), 6.81 (d, *J* = 6.8 Hz, 2H), 3.49 (d, *J* = 13.8 Hz, 1H), 3.33 (d, *J* = 13.8 Hz, 1H), 3.17 (d, *J* = 14.8 Hz, 1H), 2.85 (s, 3H), 2.71 (d, *J* = 14.7 Hz, 1H), 2.63 (ddd, *J* = 14.1, 11..6, 3.9 Hz, 1H), 2.30 (dt, *J* = 8.2, 3.9 Hz, 1H), 2.06 – 1.94 (m, 3H + solvent), 1.85

(d, J =18.2 Hz, 1H), 1.64 (s, 3H), 1.55 (ddd, J = 14.2, 9.4, 4.8 Hz, 1H), 1.36 (ddd, J = 12.1, 9.5, 4.0, 1H), 1.08 (s, 3H), 0.82 (s, 3H).

3. X-Ray crystallography of 5a_salt

*Crystallographic data for **5a_salt***

M = 467.63 g·mol^{-1}, orthorhombic system, space group $P2_12_12_1$, a = 8.0588 (2) Å, b = 12.1638 (3) Å, c = 25.1238 (6) Å, Z = 4, V = 2462.78 (10) Å^3, D_c = 1.261 g.cm^{-3}, μ(Cu-Kα) = 1.43 mm^{-1}, crystal dimensions of 0.47 × 0.41 × 0.37 mm. Data were collected at 180 (2) K on a Bruker D8 Venture Photon CMOS diffractometer with Incoatec microfocus sealed tube Cu-$K\alpha$ radiation. The structure was solved by charge flipping methods [S11] and anisotropically refined by full matrix least squares on F squared using the CRYSTALS [S12] to final value R = 0.031 and wR = 0.083 using 4803 independent reflections (ϑ_{max} = 68.4°), 396 parameters and 296 restrains. The hydrogen atoms bonded to carbon atoms were placed in calculated positions refined with a riding constrains, while hydrogen atoms bonded to nitrogen were refined using soft restraints. The disordered anion positions were found in difference electron density maps and refined with restrained geometry and ADPs. MCE [S13] was used for visualization of electron density maps. The occupancy of disordered functional group was constrained to full, resulting in final occupancy ratio of 729(3):271(3). The Flack parameter [S14] was refined to final value of 0.023(5). The structure was deposited into Cambridge Structural Database under number CCDC 2393565.

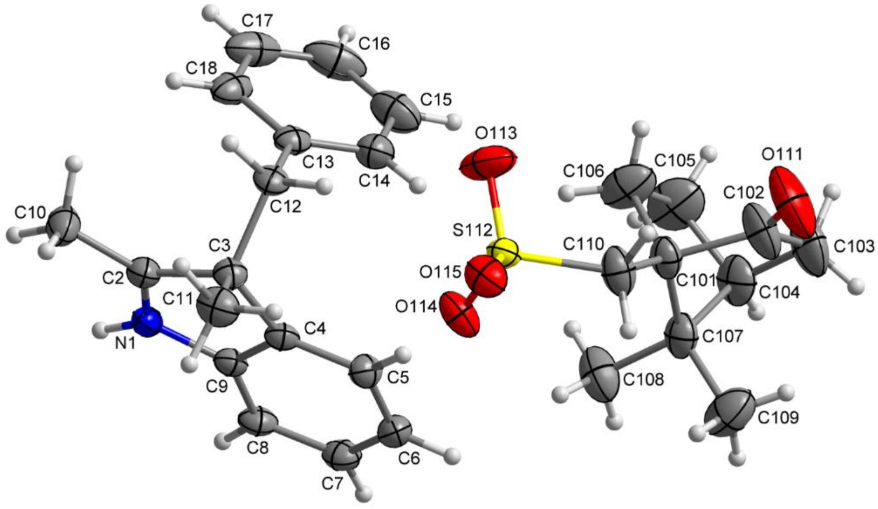

Figure 1: Numbering scheme of crystal structure ***5a_salt***, the ADPs drawn at 50% probability level. The weakly occupied atoms were omitted for clarity.

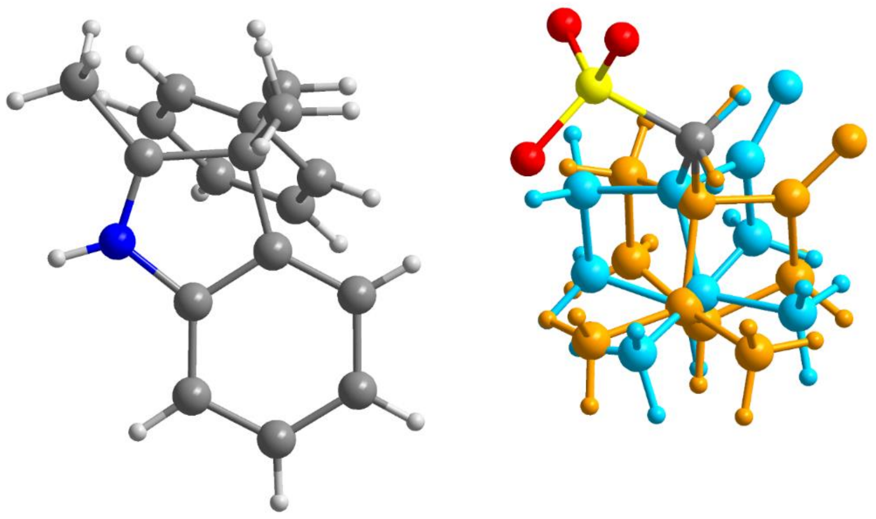

Figure 2: The disorder present in crystal structure **5a_salt**. The weakly occupied atoms depicted in blue, the strongly occupied in orange. The atoms depicted as spheres of arbitrary radii.

4. ^{1}H spectra of 2,3-dimethyl-3-benzyl-3*H*-indolium (–)-camphor-10-sulfonate

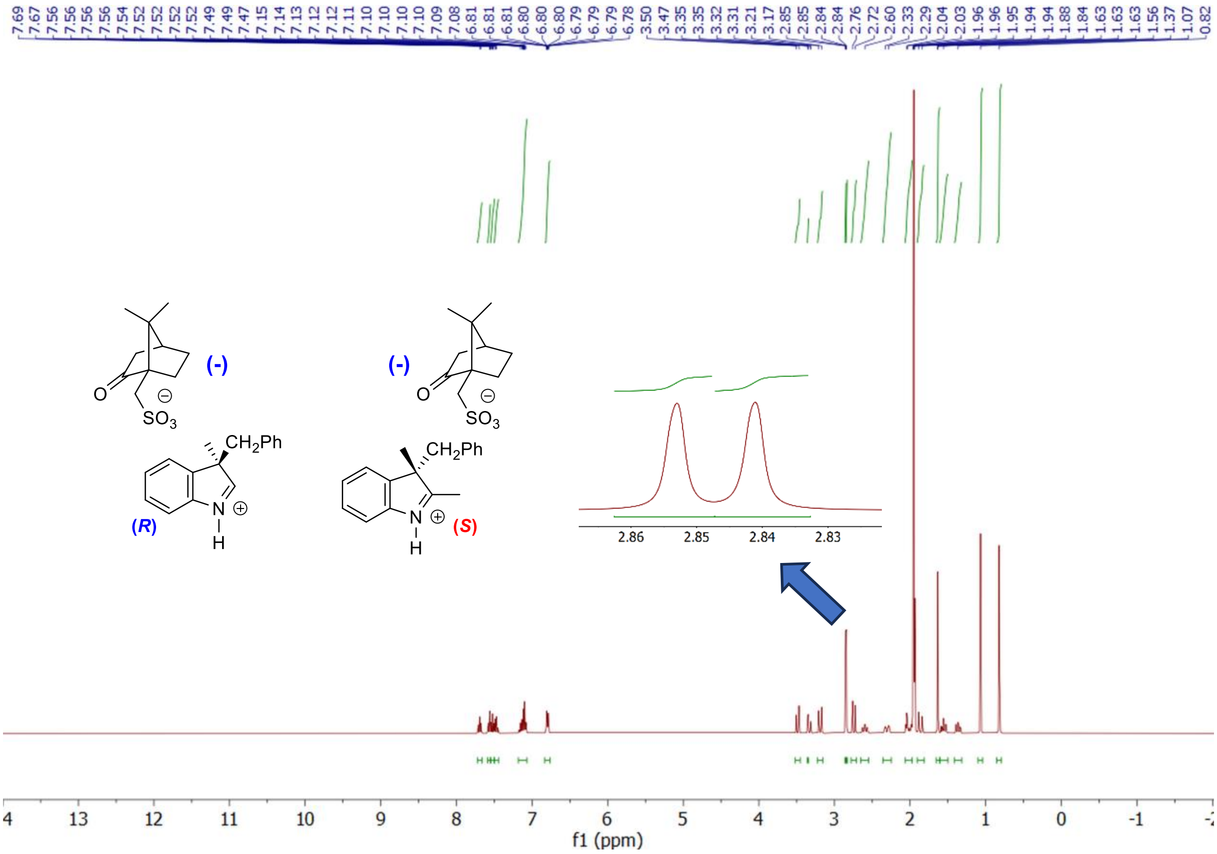

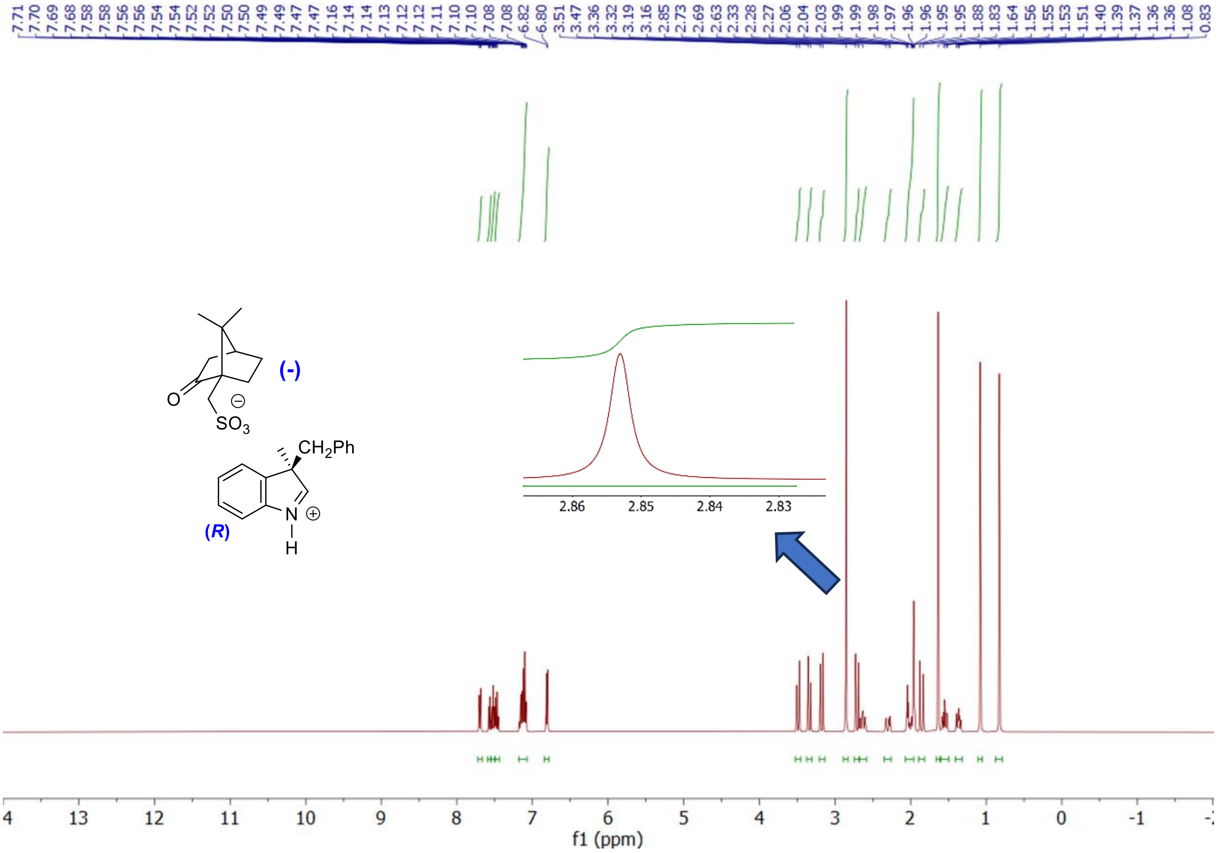

5. ^{1}H and ^{13}C NMR spectra of the target spiropyrans

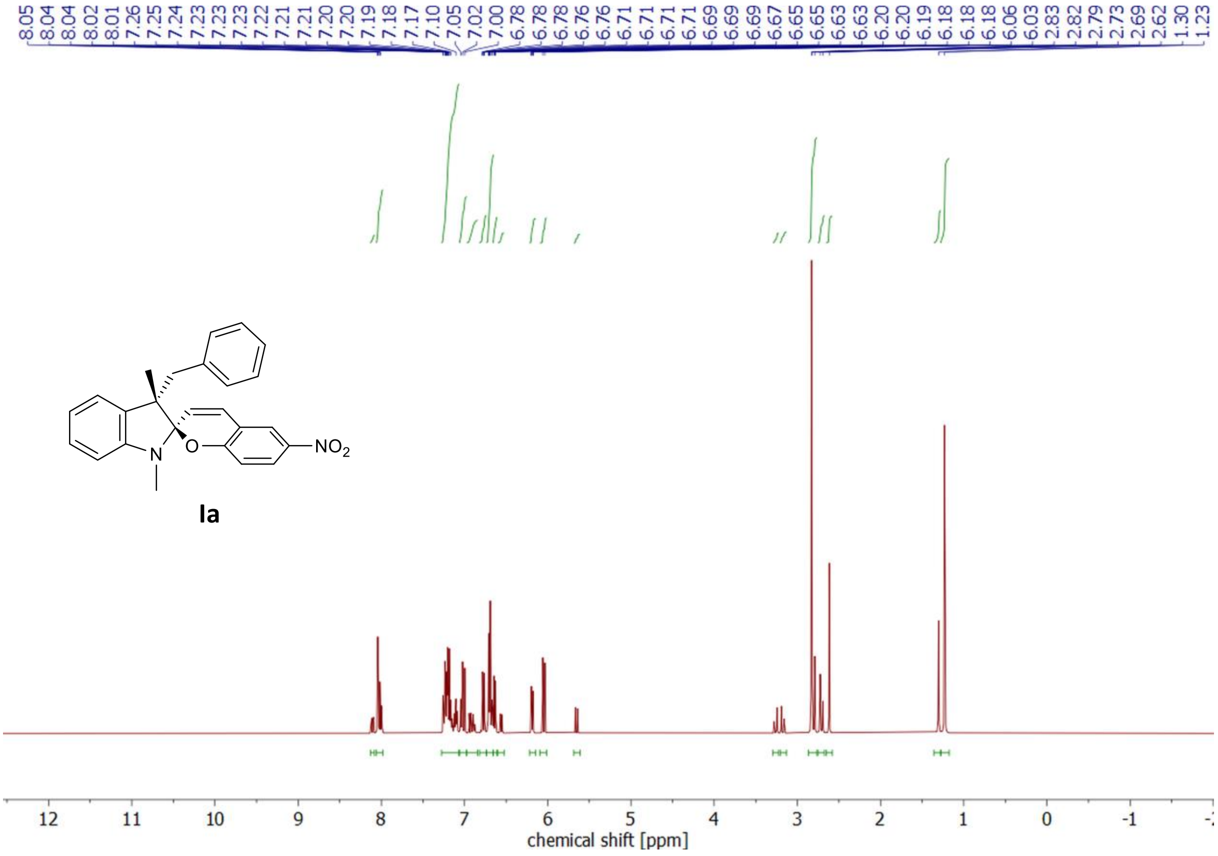

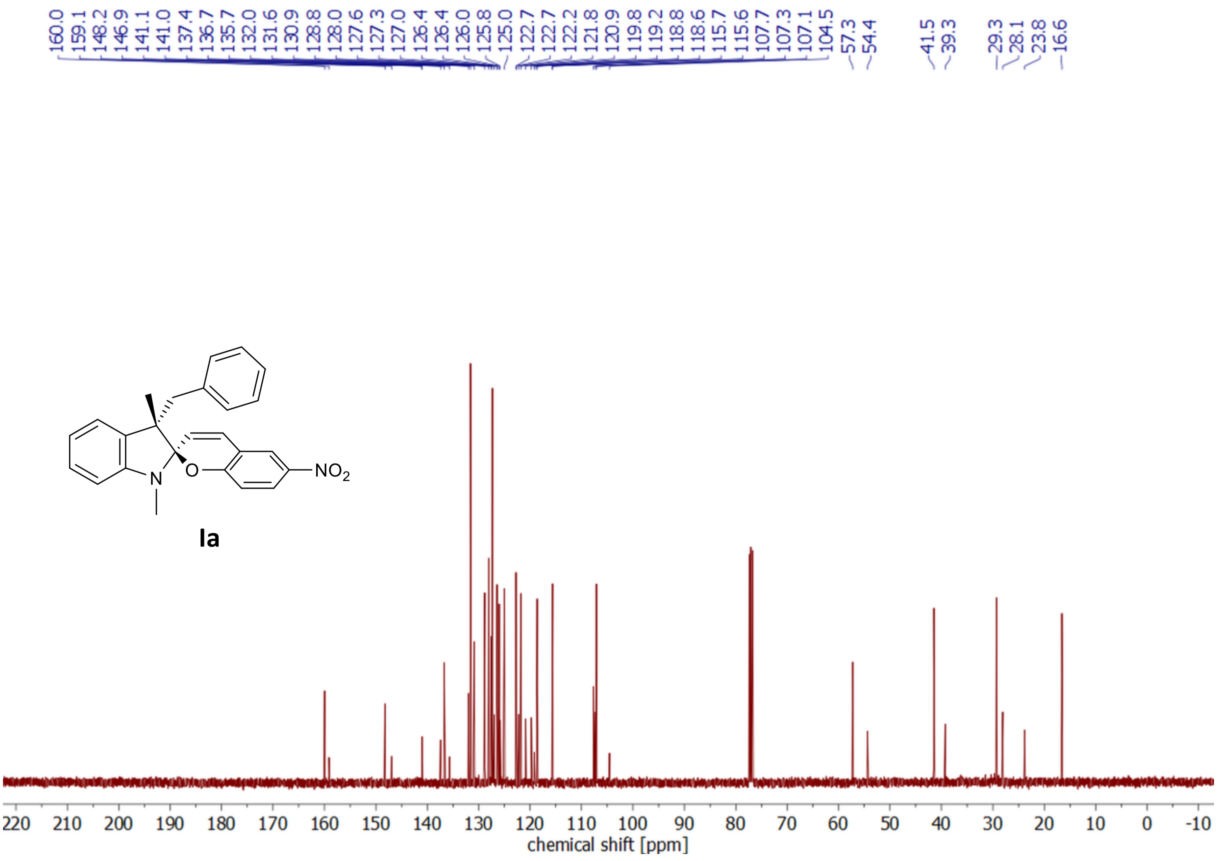

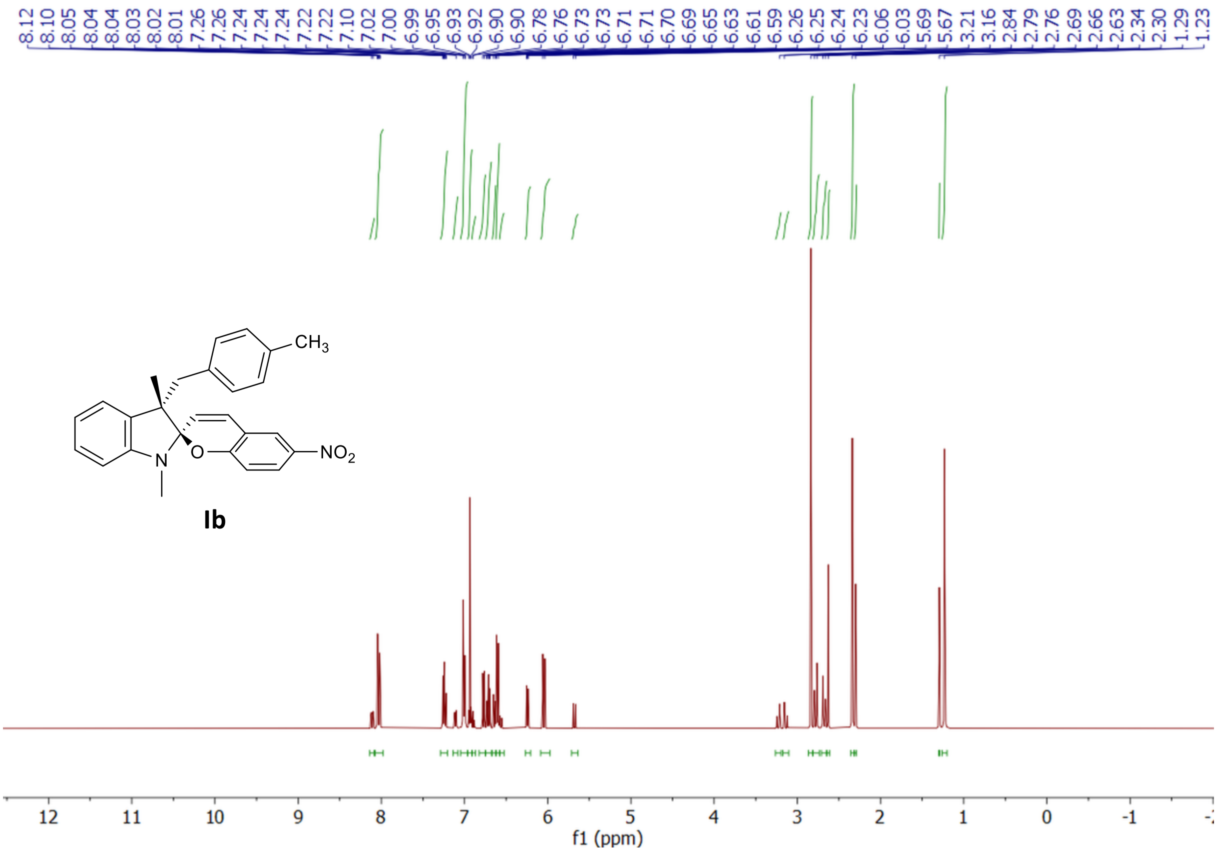
8.12 8.10 8.05 8.04 8.03 8.02 8.01 7.26 7.26 7.24 7.24 7.24 7.22 7.22 7.10 7.02 7.00 6.99 6.95 6.93 6.92 6.92 6.90 6.90 6.78 6.76 6.73 6.73 6.71 6.71 6.70 6.69 6.65 6.63 6.61 6.59 6.26 6.25 6.24 6.23 6.06 6.03 5.69 5.67 3.21 3.16 2.84 2.79 2.76 2.69 2.66 2.63 2.34 2.30 1.29 1.23
CH3
N
O
NO2
Ib
f1 (ppm)

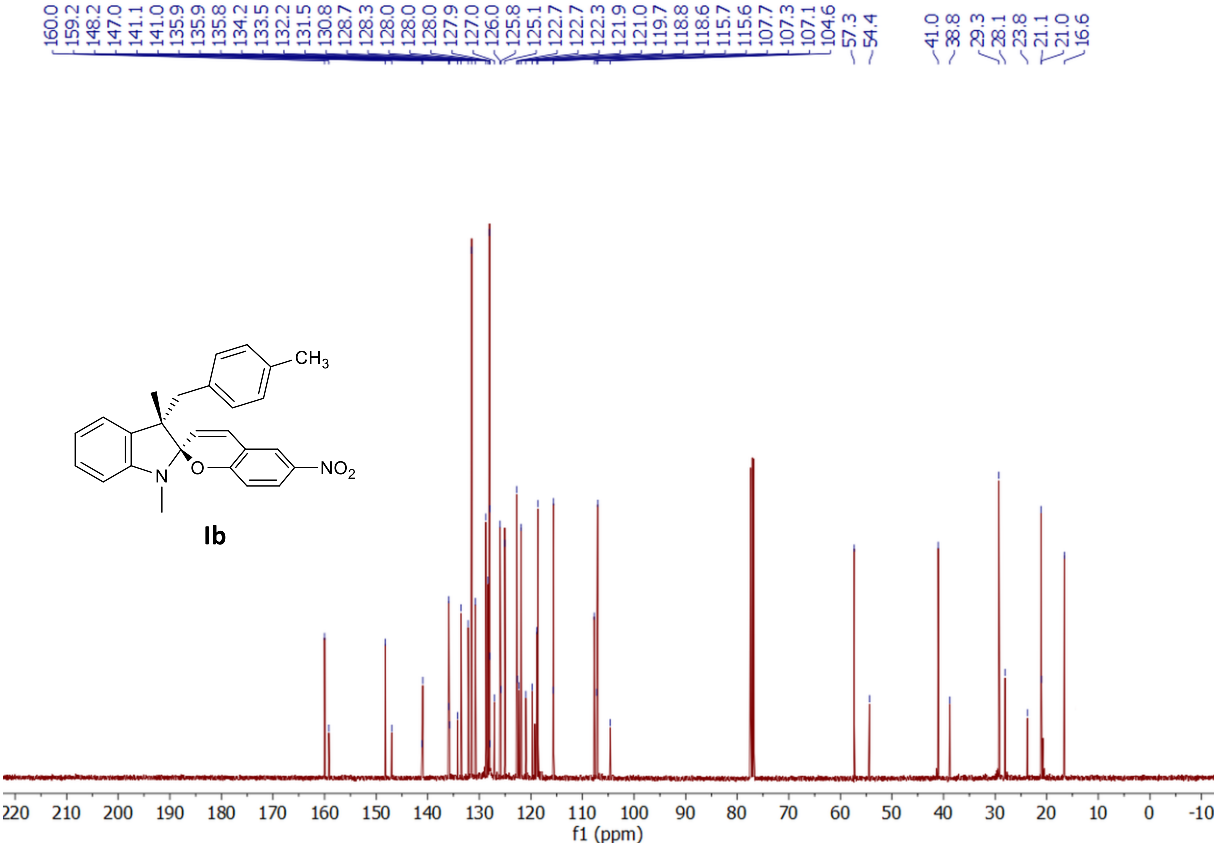
160.0 159.2 148.2 147.0 141.1 141.0 135.9 135.9 135.8 134.2 133.5 132.2 131.5 130.8 128.7 128.3 128.0 128.0 128.0 127.9 127.0 126.0 125.8 125.1 122.7 122.7 122.3 121.9 121.0 119.7 118.8 118.6 115.7 115.6 107.7 107.3 107.1 104.6 57.3 54.4 41.0 38.8 29.3 28.1 23.8 21.1 21.0 16.6
CH3
N
O
NO2
Ib
f1 (ppm)

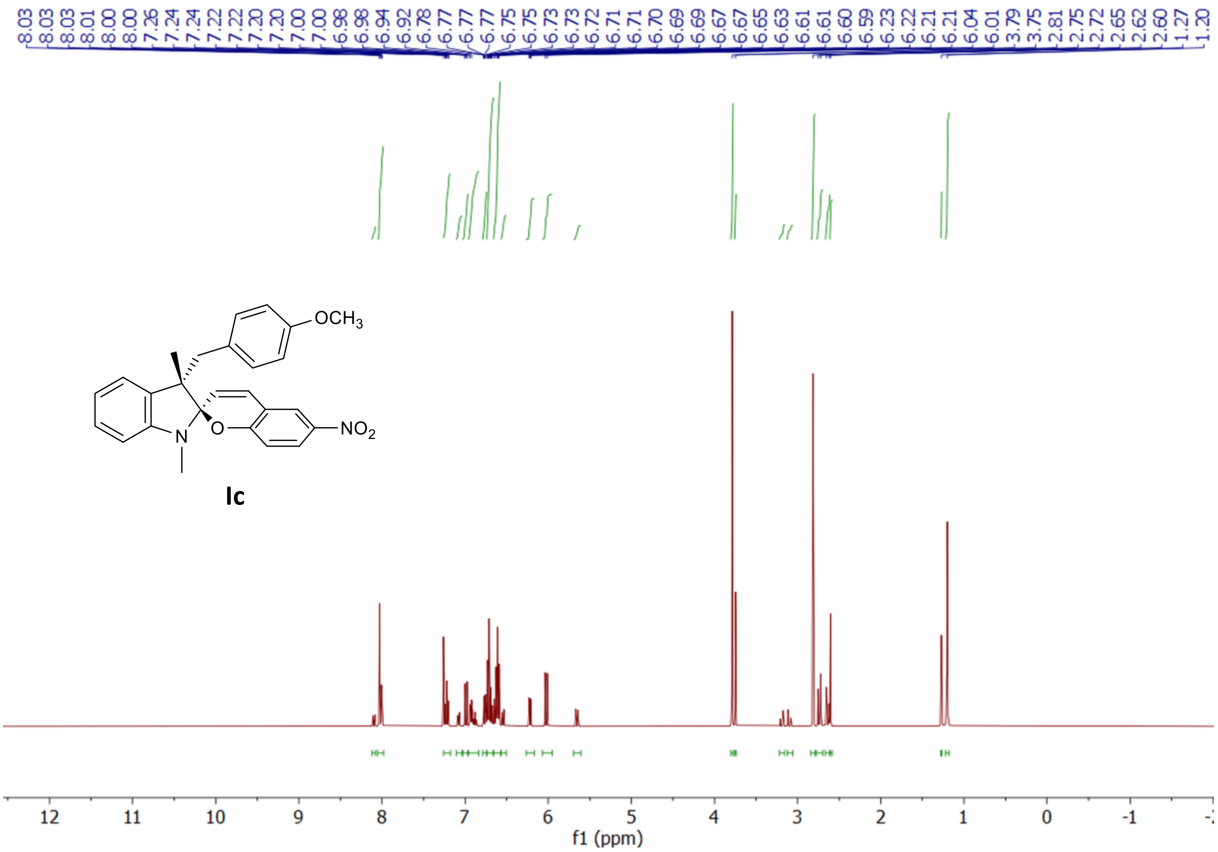

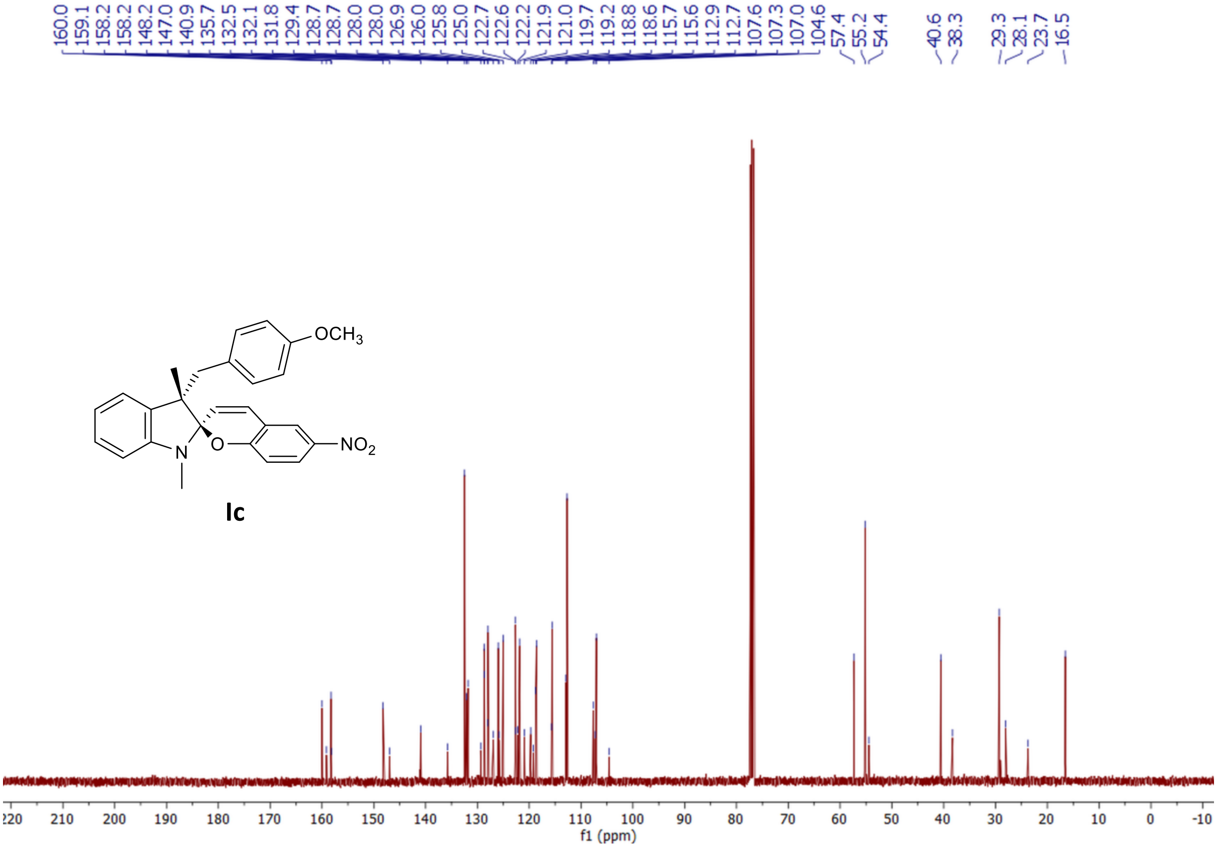

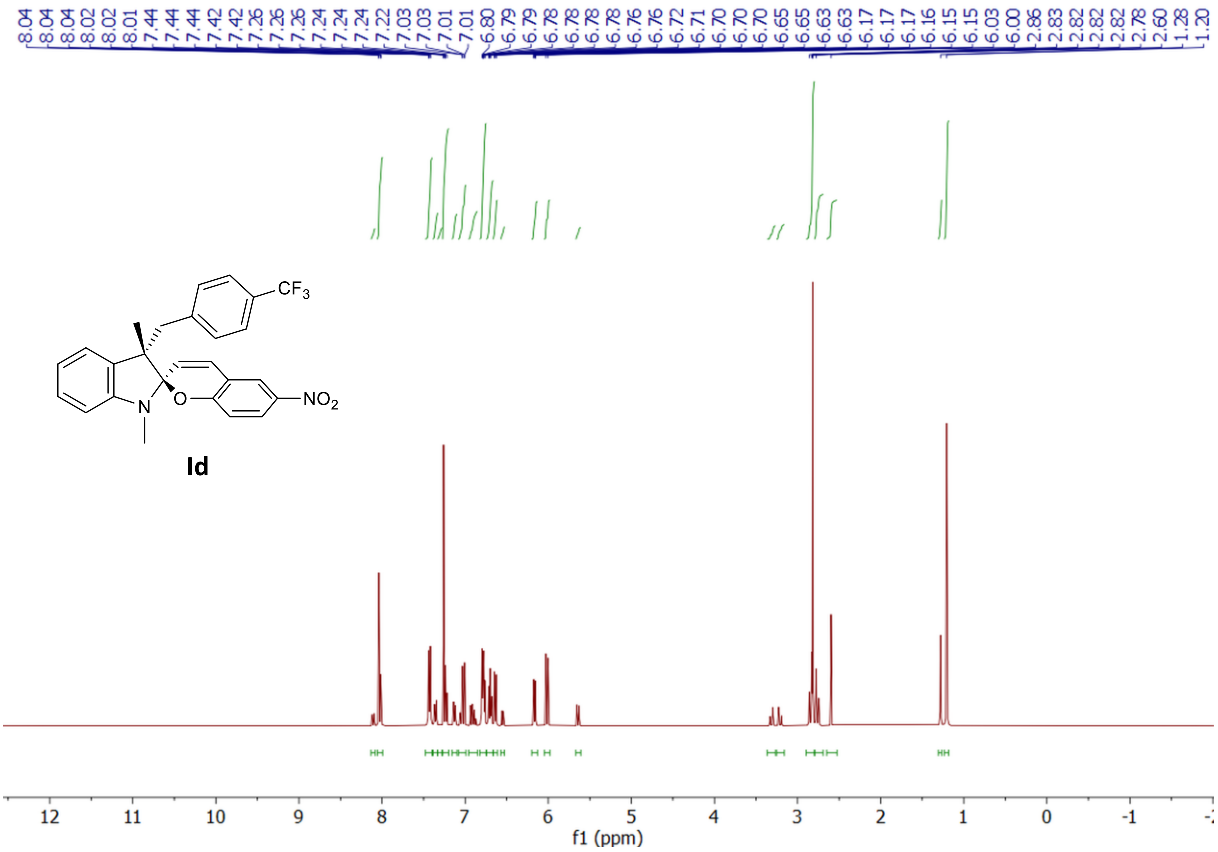
8.04 8.04 8.04 8.02 8.01 7.44 7.44 7.44 7.42 7.42 7.26 7.26 7.26 7.24 7.24 7.24 7.22 7.03 7.03 7.01 7.01 6.80 6.79 6.79 6.78 6.78 6.78 6.78 6.76 6.76 6.72 6.71 6.70 6.70 6.70 6.65 6.65 6.63 6.63 6.17 6.17 6.17 6.16 6.15 6.15 6.03 6.00 2.86 2.83 2.82 2.82 2.82 2.78 2.60 1.28 1.20
CF3
N
O
NO2
Id
f1 (ppm)

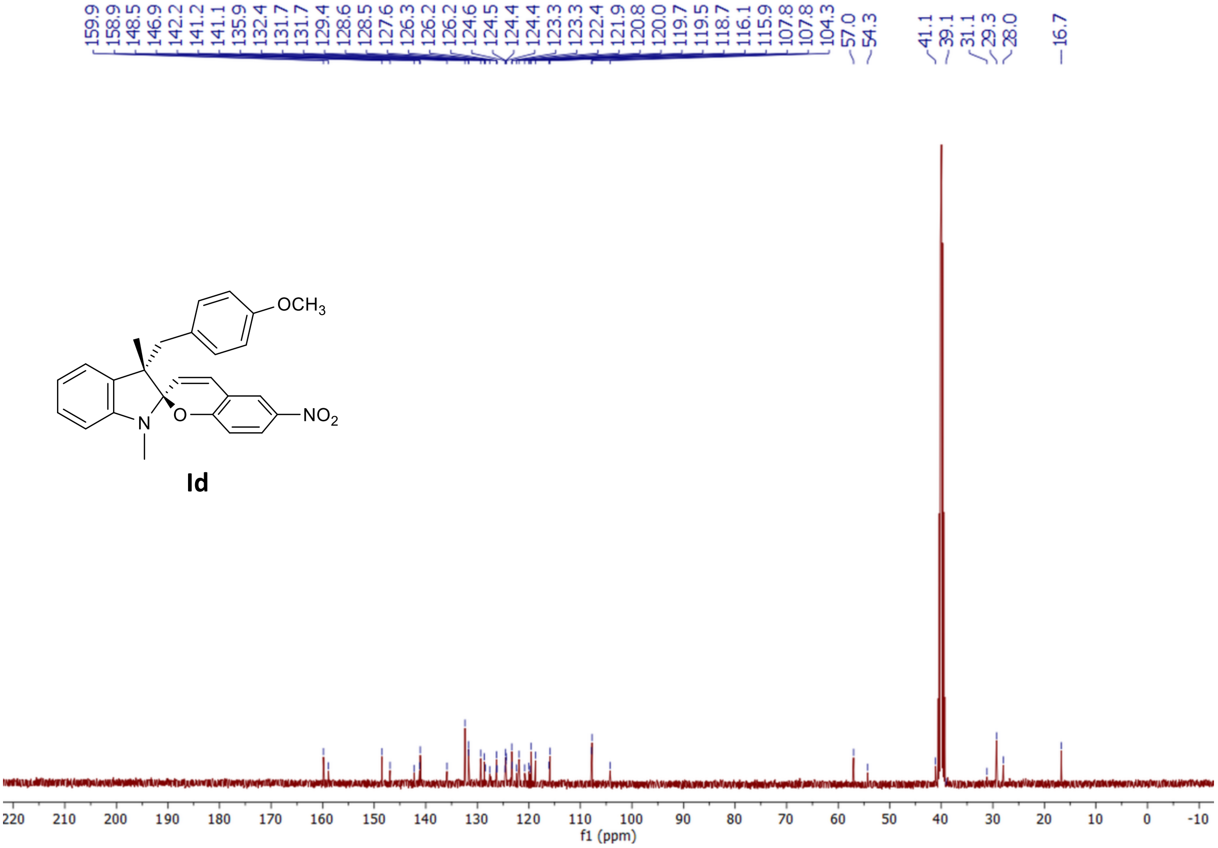
159.9 158.9 148.5 146.9 142.2 141.2 141.1 135.9 132.4 131.7 131.7 129.4 128.6 128.5 127.6 126.3 126.2 126.2 124.6 124.5 124.4 124.4 124.4 123.3 123.3 122.4 121.9 120.8 120.0 119.7 119.5 118.7 116.1 115.9 107.8 107.8 104.3 57.0 54.3 41.1 39.1 31.1 29.3 28.0 16.7
OCH3
N
O
NO2
Id
f1 (ppm)

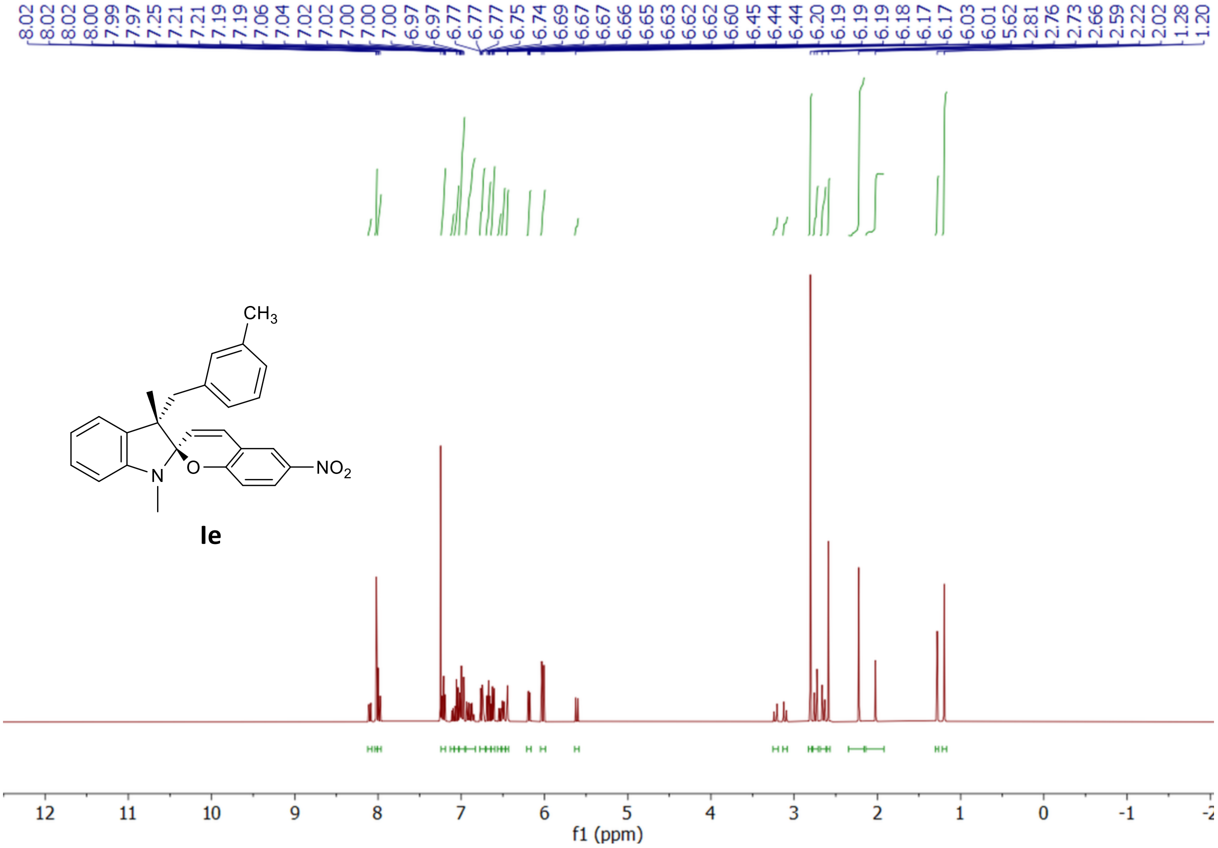
8.02 8.02 8.02 8.00 7.99 7.97 7.25 7.21 7.21 7.19 7.19 7.06 7.04 7.02 7.02 7.00 7.00 7.00 6.97 6.97 6.77 6.77 6.77 6.75 6.74 6.69 6.67 6.66 6.65 6.63 6.62 6.62 6.60 6.45 6.44 6.44 6.20 6.19 6.19 6.19 6.18 6.17 6.17 6.03 6.01 5.62 2.81 2.76 2.73 2.66 2.59 2.22 2.02 1.28 1.20
12 11 10 9 8 7 6 5 4 3 2 1 0 -1
CH3
N
O
NO2
f1 (ppm)

Ie

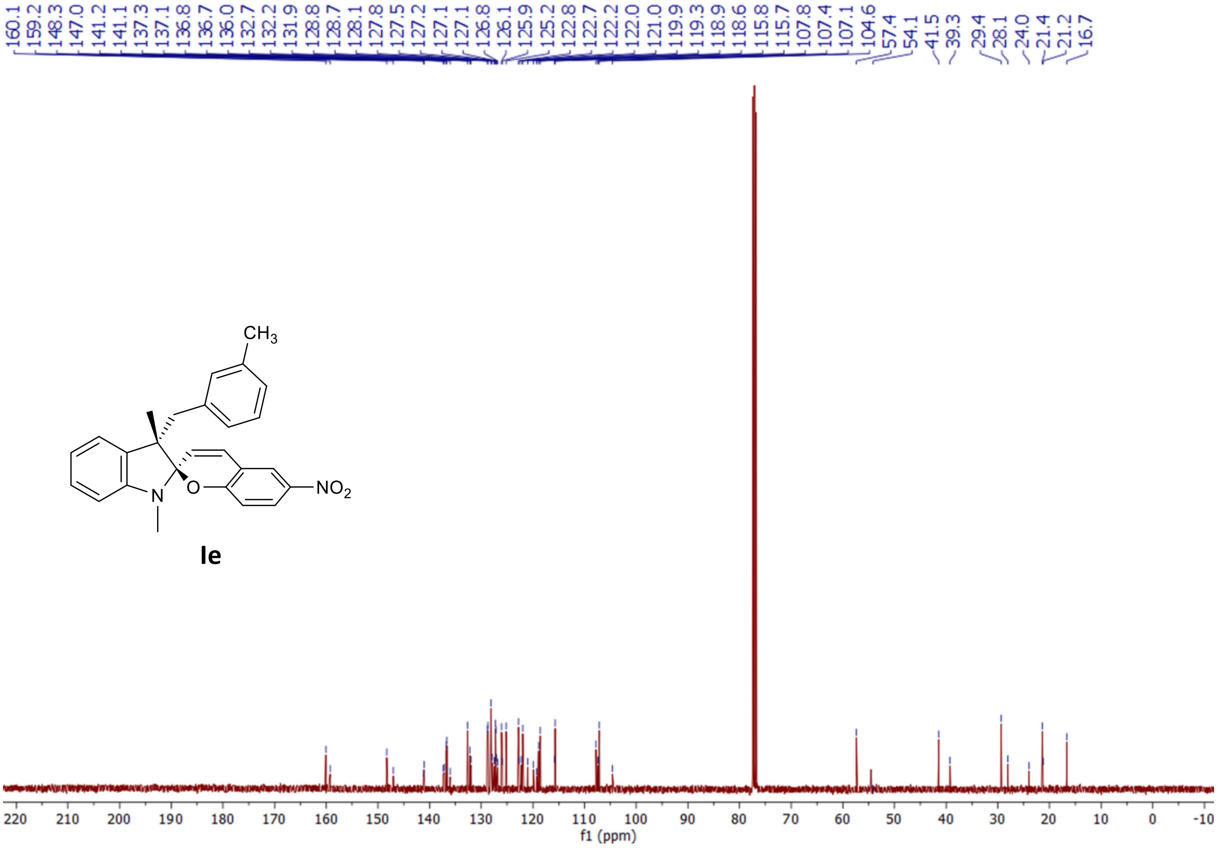
160.1 159.2 148.3 147.0 141.2 141.1 137.3 137.1 136.8 136.7 136.0 132.7 132.2 131.9 128.8 128.7 128.1 127.8 127.5 127.2 127.1 126.8 126.1 125.9 125.2 122.8 122.7 122.2 122.0 121.0 119.9 119.3 118.9 118.6 115.8 115.7 107.8 107.4 107.1 104.6 57.4 54.1 41.5 39.3 29.4 28.1 24.0 21.4 21.2 16.7
220 210 200 190 180 170 160 150 140 130 120 110 100 90 80 70 60 50 40 30 20 10 0 -10
CH3
N
O
NO2
f1 (ppm)

Ie

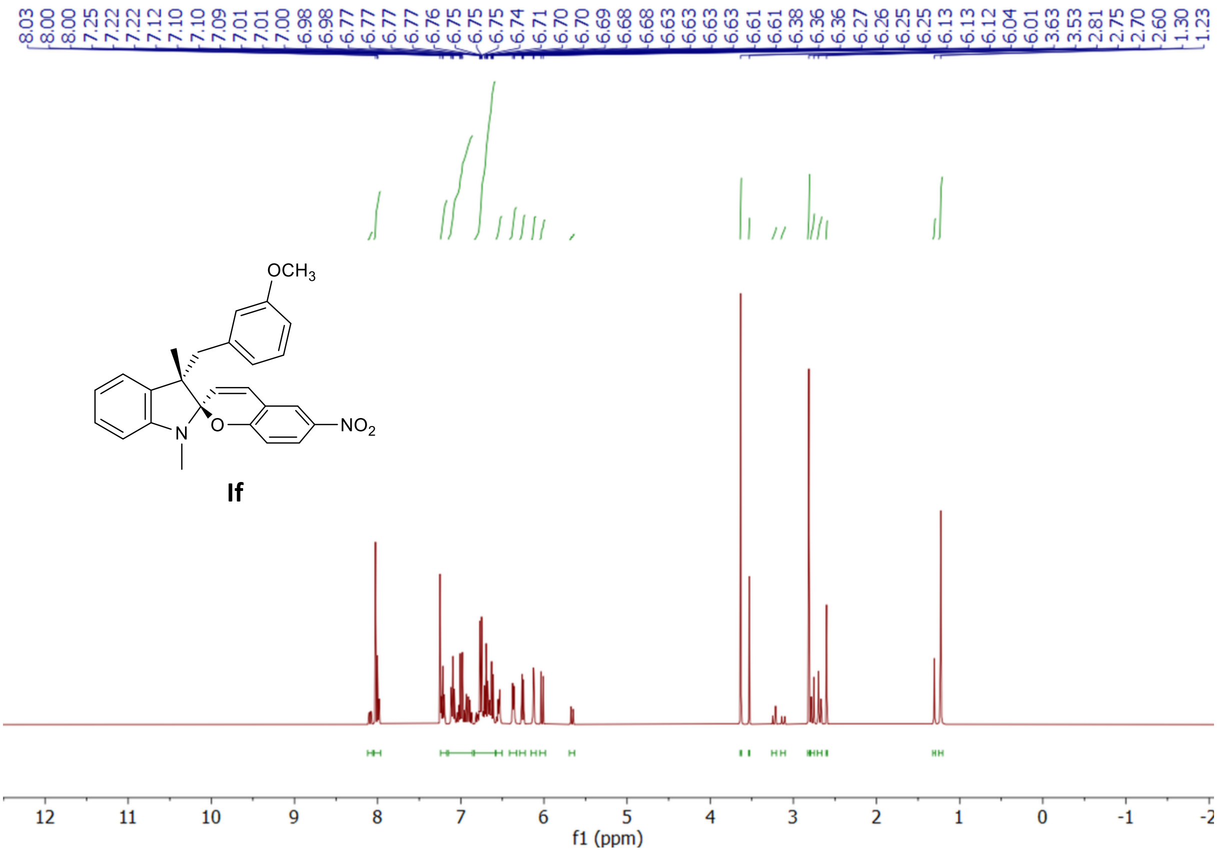
8.03 8.00 8.00 7.25 7.22 7.12 7.10 7.10 7.09 7.01 7.01 7.00 6.98 6.98 6.77 6.77 6.77 6.76 6.75 6.75 6.74 6.71 6.70 6.70 6.69 6.68 6.68 6.63 6.63 6.63 6.63 6.61 6.61 6.38 6.36 6.36 6.27 6.26 6.25 6.25 6.13 6.13 6.12 6.04 6.01 3.63 3.53 2.81 2.75 2.70 2.60 1.30 1.23
OCH3
N
O
NO2
If
f1 (ppm)

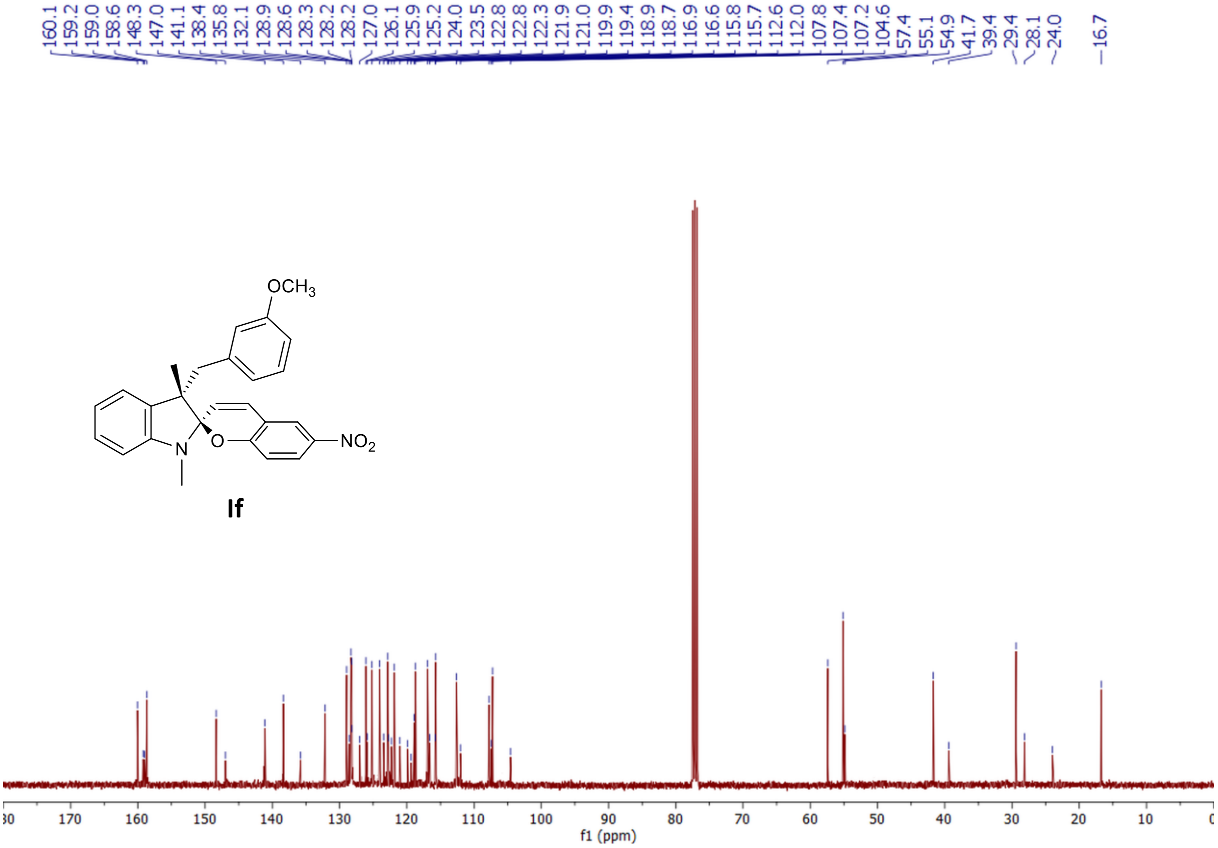
160.1 159.2 159.0 158.6 148.3 147.0 141.1 138.4 135.8 132.1 128.9 128.6 128.3 128.2 128.2 127.0 126.1 125.9 125.2 124.0 123.5 122.8 122.3 121.9 121.0 119.9 119.4 118.9 118.7 116.9 116.6 115.8 115.7 112.6 112.0 107.8 107.4 107.2 104.6 57.4 55.1 54.9 41.7 39.4 29.4 28.1 24.0 16.7
OCH3
N
O
NO2
If
f1 (ppm)

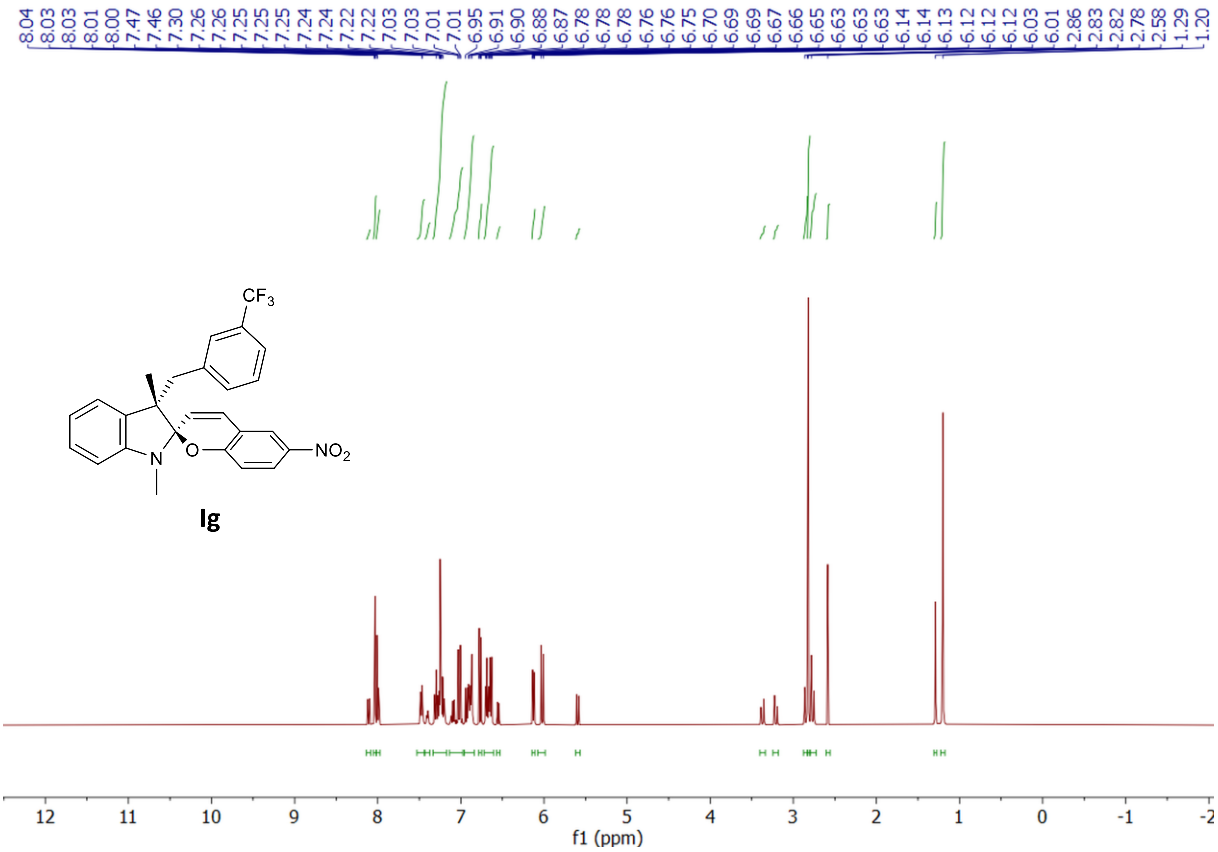
8.04 8.03 8.03 8.01 8.00 7.47 7.46 7.30 7.26 7.25 7.25 7.25 7.24 7.24 7.22 7.22 7.03 7.03 7.01 7.01 6.95 6.91 6.90 6.88 6.87 6.78 6.78 6.78 6.76 6.76 6.75 6.70 6.69 6.69 6.67 6.66 6.65 6.63 6.63 6.63 6.14 6.14 6.13 6.12 6.12 6.12 6.03 6.01 2.86 2.83 2.82 2.78 2.58 1.29 1.20
CF3
NO2
N
O
Ig
f1 (ppm)

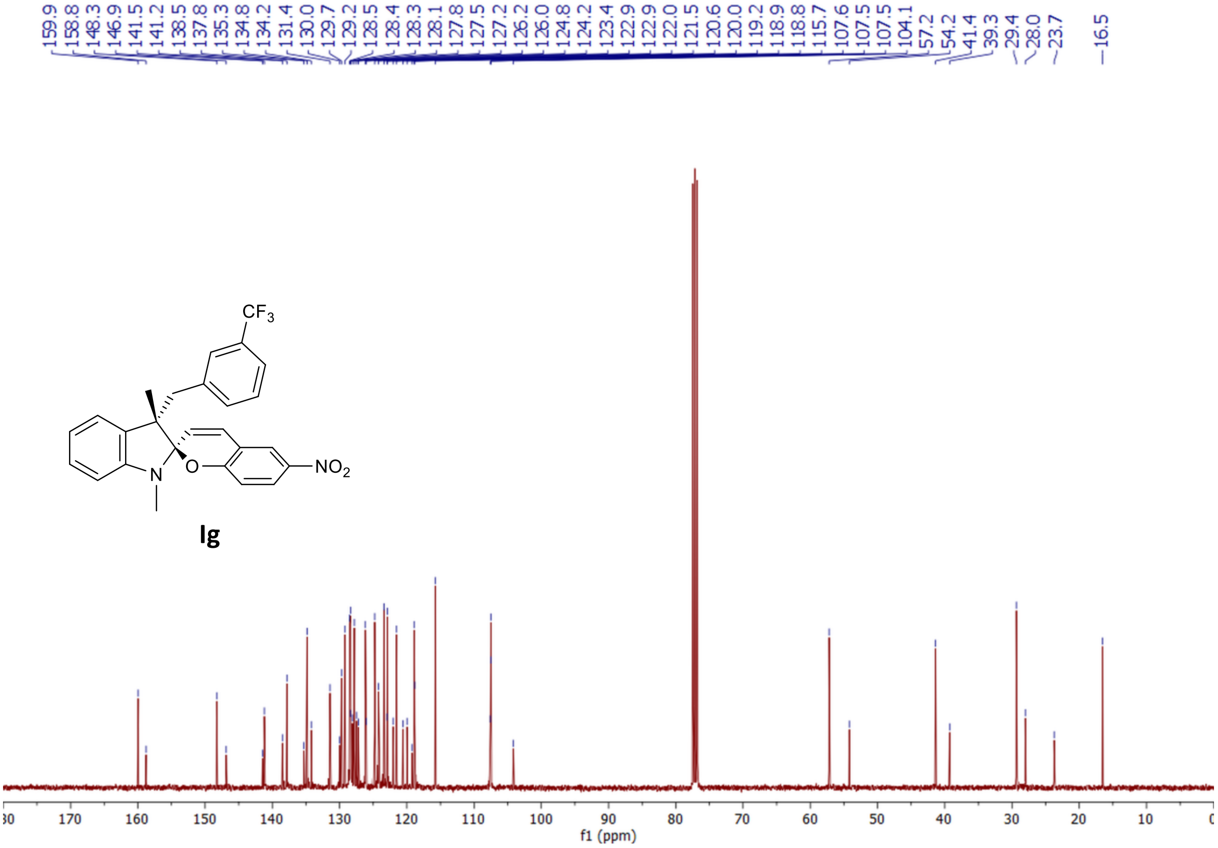
159.9 158.8 148.3 146.9 141.5 141.2 138.5 137.8 135.3 134.8 134.2 131.4 130.0 129.7 129.2 128.5 128.4 128.3 128.1 127.8 127.5 127.2 126.2 126.0 124.8 124.2 123.4 122.9 122.9 122.0 121.5 120.6 120.0 119.2 118.9 118.8 115.7 107.6 107.5 104.1 57.2 54.2 41.4 39.3 29.4 28.0 23.7 16.5
CF3
NO2
N
O
Ig
f1 (ppm)

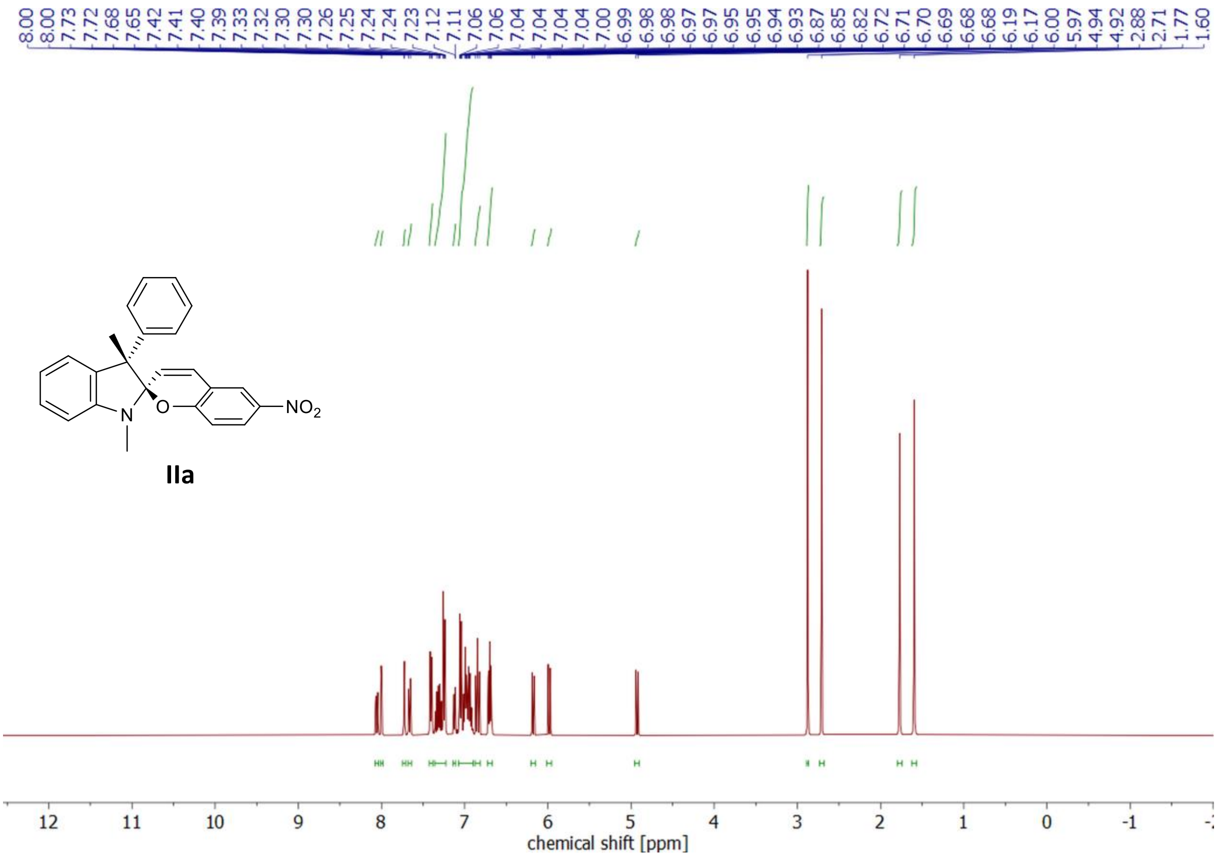
8.00 8.00 7.73 7.72 7.68 7.65 7.42 7.41 7.40 7.39 7.33 7.32 7.30 7.30 7.26 7.25 7.24 7.24 7.23 7.12 7.11 7.06 7.06 7.04 7.04 7.04 7.04 7.00 6.99 6.98 6.98 6.97 6.97 6.95 6.95 6.94 6.93 6.87 6.85 6.82 6.72 6.71 6.70 6.69 6.68 6.68 6.19 6.17 6.00 5.97 4.94 4.92 2.88 2.71 1.77 1.60
N
O
NO2
IIa
chemical shift [ppm]

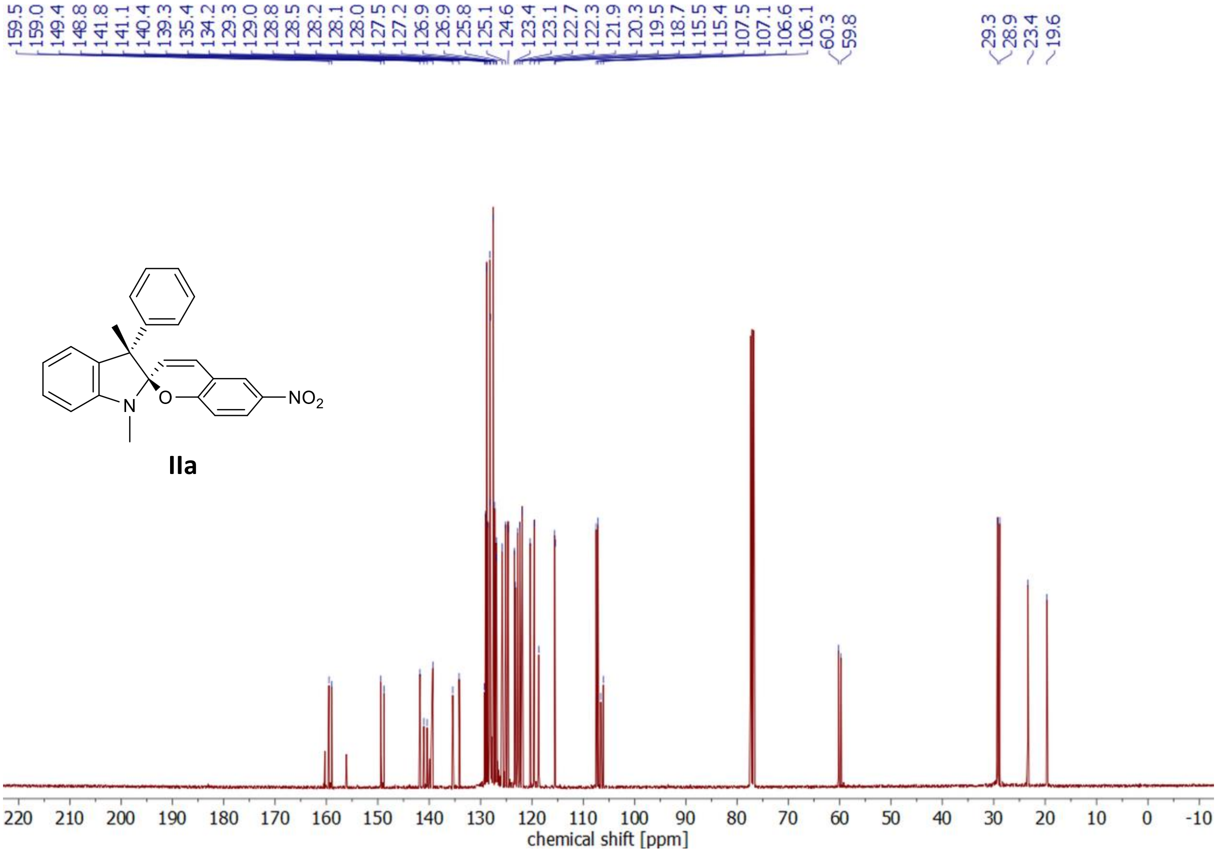
159.5 159.0 149.4 148.8 141.8 141.1 140.4 139.3 135.4 134.2 129.3 129.0 128.8 128.5 128.2 128.1 128.0 127.5 127.2 126.9 126.9 125.8 125.1 124.6 123.4 123.1 122.7 122.3 121.9 120.3 119.5 118.7 115.5 115.4 107.5 107.1 106.6 106.1 60.3 59.8 29.3 28.9 23.4 19.6
N
O
NO2
IIa
chemical shift [ppm]

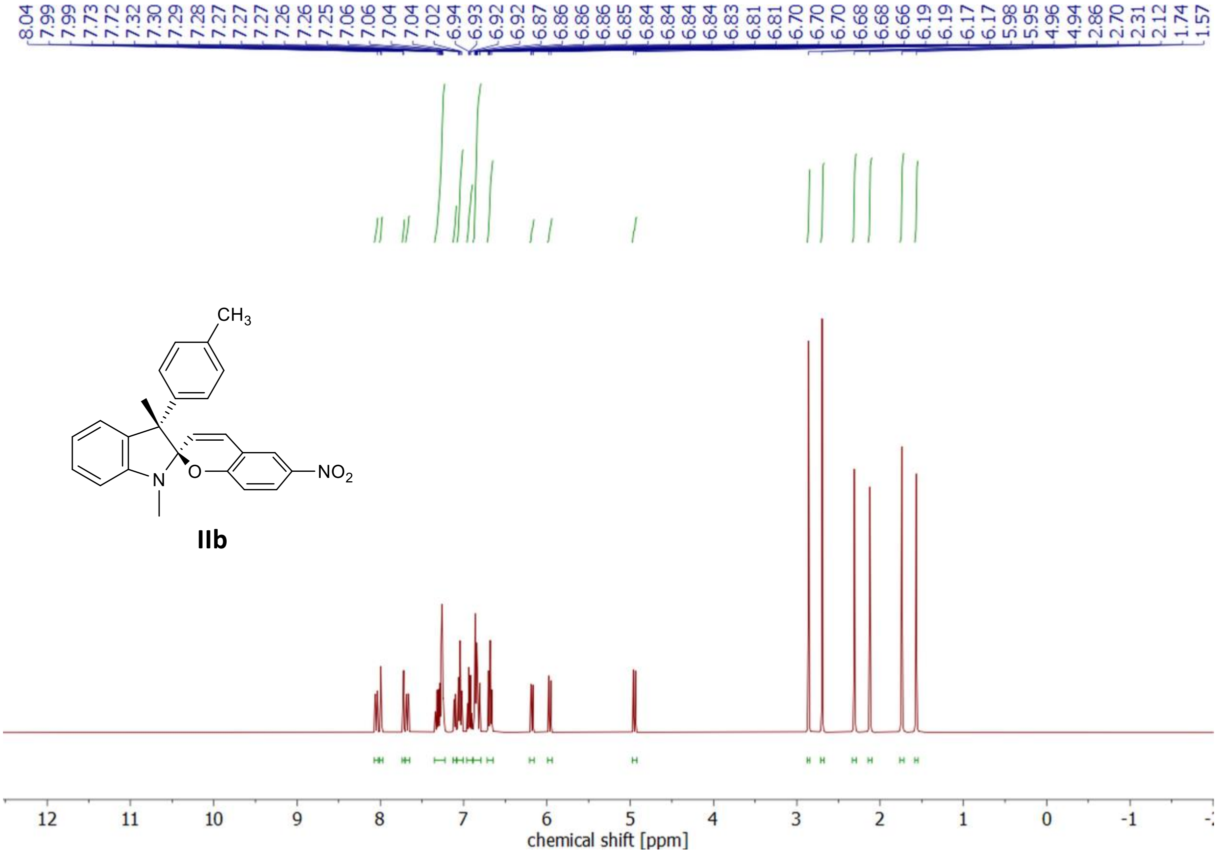
8.04 7.99 7.99 7.73 7.72 7.32 7.30 7.29 7.28 7.27 7.27 7.27 7.26 7.25 7.06 7.06 7.04 7.04 7.02 6.94 6.93 6.92 6.92 6.87 6.86 6.86 6.86 6.85 6.84 6.84 6.84 6.84 6.83 6.81 6.81 6.70 6.70 6.68 6.68 6.66 6.19 6.19 6.17 6.17 5.98 5.95 4.96 4.94 2.86 2.70 2.31 2.12 1.74 1.57
CH3
N
O
NO2
IIb
chemical shift [ppm]

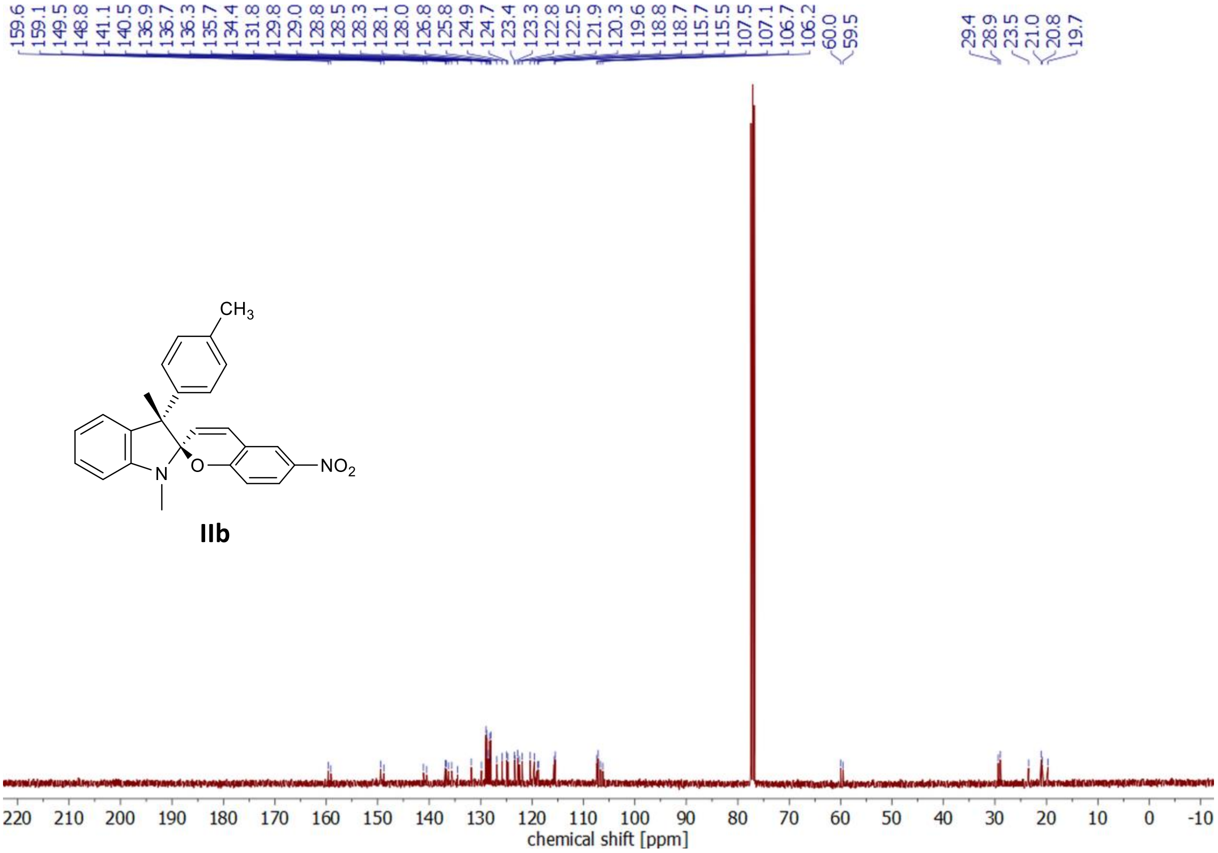
159.6 159.1 149.5 148.8 141.1 140.5 136.9 136.7 136.3 135.7 134.4 131.8 129.8 129.0 128.8 128.5 128.3 128.1 128.0 126.8 125.8 124.9 124.7 123.4 123.3 122.8 122.5 121.9 120.3 119.6 118.8 118.7 115.7 115.5 107.5 107.1 106.7 106.2 60.0 59.5 29.4 28.9 23.5 21.0 20.8 19.7
CH3
N
O
NO2
IIb
chemical shift [ppm]

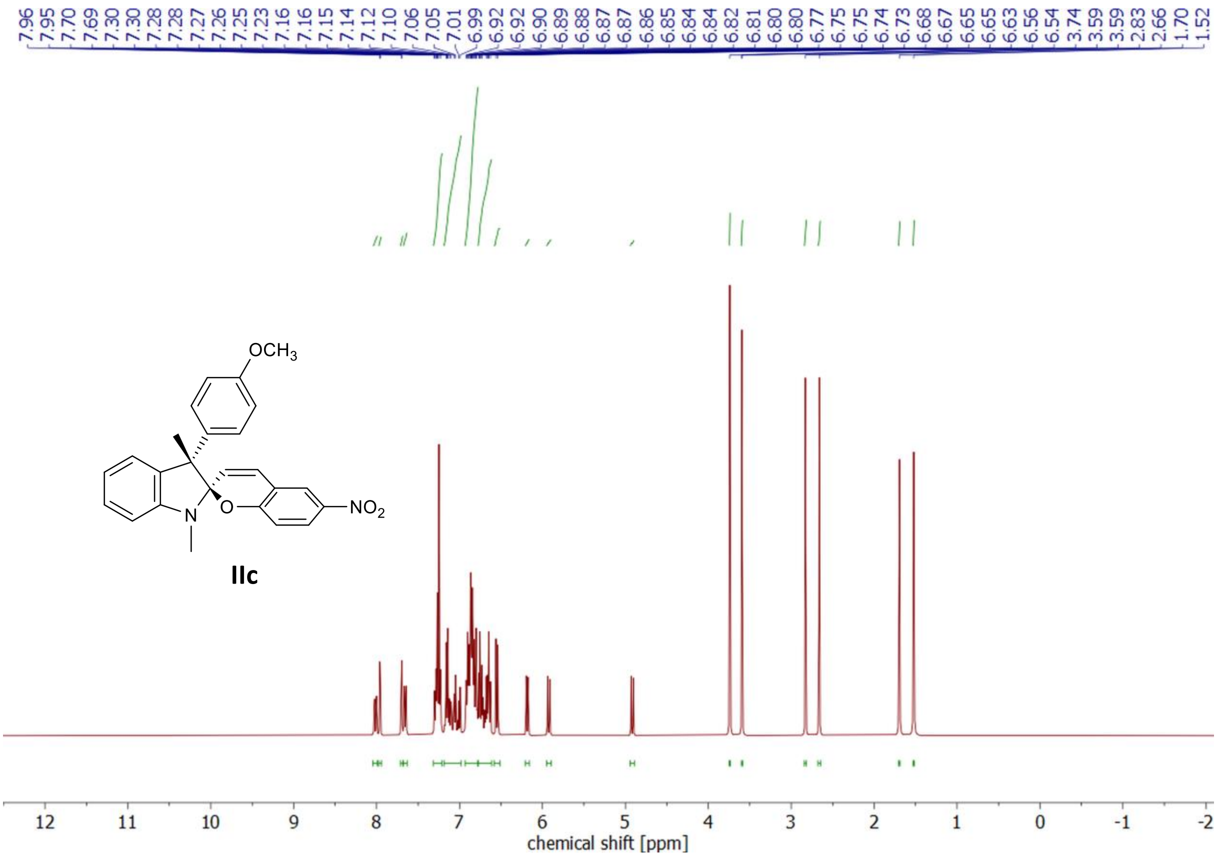
7.96 7.95 7.70 7.69 7.30 7.30 7.28 7.28 7.27 7.26 7.25 7.23 7.16 7.15 7.14 7.12 7.10 7.06 7.05 7.01 6.99 6.92 6.92 6.90 6.89 6.88 6.87 6.87 6.86 6.85 6.84 6.84 6.82 6.81 6.80 6.80 6.77 6.75 6.75 6.74 6.73 6.68 6.67 6.65 6.65 6.63 6.56 6.54 3.74 3.59 3.59 2.83 2.66 1.70 1.52
OCH3
N
O
NO2
IIc
12 11 10 9 8 7 6 5 4 3 2 1 0 -1 -2
chemical shift [ppm]

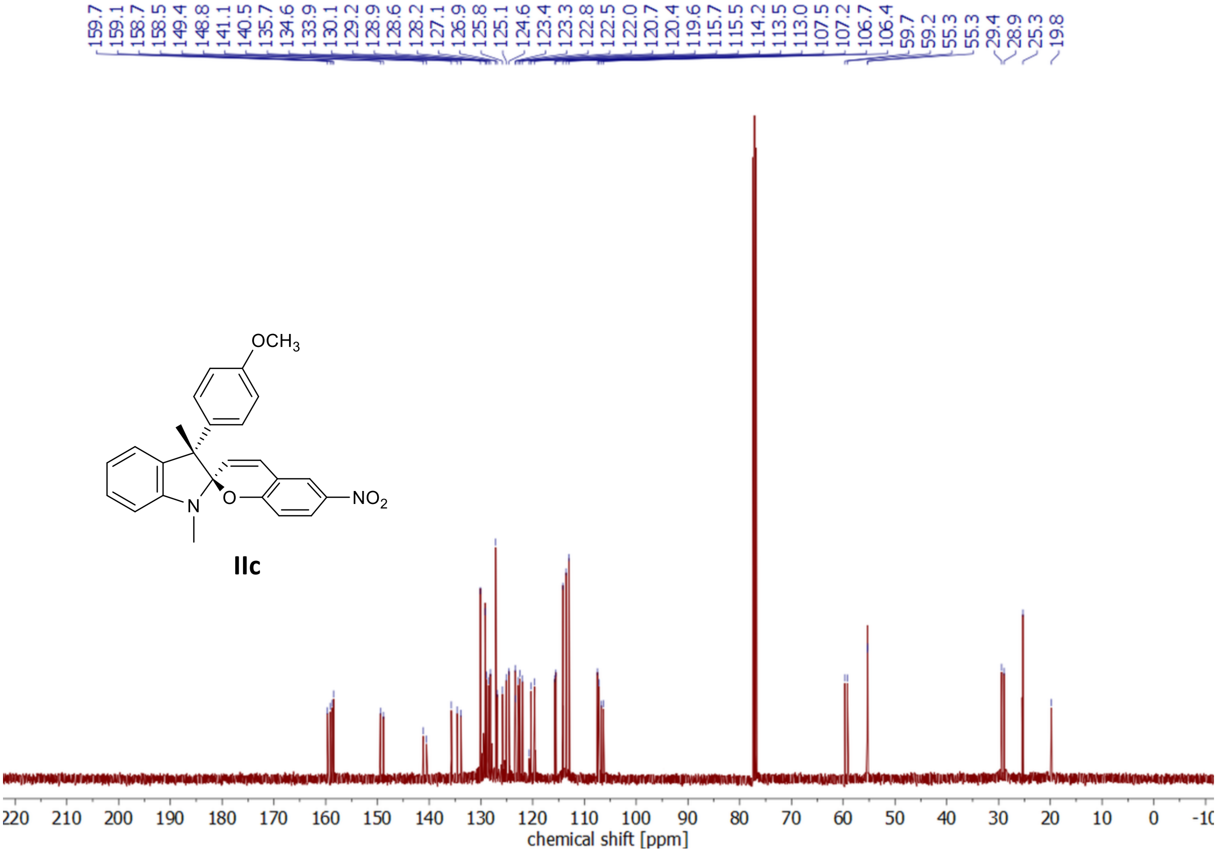
159.7 159.1 158.7 158.5 149.4 148.8 141.1 140.5 135.7 134.6 133.9 130.1 129.2 128.9 128.6 128.2 127.1 126.9 125.8 125.1 124.6 123.4 123.3 122.8 122.5 122.0 120.7 120.4 119.6 115.7 115.5 114.2 113.5 113.0 107.5 107.2 106.7 106.4 59.7 59.2 55.3 55.3 29.4 28.9 25.3 19.8
OCH3
N
O
NO2
IIc
220 210 200 190 180 170 160 150 140 130 120 110 100 90 80 70 60 50 40 30 20 10 0 -10
chemical shift [ppm]

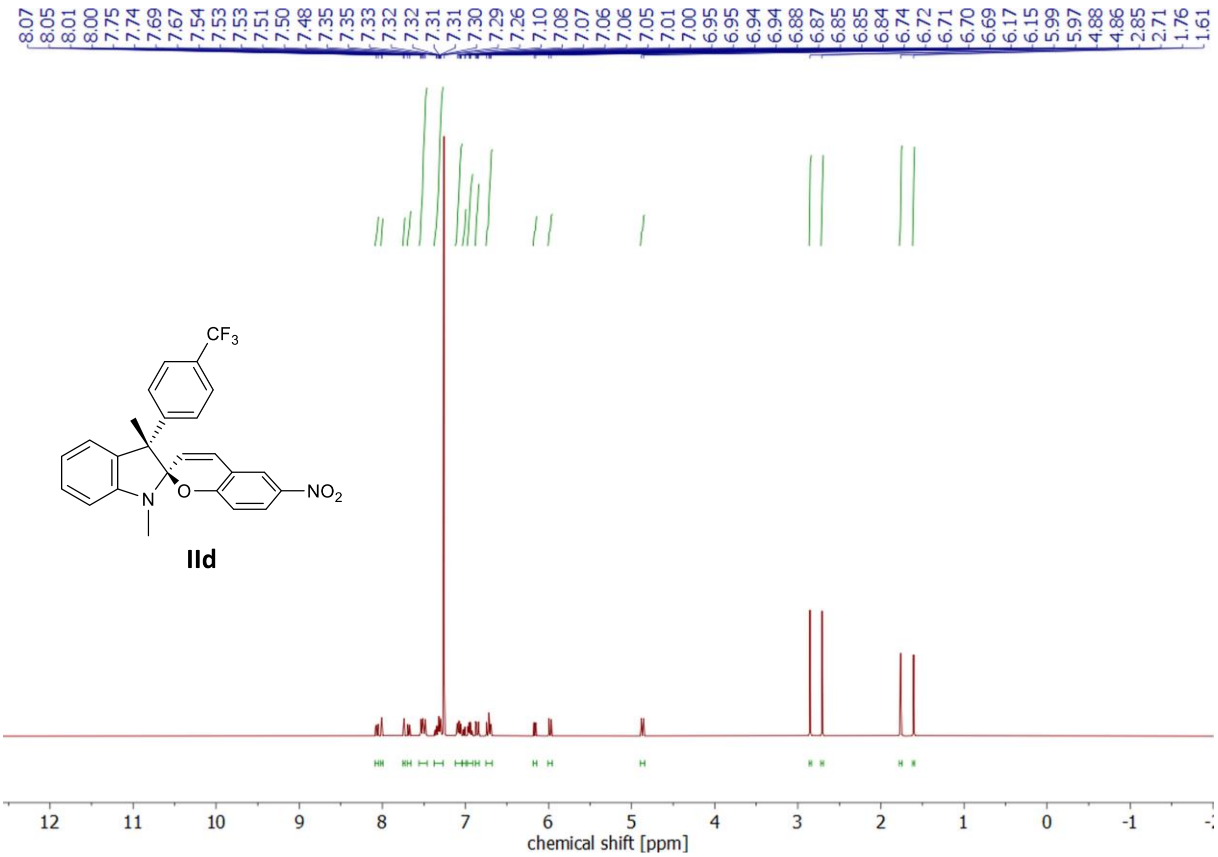

8.07 8.05 8.01 8.00 7.75 7.74 7.69 7.67 7.54 7.53 7.53 7.51 7.50 7.48 7.35 7.35 7.33 7.32 7.31 7.31 7.30 7.29 7.26 7.10 7.08 7.07 7.06 7.06 7.05 7.01 7.00 6.95 6.95 6.94 6.94 6.88 6.87 6.85 6.85 6.84 6.74 6.72 6.71 6.70 6.69 6.17 6.15 5.99 5.97 4.88 4.86 2.85 2.71 1.76 1.61
CF3
N
O
NO2
IId
12 11 10 9 8 7 6 5 4 3 2 1 0 -1
chemical shift [ppm]

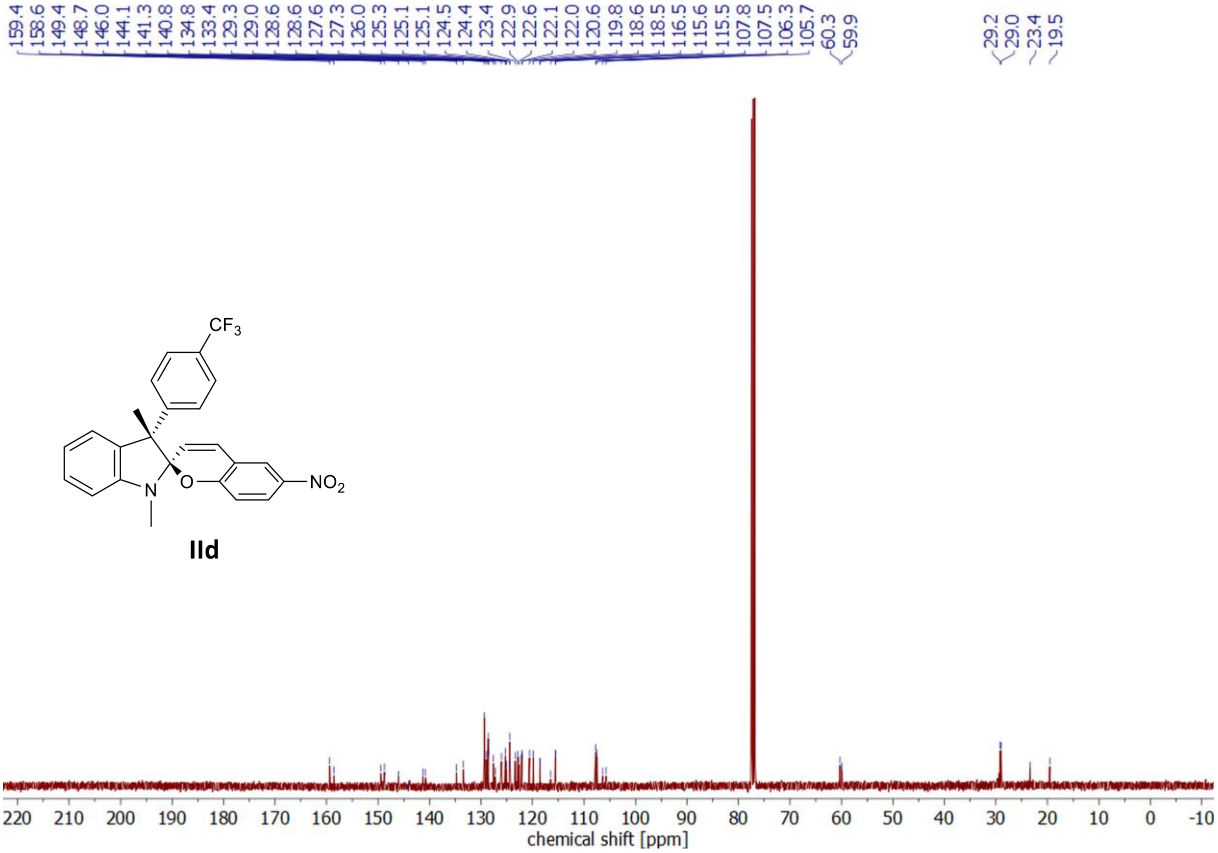

159.4 158.6 149.4 148.7 146.0 144.1 141.3 140.8 134.8 133.4 129.3 129.0 128.6 128.6 127.6 127.3 126.0 125.3 125.1 125.1 124.5 124.4 123.4 122.9 122.6 122.1 122.0 120.6 119.8 118.6 118.5 116.5 115.6 115.5 107.8 107.5 106.3 105.7 60.3 59.9 29.2 29.0 23.4 19.5
CF3
N
O
NO2
IId
220 210 200 190 180 170 160 150 140 130 120 110 100 90 80 70 60 50 40 30 20 10 0 -10
chemical shift [ppm]

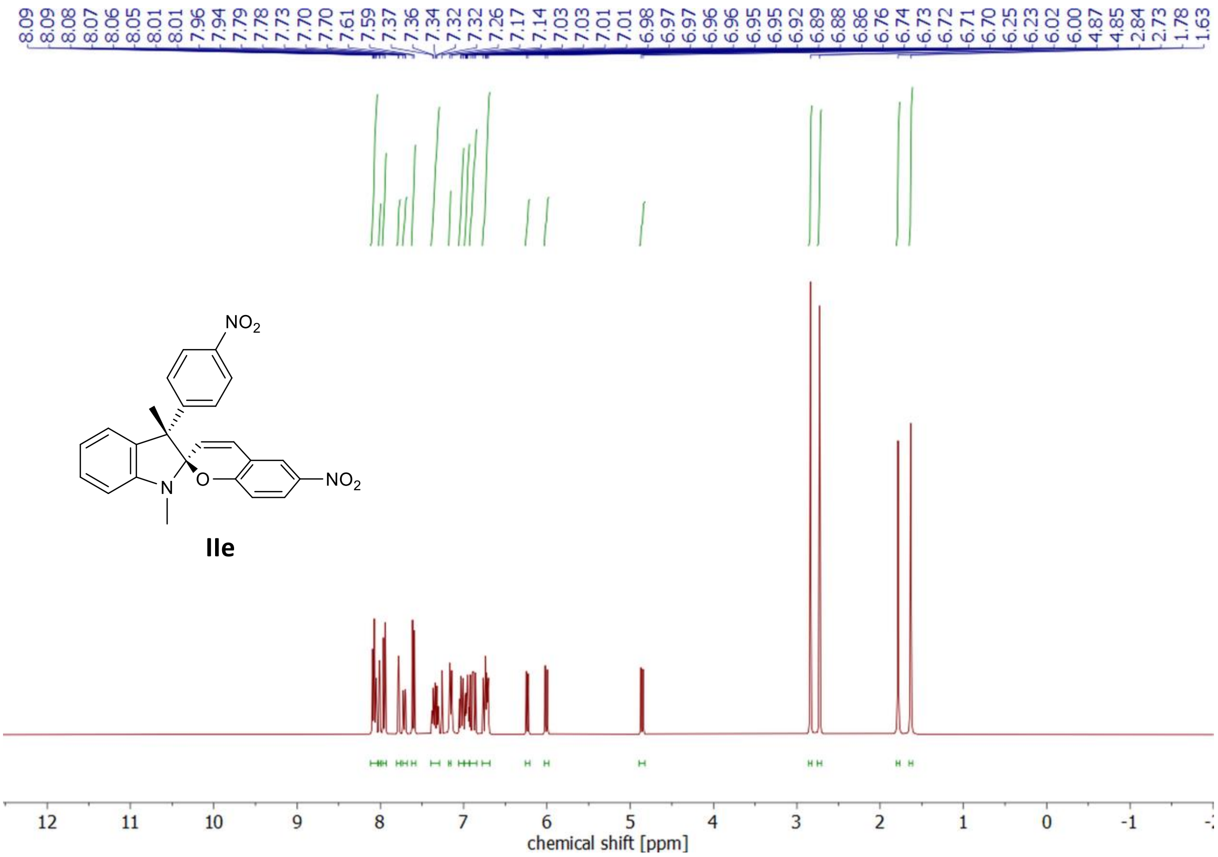
8.09 8.09 8.08 8.07 8.06 8.05 8.01 8.01 7.96 7.94 7.79 7.78 7.73 7.70 7.70 7.61 7.59 7.37 7.36 7.34 7.32 7.32 7.26 7.17 7.14 7.03 7.03 7.01 7.01 6.98 6.97 6.97 6.96 6.96 6.95 6.95 6.92 6.89 6.88 6.86 6.76 6.74 6.73 6.72 6.71 6.70 6.25 6.23 6.02 6.00 4.87 4.85 2.84 2.73 1.78 1.63
NO2
N
O
NO2
IIe
chemical shift [ppm]

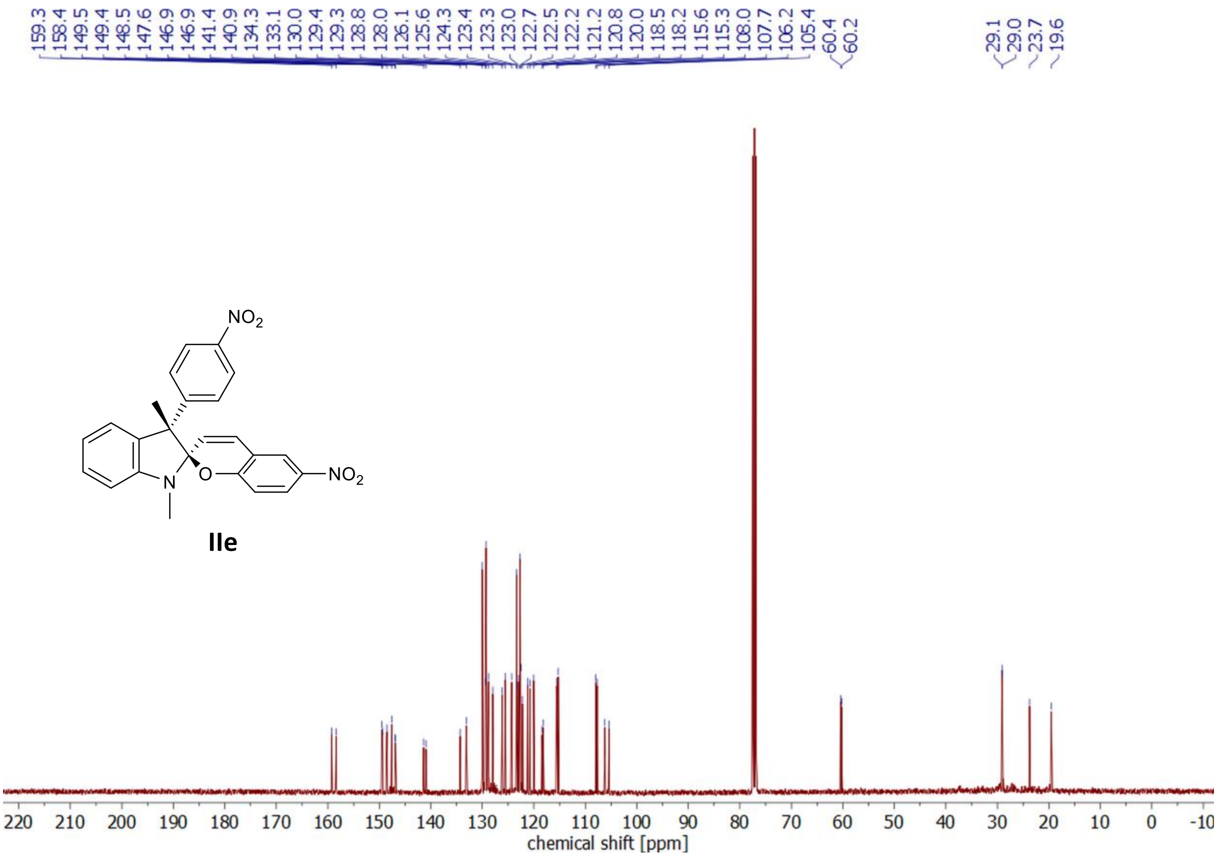
159.3 158.4 149.5 149.4 148.5 147.6 146.9 146.9 141.4 140.9 134.3 133.1 130.0 129.4 129.3 128.8 128.0 126.1 125.6 124.3 123.4 123.3 123.0 122.7 122.5 122.2 121.2 120.8 120.0 118.5 118.2 115.6 115.3 108.0 107.7 106.2 105.4 60.4 60.2
29.1 29.0 23.7 19.6
NO2
N
O
NO2
IIe
chemical shift [ppm]

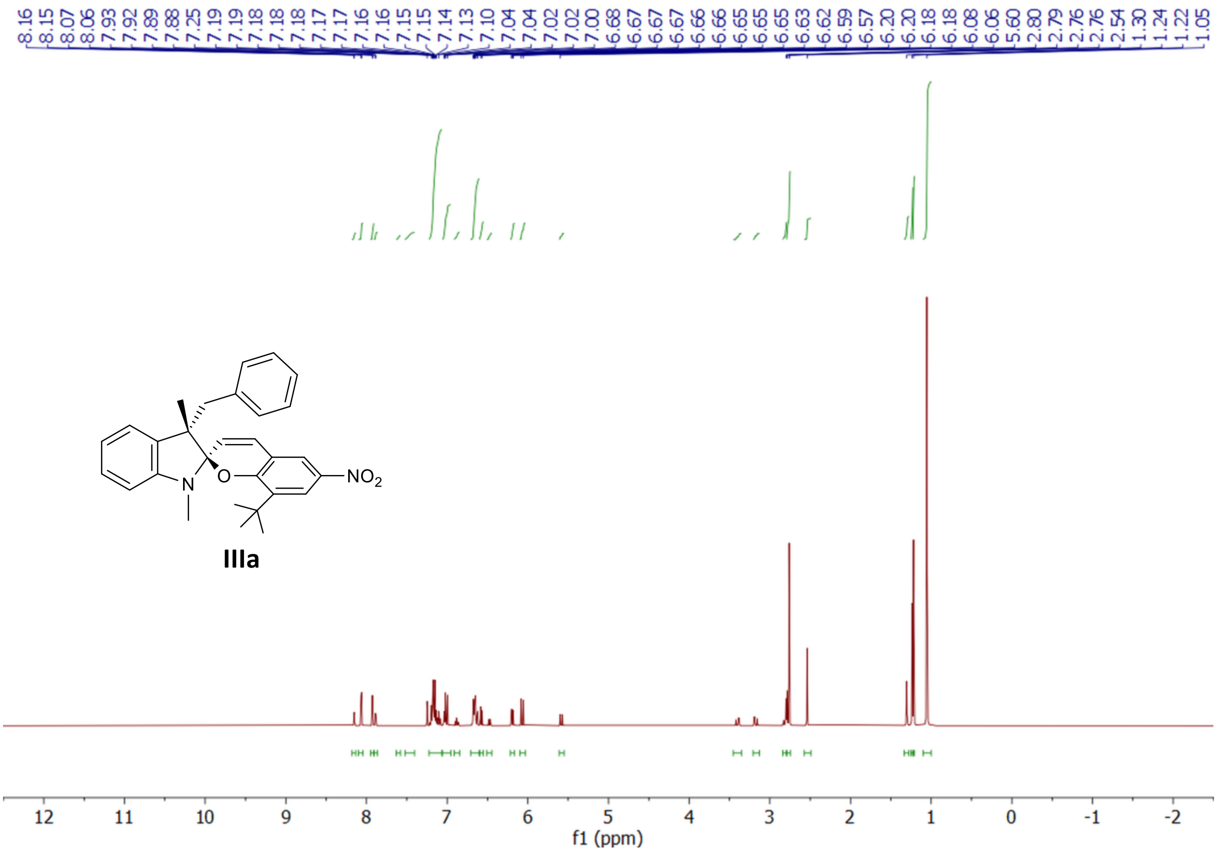
8.16
8.15
8.07
8.06
7.93
7.92
7.89
7.88
7.25
7.19
7.19
7.18
7.18
7.18
7.17
7.17
7.16
7.16
7.15
7.15
7.14
7.13
7.10
7.04
7.04
7.02
7.02
7.00
6.68
6.67
6.67
6.67
6.66
6.66
6.65
6.65
6.65
6.63
6.62
6.59
6.57
6.20
6.20
6.18
6.18
6.08
6.06
5.60
2.80
2.79
2.76
2.76
2.54
1.30
1.24
1.22
1.05
NO2
N
O
IIIa
12
11
10
9
8
7
6
5
4
3
2
1
0
-1
-2
f1 (ppm)

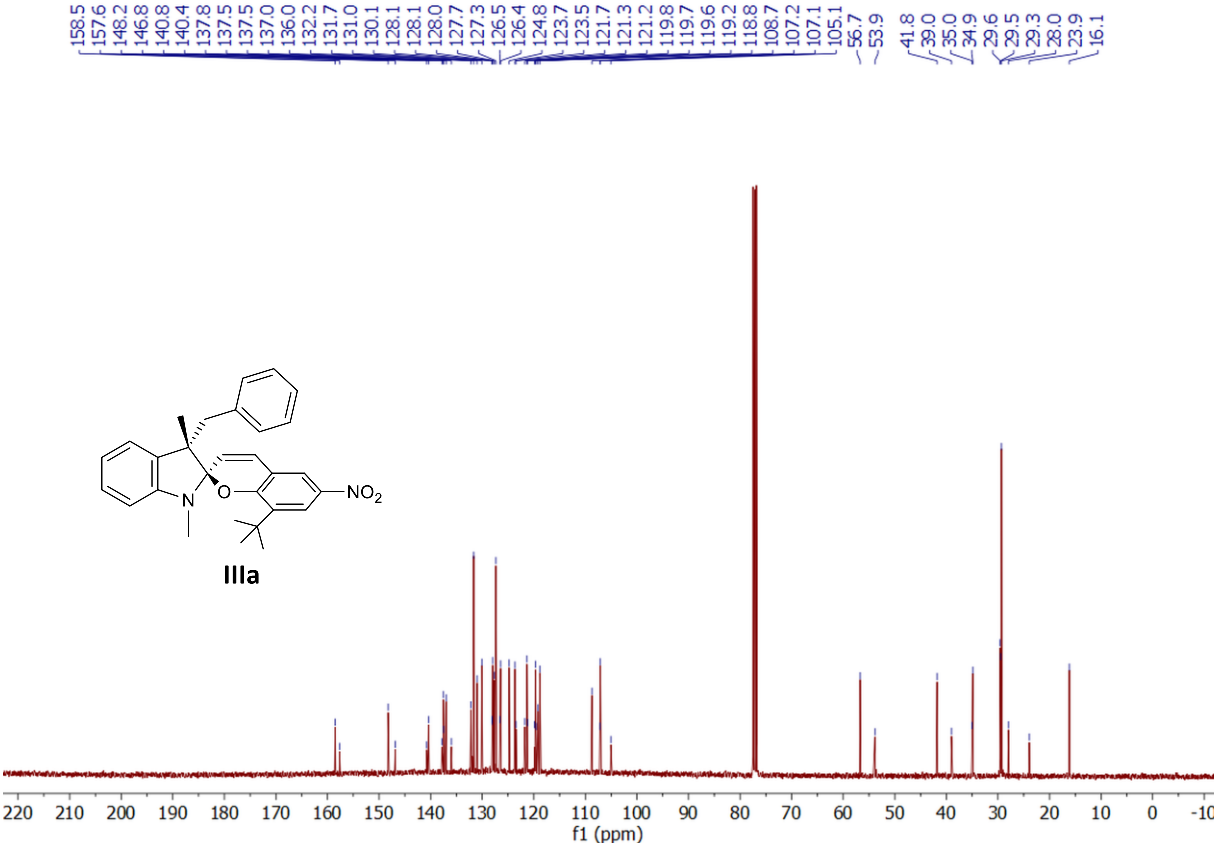
158.5
157.6
148.2
146.8
140.8
140.4
137.8
137.5
137.0
136.0
132.2
131.7
131.0
130.1
128.1
128.1
128.0
127.7
127.3
126.5
126.4
124.8
123.7
123.5
121.7
121.3
121.2
119.8
119.7
119.6
119.2
118.8
108.7
107.2
107.1
105.1
56.7
53.9
41.8
39.0
35.0
34.9
29.6
29.5
29.3
28.0
23.9
16.1
NO2
N
O
IIIa
220
210
200
190
180
170
160
150
140
130
120
110
100
90
80
70
60
50
40
30
20
10
0
-10
f1 (ppm)

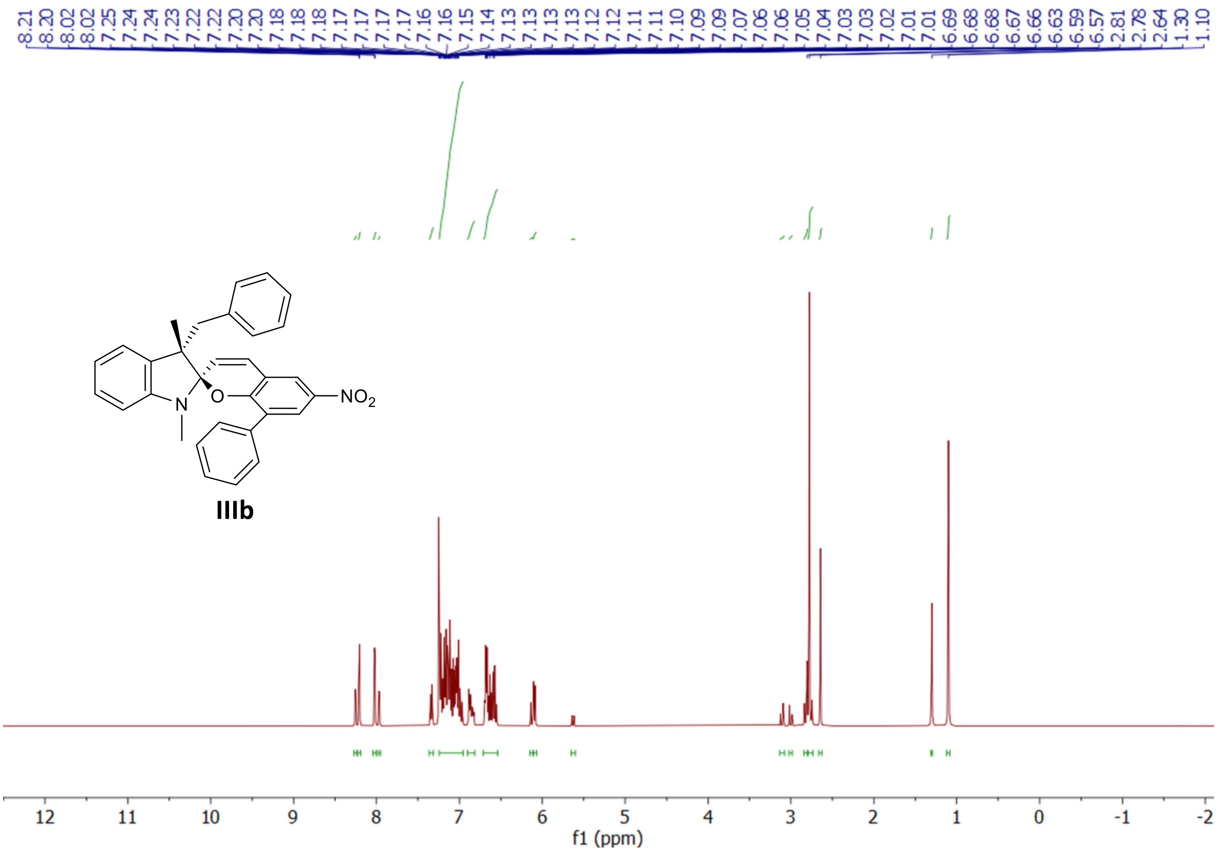
8.21 8.20 8.02 8.02 7.25 7.24 7.24 7.23 7.22 7.22 7.20 7.20 7.18 7.18 7.18 7.17 7.17 7.17 7.16 7.16 7.15 7.14 7.13 7.13 7.13 7.13 7.12 7.12 7.11 7.11 7.10 7.09 7.09 7.07 7.06 7.06 7.05 7.04 7.03 7.03 7.02 7.01 7.01 6.69 6.68 6.68 6.67 6.66 6.63 6.59 6.57 2.81 2.78 2.64 1.30 1.10
N
O
NO2
IIIb
f1 (ppm)

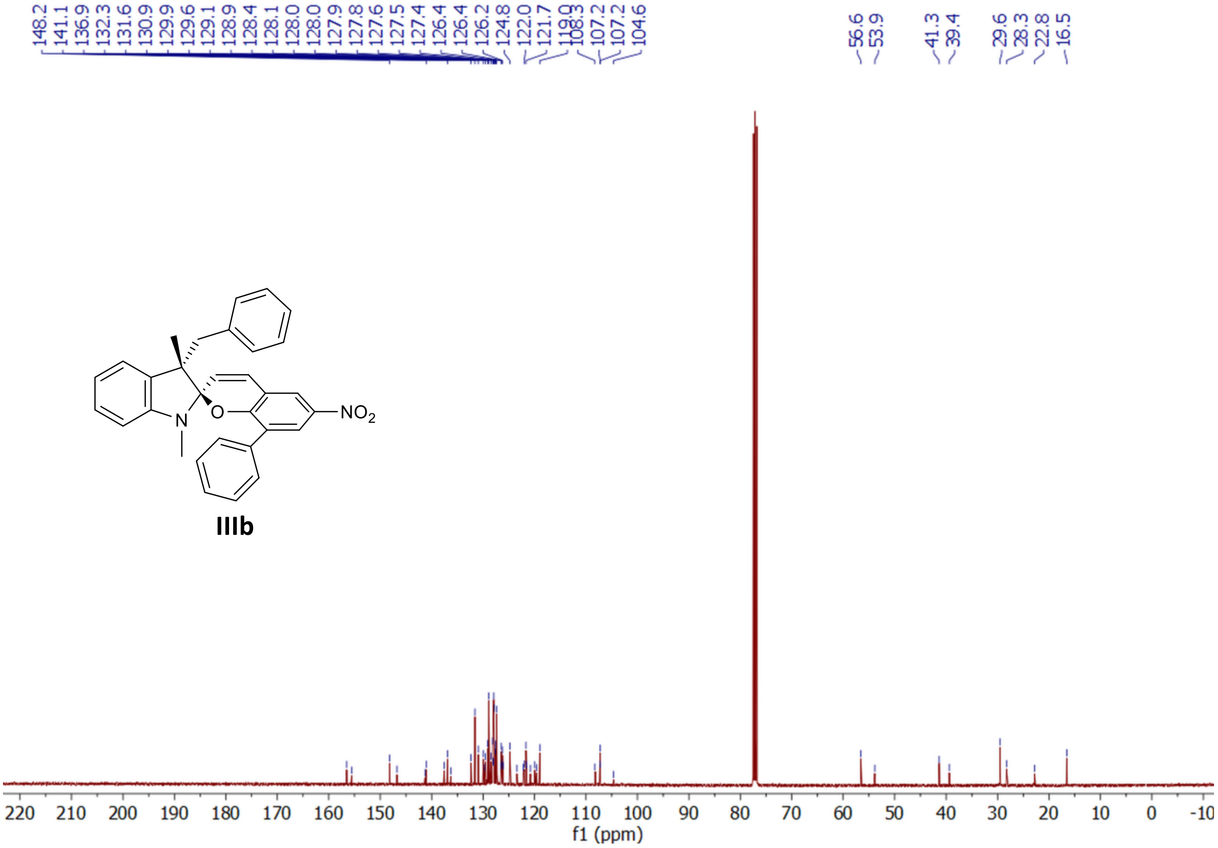
148.2 141.1 136.9 132.3 131.6 130.9 129.9 129.6 129.1 128.9 128.4 128.1 128.0 128.0 127.9 127.8 127.6 127.5 127.4 126.4 126.2 124.8 122.0 121.7 119.0 108.3 107.2 107.2 104.6
56.6 53.9
41.3 39.4
29.6 28.3 22.8 16.5
N
O
NO2
IIIb
f1 (ppm)

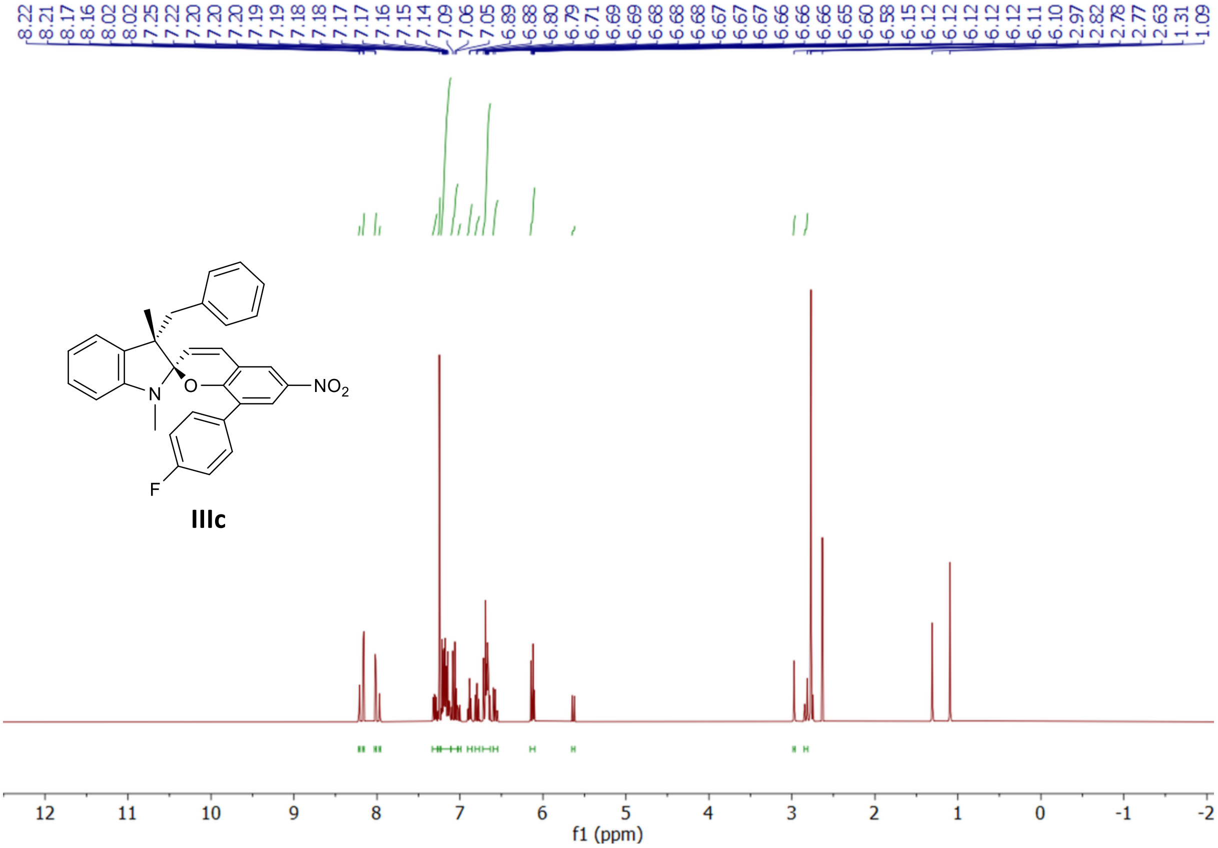
8.22 8.21 8.17 8.16 8.02 8.02 7.25 7.22 7.20 7.20 7.20 7.19 7.19 7.18 7.18 7.17 7.17 7.16 7.15 7.14 7.09 7.06 7.05 6.89 6.88 6.80 6.79 6.71 6.69 6.69 6.68 6.68 6.68 6.67 6.67 6.67 6.66 6.66 6.66 6.65 6.60 6.58 6.15 6.12 6.12 6.12 6.12 6.12 6.11 6.10 2.97 2.82 2.78 2.77 2.63 1.31 1.09
NO2
N
O
F
IIIc
f1 (ppm)

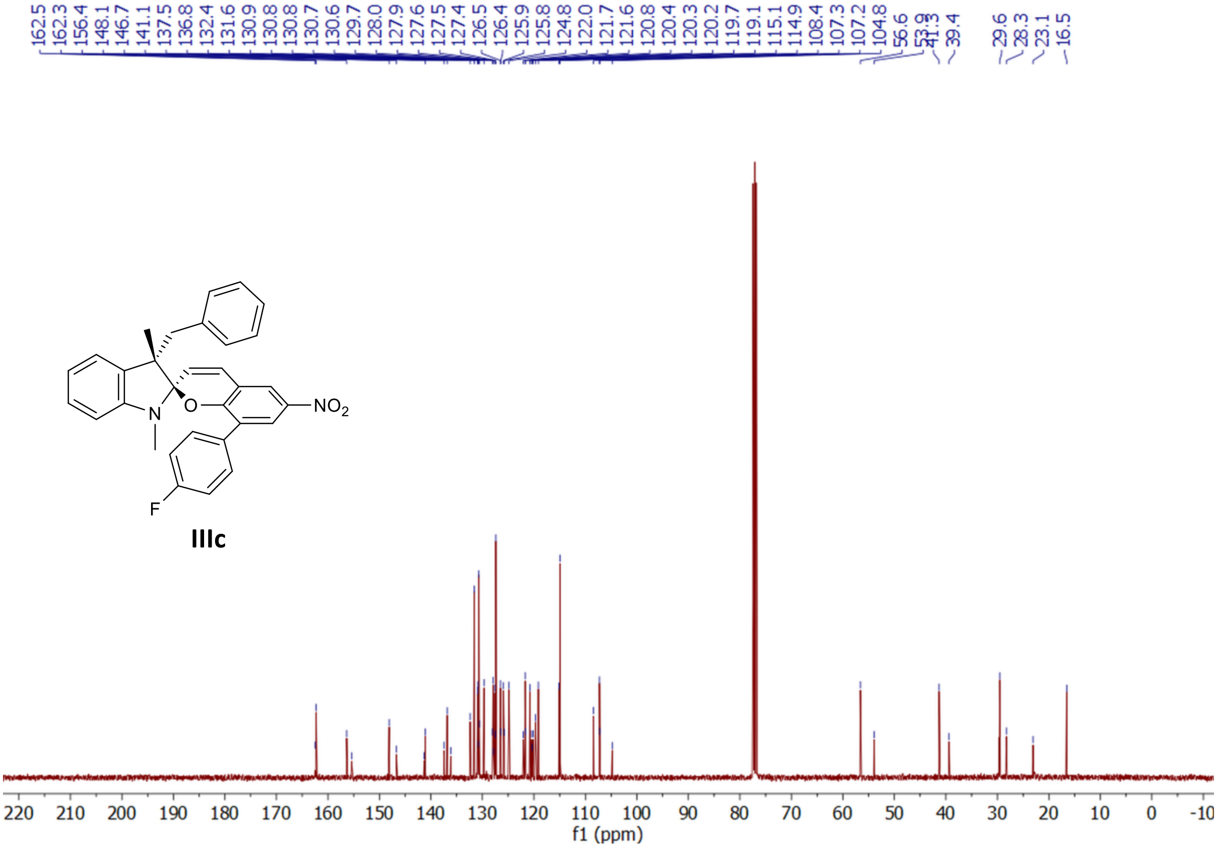
162.5 162.3 156.4 148.1 146.7 141.1 137.5 136.8 132.4 131.6 130.9 130.8 130.8 130.7 130.6 129.7 128.0 127.9 127.6 127.5 127.4 126.5 126.4 125.9 125.8 124.8 122.0 121.7 121.6 120.8 120.4 120.3 120.2 119.7 119.1 115.1 114.9 108.4 107.3 107.2 104.8 56.6 53.9 41.3 39.4 29.6 28.3 23.1 16.5
NO2
N
O
F
IIIc
f1 (ppm)

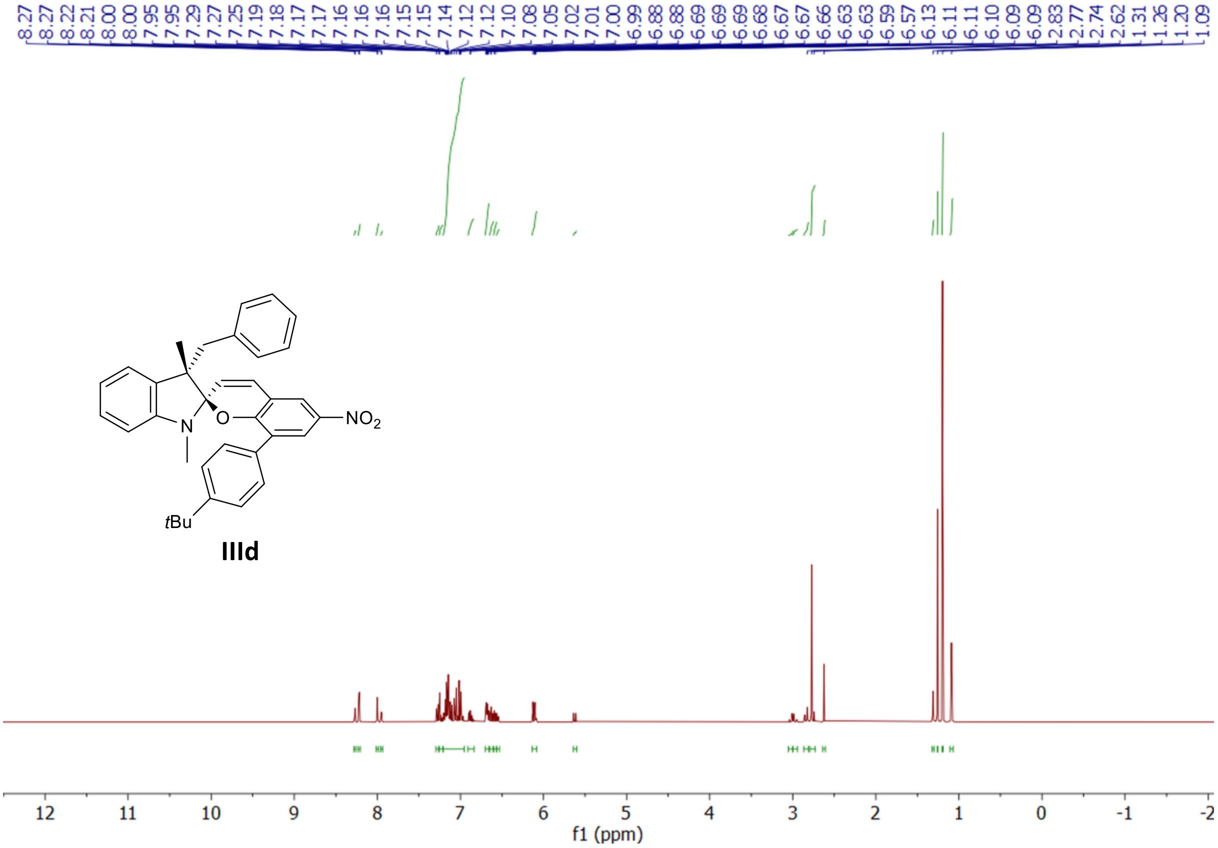
8.27 8.27 8.22 8.21 8.00 8.00 7.95 7.95 7.29 7.27 7.25 7.19 7.18 7.17 7.17 7.16 7.16 7.15 7.15 7.14 7.12 7.12 7.10 7.08 7.05 7.02 7.01 7.00 6.99 6.88 6.88 6.69 6.69 6.69 6.68 6.67 6.67 6.66 6.63 6.63 6.59 6.57 6.13 6.11 6.11 6.10 6.09 6.09 2.83 2.77 2.74 2.62 1.31 1.26 1.20 1.09
NO2
N
O
tBu
IIId
12 11 10 9 8 7 6 5 4 3 2 1 0 -1 -2
f1 (ppm)

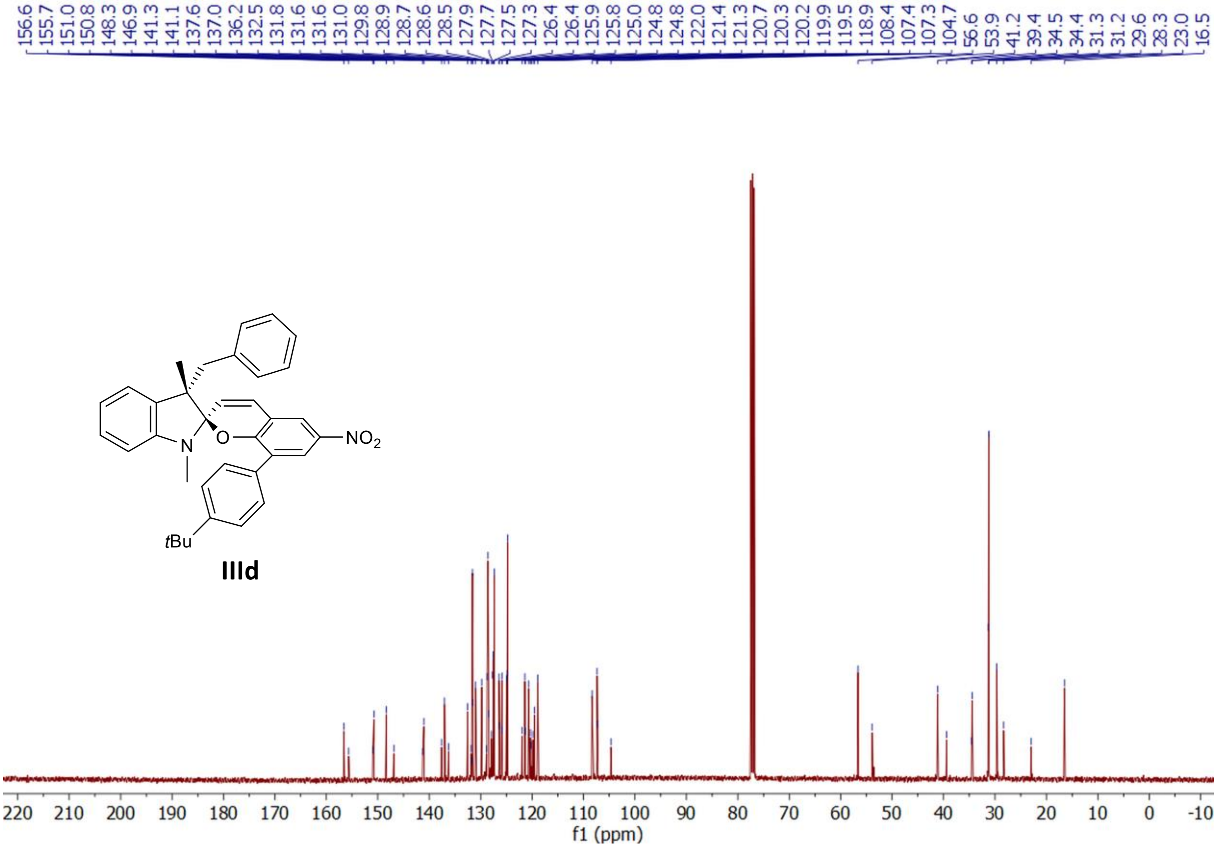
156.6 155.7 151.0 150.8 148.3 146.9 141.3 141.1 137.6 137.0 136.2 132.5 131.8 131.6 131.0 129.8 128.9 128.7 128.6 128.5 127.9 127.7 127.5 127.3 126.4 125.9 125.8 125.0 124.8 122.0 121.4 121.3 120.7 120.3 120.2 119.9 119.5 118.9 108.4 107.4 107.3 104.7 56.6 53.9 41.2 39.4 34.5 34.4 31.3 31.2 29.6 28.3 23.0 16.5
NO2
N
O
tBu
IIId
220 210 200 190 180 170 160 150 140 130 120 110 100 90 80 70 60 50 40 30 20 10 0 -10
f1 (ppm)

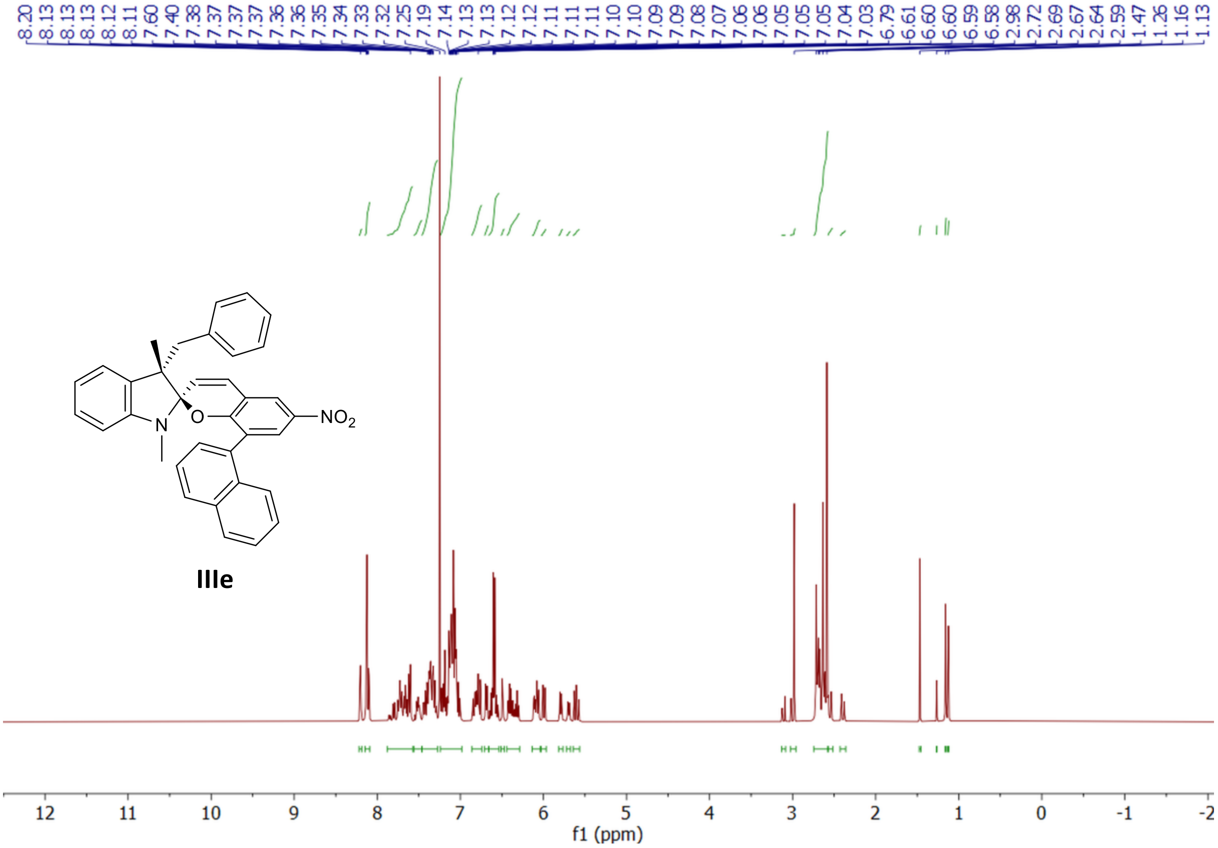
8.20 8.13 8.13 8.13 8.12 8.11 7.60 7.40 7.38 7.37 7.37 7.37 7.36 7.36 7.35 7.34 7.33 7.32 7.25 7.19 7.14 7.13 7.13 7.12 7.12 7.11 7.11 7.10 7.10 7.09 7.08 7.07 7.06 7.06 7.05 7.05 7.05 7.04 7.03 6.79 6.61 6.60 6.60 6.59 6.58 2.98 2.72 2.69 2.67 2.64 2.59 1.47 1.26 1.16 1.13
NO2
N
O
IIIe
12 11 10 9 8 7 6 5 4 3 2 1 0 -1 -2
f1 (ppm)

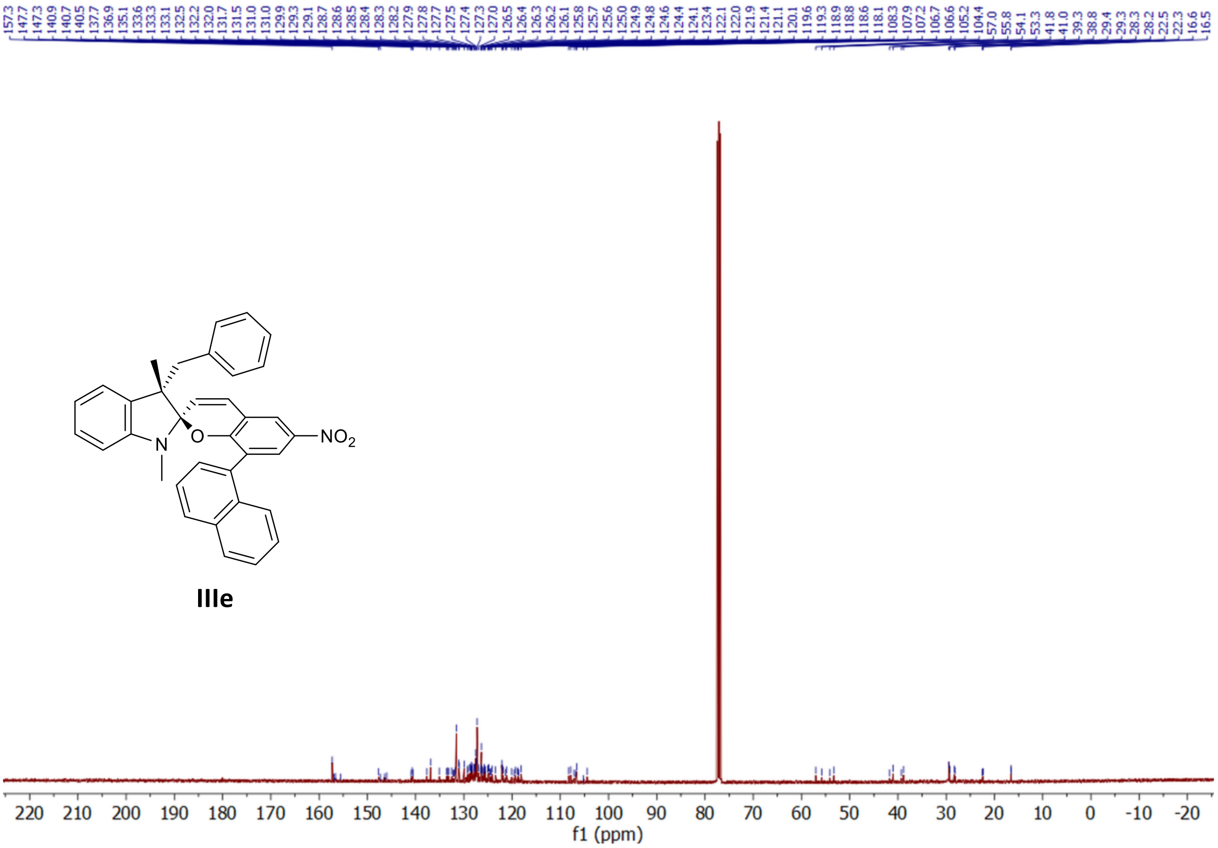
157.3 147.7 147.3 140.9 140.7 140.5 137.7 136.9 135.1 133.6 133.3 133.1 132.5 132.2 132.0 131.7 131.5 131.0 131.0 129.9 129.3 129.1 128.7 128.6 128.5 128.4 128.3 128.2 127.9 127.8 127.7 127.5 127.4 127.3 127.0 126.5 126.4 126.3 126.2 126.1 125.8 125.7 125.6 125.0 124.9 124.8 124.6 124.4 124.1 123.4 122.1 122.0 121.9 121.4 121.1 120.1 119.6 119.3 118.9 118.8 118.6 118.1 108.3 107.9 107.2 106.7 106.6 105.2 104.4 57.0 55.8 54.1 53.3 41.8 41.0 39.3 38.8 29.4 29.3 28.3 28.2 22.5 22.3 16.6 16.5
NO2
N
O
IIIe
220 210 200 190 180 170 160 150 140 130 120 110 100 90 80 70 60 50 40 30 20 10 0 -10 -20
f1 (ppm)

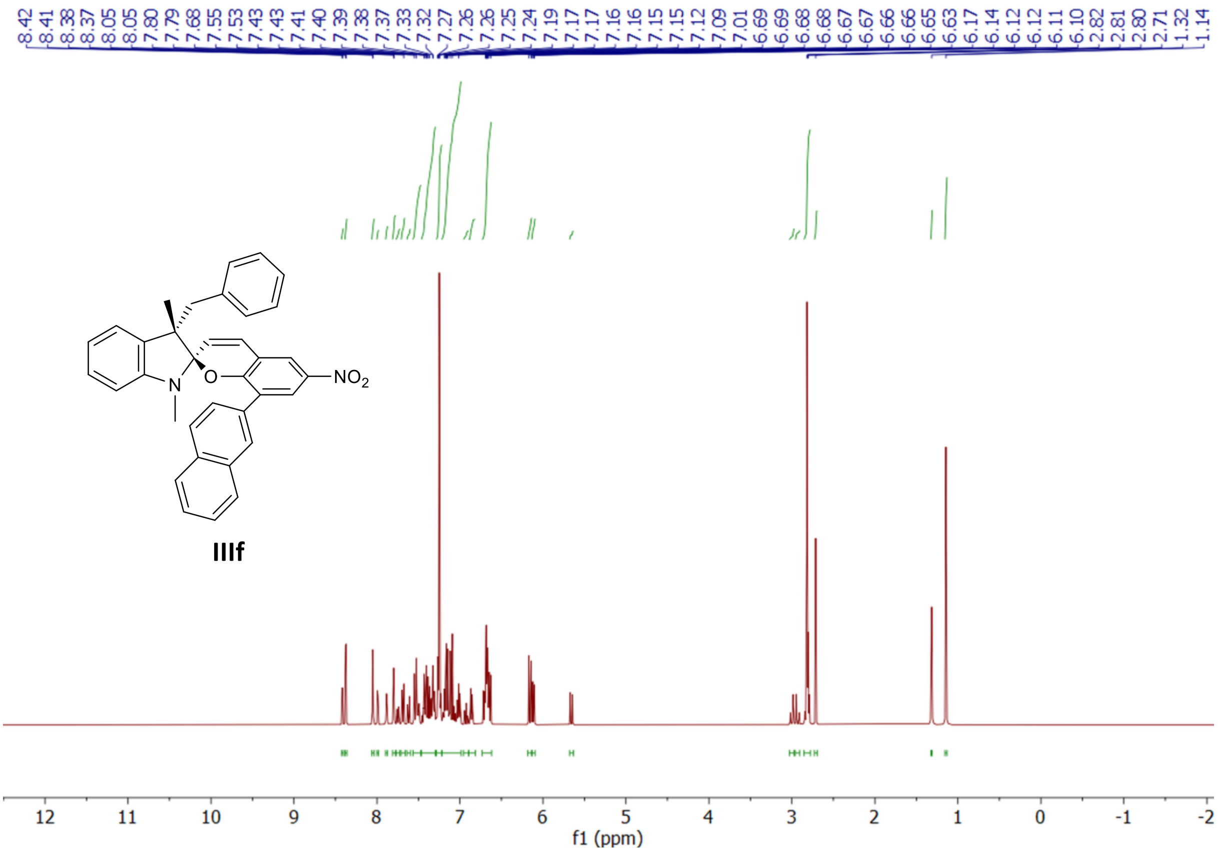
8.42 8.41 8.38 8.37 8.05 8.05 7.80 7.79 7.68 7.55 7.53 7.43 7.43 7.41 7.40 7.39 7.38 7.37 7.33 7.32 7.27 7.26 7.26 7.25 7.24 7.19 7.17 7.17 7.16 7.16 7.15 7.15 7.12 7.09 7.01 6.69 6.69 6.68 6.68 6.67 6.67 6.66 6.66 6.65 6.63 6.17 6.14 6.12 6.12 6.11 6.10 2.82 2.81 2.80 2.71 1.32 1.14
N
O
NO2
IIIf
12 11 10 9 8 7 6 5 4 3 2 1 0 -1 -2
f1 (ppm)

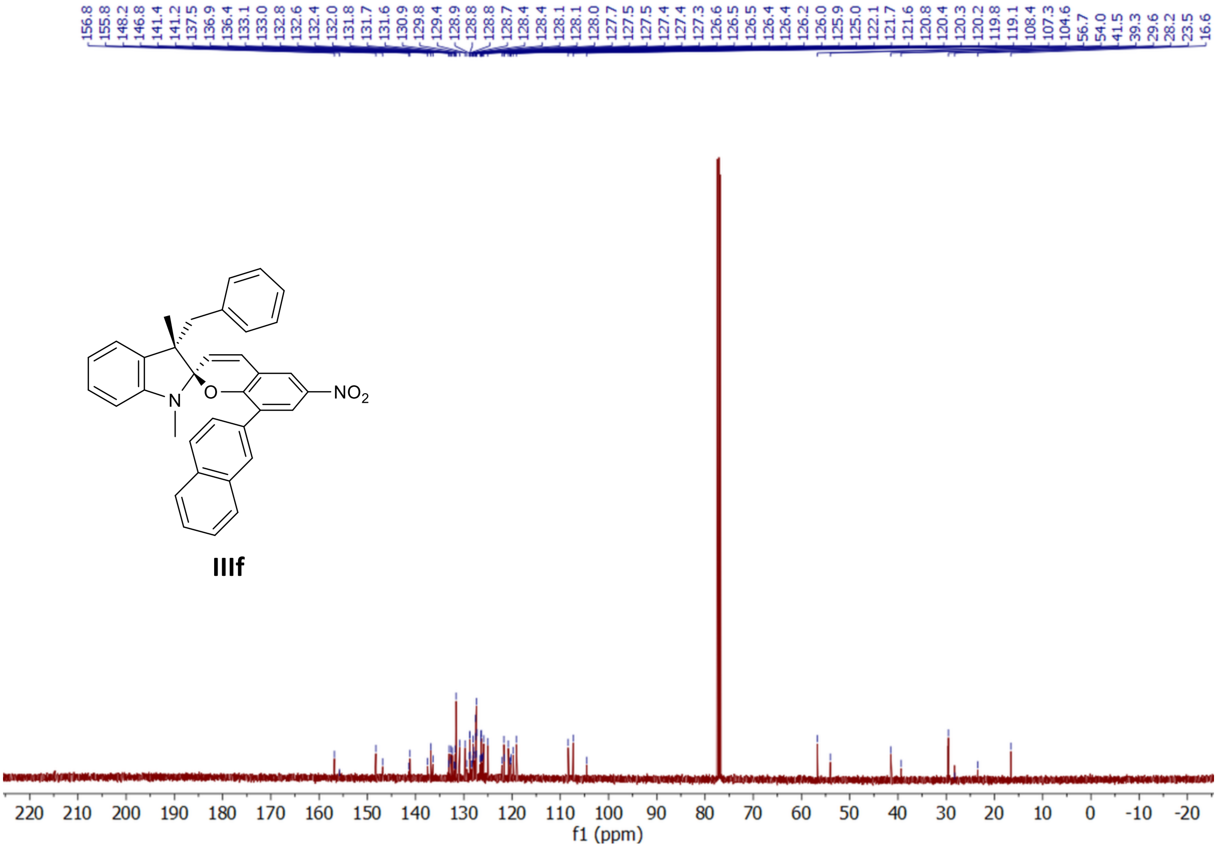
156.8 155.8 148.2 146.8 141.4 141.2 137.5 136.9 136.4 133.1 133.0 132.8 132.6 132.4 132.0 131.8 131.7 131.6 130.9 129.8 129.4 128.9 128.8 128.8 128.7 128.4 128.4 128.1 128.1 128.0 127.7 127.5 127.5 127.4 127.4 127.3 126.6 126.5 126.5 126.4 126.4 126.2 126.0 125.9 125.0 122.1 121.7 121.6 120.8 120.4 120.3 120.2 119.8 119.1 108.4 107.3 104.6 56.7 54.0 41.5 39.3 29.6 28.2 23.5 16.6
N
O
NO2
IIIf
220 210 200 190 180 170 160 150 140 130 120 110 100 90 80 70 60 50 40 30 20 10 0 -10 -20
f1 (ppm)

6. Photokinetic plots

Ia

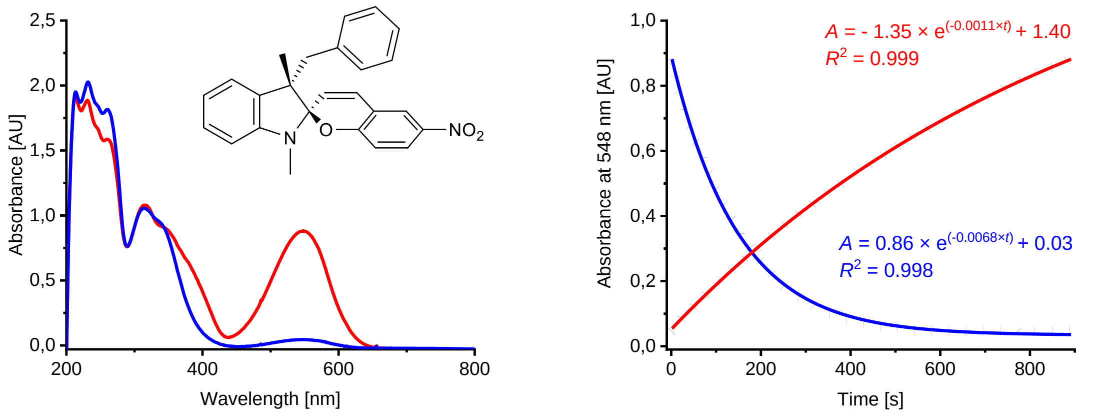

Ib

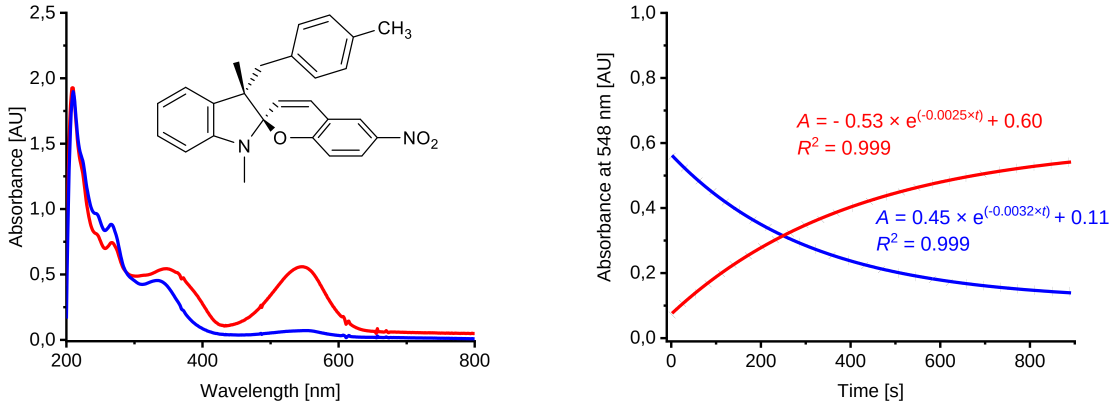

Ic

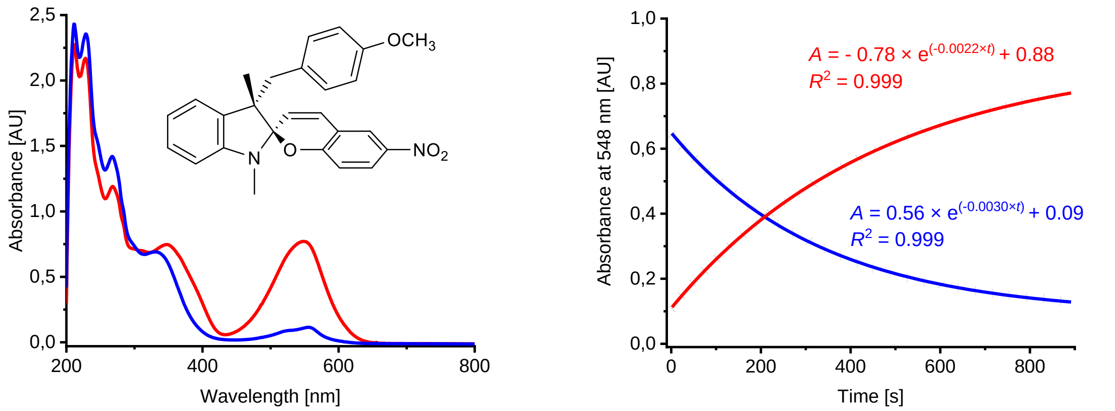

Id

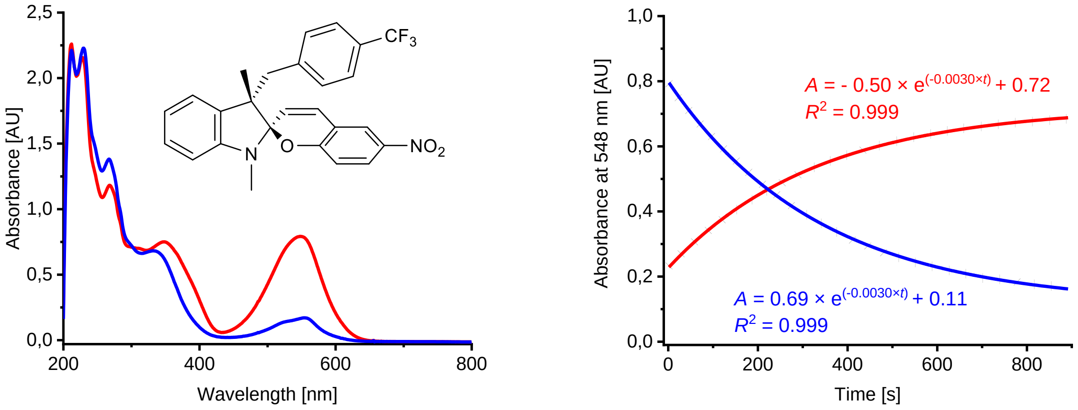
CF3
NO2
N
O
Absorbance [AU]
Wavelength [nm]
Absorbance at 548 nm [AU]
Time [s]
A = - 0.50 × e^(-0.0030×t) + 0.72
R^2 = 0.999
A = 0.69 × e^(-0.0030×t) + 0.11
R^2 = 0.999

Ie

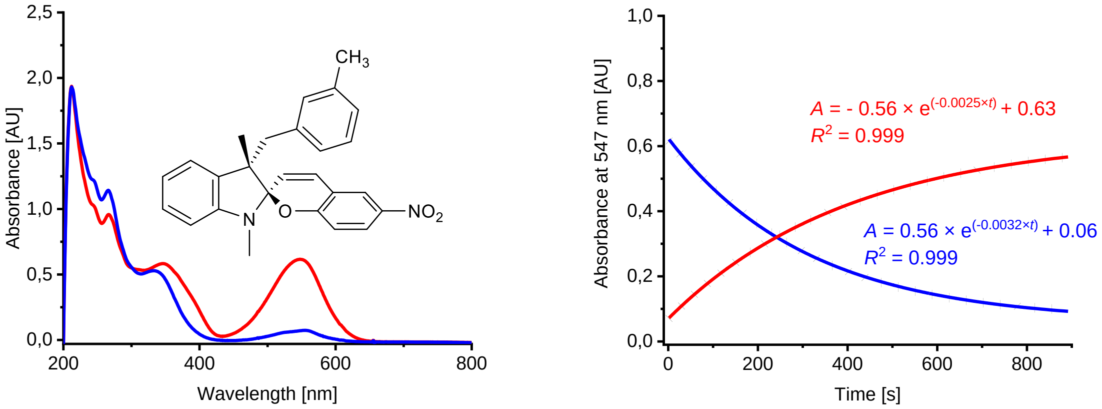
CH3
NO2
N
O
Absorbance [AU]
Wavelength [nm]
Absorbance at 547 nm [AU]
Time [s]
A = - 0.56 × e^(-0.0025×t) + 0.63
R^2 = 0.999
A = 0.56 × e^(-0.0032×t) + 0.06
R^2 = 0.999

If

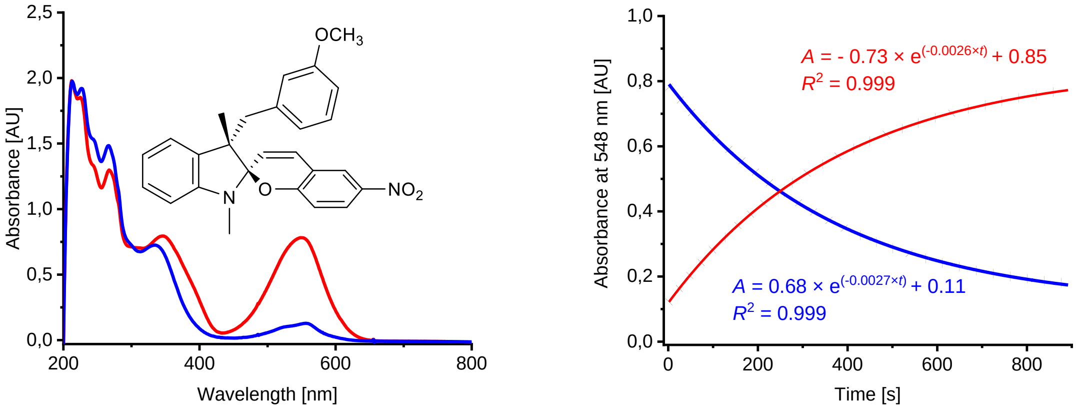
OCH3
NO2
N
O
Absorbance [AU]
Wavelength [nm]
Absorbance at 548 nm [AU]
Time [s]
A = - 0.73 × e^(-0.0026×t) + 0.85
R^2 = 0.999
A = 0.68 × e^(-0.0027×t) + 0.11
R^2 = 0.999

Ig

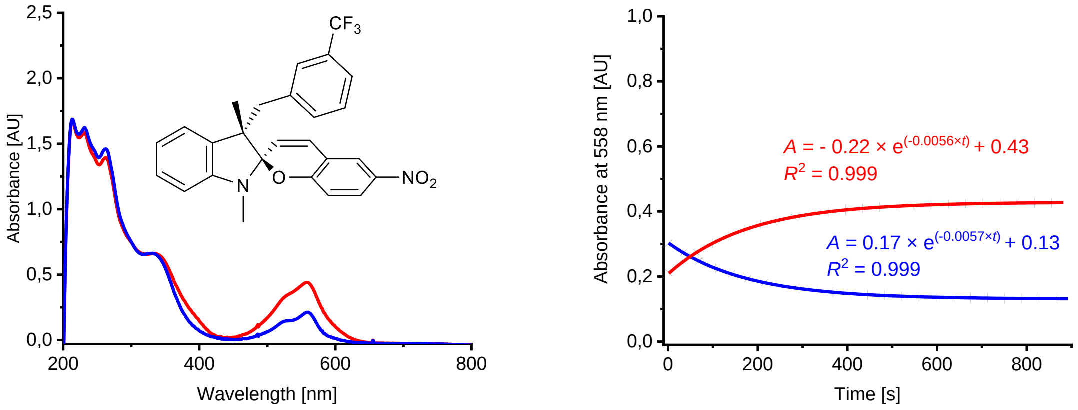

CF3
N
O
NO2
Absorbance [AU]
Wavelength [nm]
Absorbance at 558 nm [AU]
Time [s]
A = - 0.22 × e^(-0.0056×t) + 0.43
R^2 = 0.999
A = 0.17 × e^(-0.0057×t) + 0.13
R^2 = 0.999

IIa

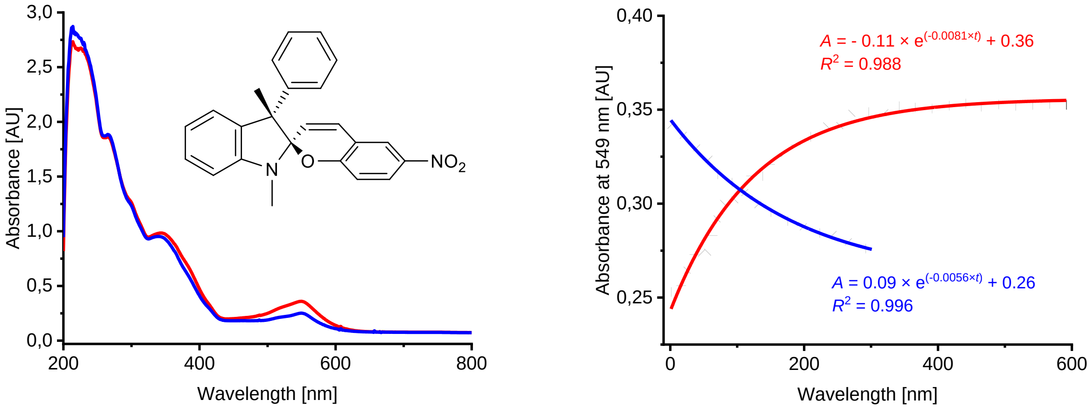

N
O
NO2
Absorbance [AU]
Wavelength [nm]
Absorbance at 549 nm [AU]
Wavelength [nm]
A = - 0.11 × e^(-0.0081×t) + 0.36
R^2 = 0.988
A = 0.09 × e^(-0.0056×t) + 0.26
R^2 = 0.996

IIb

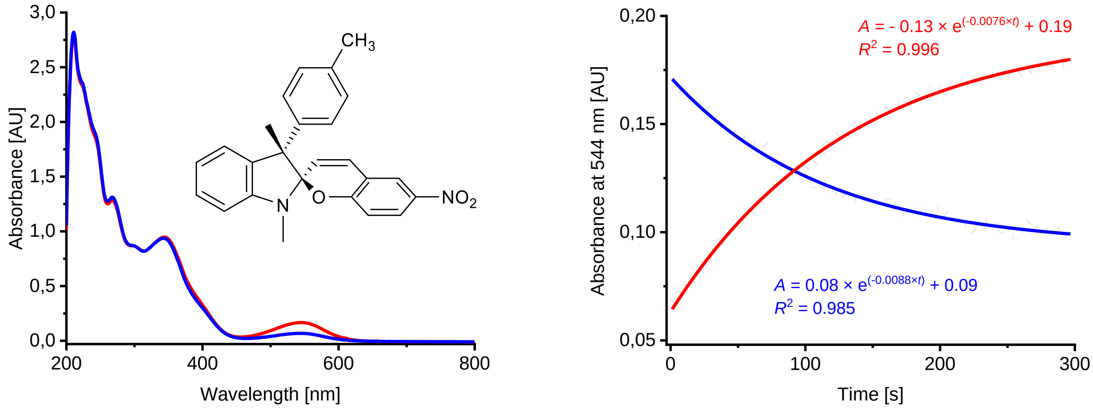

CH3
N
O
NO2
Absorbance [AU]
Wavelength [nm]
Absorbance at 544 nm [AU]
Time [s]
A = - 0.13 × e^(-0.0076×t) + 0.19
R^2 = 0.996
A = 0.08 × e^(-0.0088×t) + 0.09
R^2 = 0.985

IIc

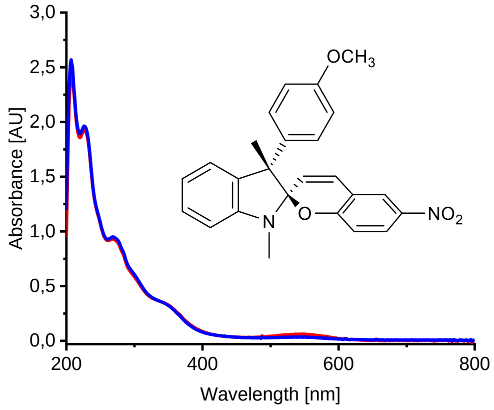
Absorbance [AU]
Wavelength [nm]
OCH3
NO2
N
O

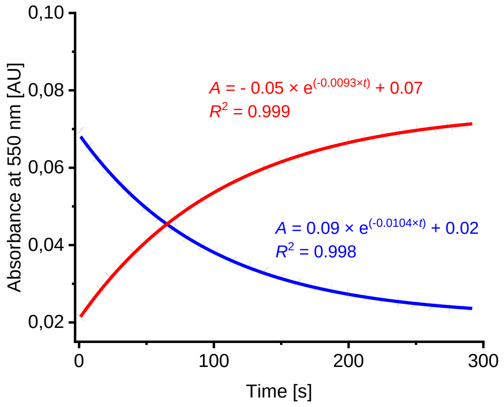
Absorbance at 550 nm [AU]
Time [s]
A = - 0.05 × e$^{(-0.0093×t)}$ + 0.07
R^2 = 0.999
A = 0.09 × e$^{(-0.0104×t)}$ + 0.02
R^2 = 0.998

IId

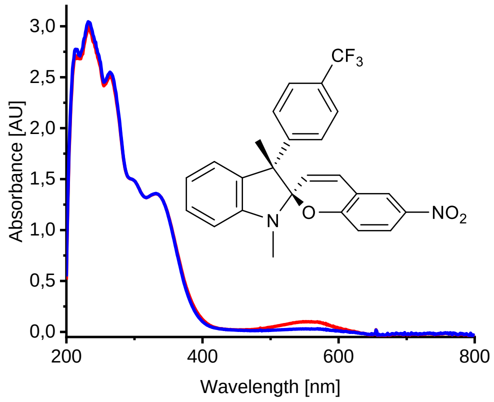
Absorbance [AU]
Wavelength [nm]
CF3
NO2
N
O

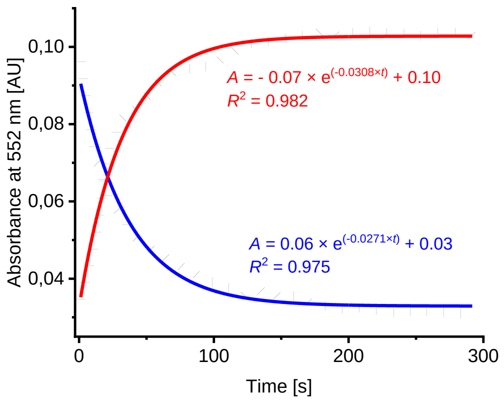
Absorbance at 552 nm [AU]
Time [s]
A = - 0.07 × e$^{(-0.0308×t)}$ + 0.10
R^2 = 0.982
A = 0.06 × e$^{(-0.0271×t)}$ + 0.03
R^2 = 0.975

IIe

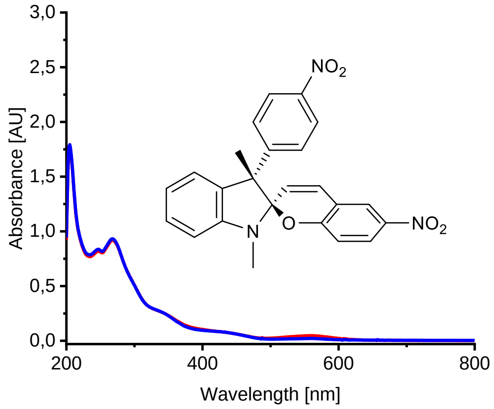
Absorbance [AU]
Wavelength [nm]
NO2
NO2
N
O

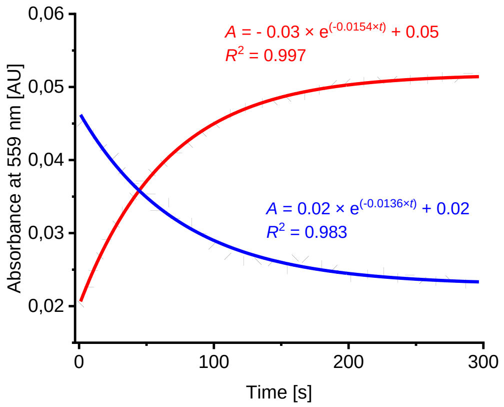
Absorbance at 559 nm [AU]
Time [s]
A = - 0.03 × e$^{(-0.0154×t)}$ + 0.05
R^2 = 0.997
A = 0.02 × e$^{(-0.0136×t)}$ + 0.02
R^2 = 0.983

IIIa

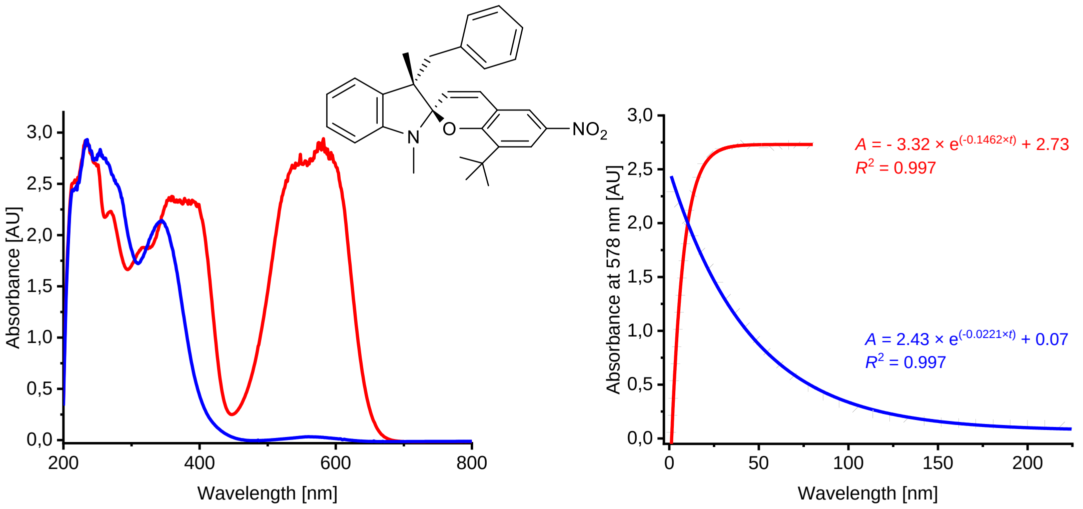

IIIb

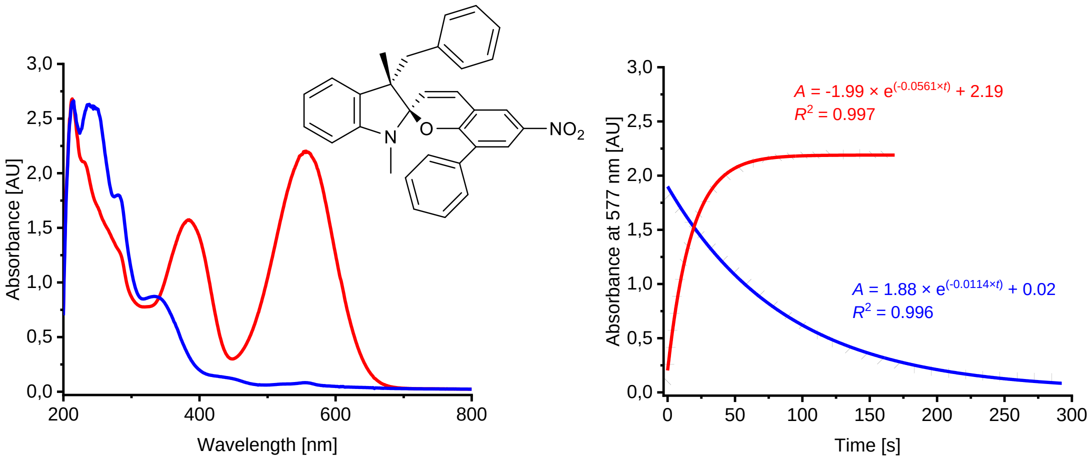

IIIc

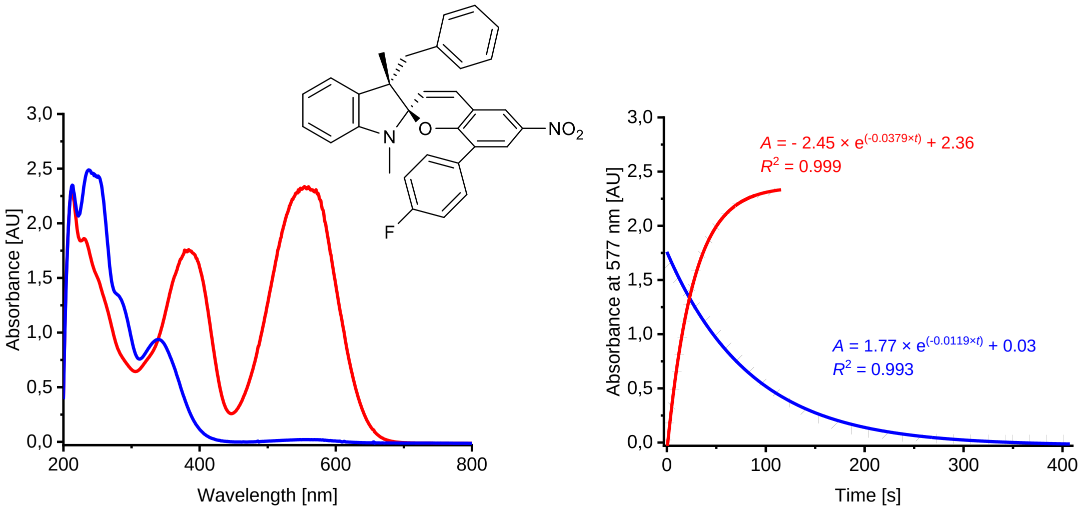

IIId

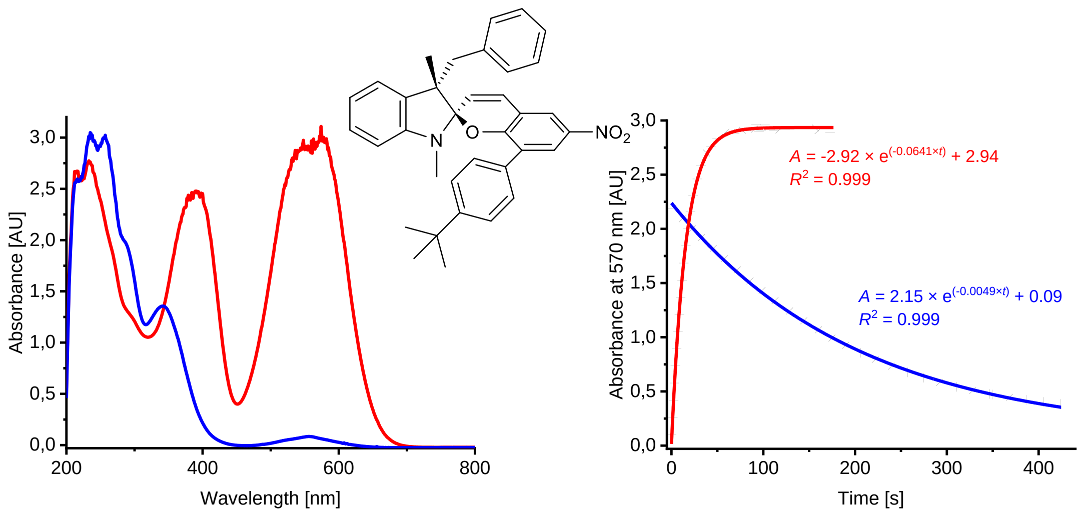

IIIe

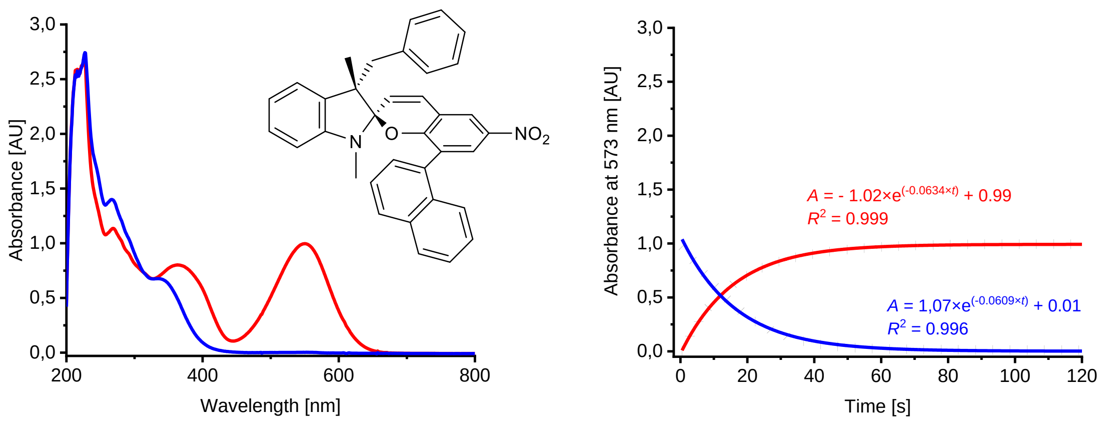

IIIf

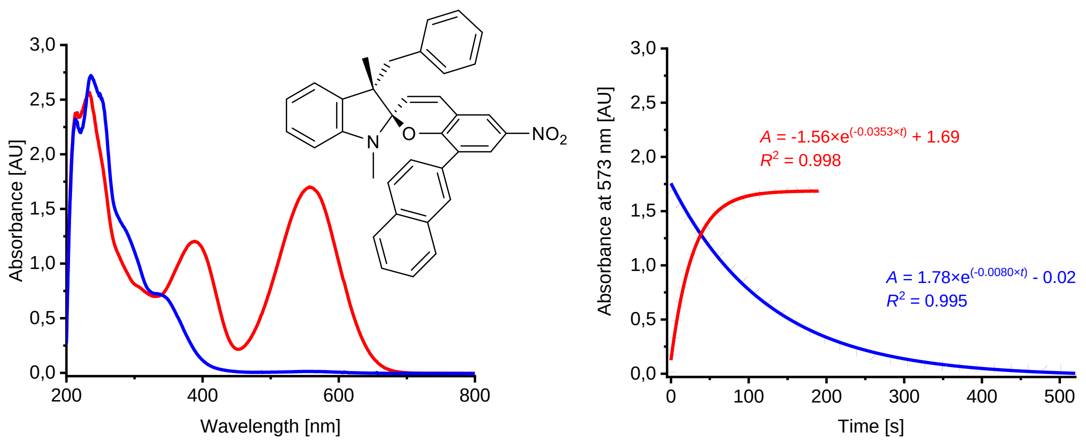

7. References

[S1] Lu, S.-M.; Bolm, C.: *Angew. Chem. Int. Ed.* **2008**, *47*, 8920.

[S2] Peters, B. B. C.; Jongcharoenkamol, J.; Krajangsri, S.; Andersson, P. G.: *Org. Lett.* **2021**, *23*, 242.

[S3] Malgorzata, Z.; Plenkiewicz, J.: *Tetrahedron Asymmetry* **2006**, *17*, 1958.

[S4] Zhu, Y.; Rawal, V. H.: *J. Am. Chem. Soc.* **2011**, *134*, 111.

[S5] Adhikari, A. A.; Radal, L.; Chisholm, J. D.: *Synlett* **2017**, *28*, 2335.

[S6] Zimmermann, T.: *J. Heterocyclic Chem.* **2000**, *37*, 1571.

[S7] Purohit, V. C.; Allwein, S. P.; Bakale, R. P.: *Org. Lett.* **2013**, *15*, 1650.

[S8] Wu, X.; Zhou, L.; Yang, R.; Guo, F.; Tang, Z.-L.; Xiao, J.: *J. Chem. Res.* **2020**, *44*, 301.

[S9] He, C.; Guo, S.; Huang, L.; Lei, A.: *J. Am. Chem. Soc.* **2010**, *132*, 8273.

[S10] Zhang, G.; Hu, X.; Chiang, C.-W.; Yi, H.; Pei, P.; Singh, A. K.; Lei, A.: *J. Am. Chem. Soc.* **2016**, *138*, 12037.

[S11] Zhang, S.-L.; Yu, Z.-L.: *J. Org. Chem.* **2016**, *81*, 57.

[S12] Kong, C.; Jana, N.; Jones, C.; Driver, T. G.: *J. Am. Chem. Soc.* **2016**, *138*, 13271.

[S13] Akimoto, R.; Tokugawa, T.; Yamamoto, Y.; Yamataka, H.: *J. Org. Chem.* **2012**, *77*, 4073.

[S14] Xu, Q.-L.; Gao, H.; Yousufuddin, M.; Ess, D. H.; Kürti, L.: *J. Am. Chem. Soc.* **2013**, *135*, 14048.

[S15] Truong, T.; Mesgar, M.; Le, K. K. A.; Daugulis, O.: *J. Am. Chem. Soc.* **2014**, *136*, 8568.

[S16] Attanasi, O. A.; Favi, G.; Filippone, P.; Forzato, C.; Giorgi, G.; Morganti, S.; Nitti, P.; Pitacco, G.; Rizzato, E.; Spinelli, D.; Valentin, E.: *Tetrahedron* **2006**, *62*, 6420.

[S17] Chen, C.-T.; Kao, J.-Q.; Salunke, S. B.; Lin, Y.-H.: *Org. Lett.* **2010**, *13*, 26.

[S18] Palatinus, L.; Chapuis, G.: *J. Appl. Cryst.* **2007**, *40*, 786.

[S19] Betteridge, P. W.; Carruthers, J. R.; Cooper, R. I.; Prout, K.; Watkin, D. J.: *J. Appl. Cryst.* **2003**, *36*, 1487.

[S20] Rohlicek, J.; Husak, M.: *J. Appl. Cryst.* **2007**, *40*, 600.

[S21] Parsons, S.; Flack, H. D.; Wagner, T.: *Acta Cryst.* **2013** *B69*, 249–259.
